# Mapping the global burden of early-onset Parkinson’s disease: socioeconomic and regional inequalities from the Global Burden of Disease Study 2021

**DOI:** 10.3389/fpubh.2025.1618533

**Published:** 2025-07-22

**Authors:** Xinyu Li, Jingpei Zhou, Wanqing Peng, Renhui Zhao, Quan Sun, Zhijuan Liu, Yanning Liu, Ziyuan Li, Ziting Huang, Yihui Zhang, Shuqiao Zhang, Xubo Hong, Zhenhu Chen, Jun Lyu, Nanbu Wang

**Affiliations:** ^1^The First School of Clinical Medicine, Guangzhou University of Chinese Medicine, Guangzhou, China; ^2^School of Acupuncture-Moxibustion and Tuina, Beijing University of Chinese Medicine, Beijing, China; ^3^The First Affiliated Hospital of Guangzhou University of Chinese Medicine, Guangzhou, China; ^4^Department of Clinical Research, The First Affiliated Hospital of Jinan University, Guangzhou, China; ^5^Key Laboratory of Regenerative Medicine of Ministry of Education, Guangzhou, China; ^6^State Key Laboratory of Traditional Chinese Medicine Syndrome, The First Affiliated Hospital of Guangzhou University of Chinese Medicine, Guangzhou, China

**Keywords:** epidemiology, public health, early-onset Parkinson’s disease, disability-adjusted life years, neurological disorders

## Abstract

**Backgrounds:**

Early-onset Parkinson’s disease (EOPD) presents a significant financial burden on healthcare systems and medical expenses. However, there has been a lack of comprehensive quantitative assessments to fully understand the extent of this burden. The Global Burden of Diseases (GBD) initiative aims to provide a standardized and thorough evaluation of these factors on a global, regional, and national scale. This study aimed to calculate the global burden of EOPD and characterize regional disparities, SDI-based inequalities, and gender differences in disease burden, with a focus on trends from 1990 to 2021.

**Methods:**

We utilized data from the GBD Study 2021 to analyze the burden of EOPD by examining factors such as incidence, prevalence, disability-adjusted life years (DALYs), and mortality rates. We focused on trends in EOPD incidence, prevalence, DALYs, and deaths from 1990 to 2021. Additionally, socio-demographic index (SDI)-related determinants that influence EOPD DALYs and characterized the disparities in EOPD burden associated with different SDI levels over the same period.

**Results:**

In EOPD, a significant increase in age-standardized rates for incidence, prevalence, and DALYs while the death rate declined. Males exhibited a higher burden than females across all metrics. Geographic disparities showed that East Asia had the highest rates of incidence and DALYs, while Andean Latin America recorded the highest prevalence. Countries with higher SDI levels, particularly China, Bolivia, and Peru, bore the greatest burden. Socioeconomic patterns suggested high-middle SDI regions experienced the highest rates of incidence and prevalence, whereas middle-SDI regions showed the highest rates of disability and mortality. Decomposition analysis revealed population growth was the primary driver of increased DALYs in middle-SDI regions. Additionally, inequality analysis indicated that countries with higher SDI levels faced a disproportionately lower burden of disease.

**Conclusion:**

This study confirms a global increase in the burden of EOPD, and indicate rising incidence and prevalence rates, an increase in DALYs, and a decline in mortality rates. A notable predominance of male cases, along with significant geographic and socioeconomic disparities. Regions with a middle SDI experience the most significant burden of disability and mortality, primarily driven by population growth. This underscores the urgent need for targeted interventions to address these inequities.

## Introduction

Parkinson’s disease (PD) is a prevalent neurodegenerative movement disorder that presents a range of clinical symptoms (1). According to the Global Burden of Diseases, Injuries, and Risk Factors Study (GBD) 2021, the global prevalence of PD has increased by 76% since 1990 (2). A notable subgroup within this global burden is early-onset Parkinson’s disease (EOPD), which is defined by onset before the age of 50 (3). EOPD presents distinct challenges for young adults and healthcare systems (4).

Although EOPD is not typically considered life-threatening, it significantly affects the healthcare system from a socioeconomic standpoint (5, 6). Compared to late-onset Parkinson’s disease (LOPD), EOPD is associated with a higher incidence of motor complications and atypical symptoms, which can result in misdiagnosis and being overlooked. Research on suicidal ideation has indicated that patients with EOPD experience a significantly higher prevalence of such thoughts compared to those with LOPD (7). In addition to the risks of motor complications, atypical clinical manifestations, and increased suicidal ideation, EOPD patients face various unique challenges that deserve attention. These challenges include delays in diagnosis, complexities in management, workplace stigma, and the psychosocial impact of living with a chronic illness.

Nonetheless, epidemiological studies on EOPD are currently insufficient (8, 9). The majority of existing research has been carried out in the past and has concentrated on particular geographical regions. In this study, we analyzed the data according to the World Health Organization (WHO) regional classifications and social demographic index (SDI) quintiles to ensure global comparability. It is imperative to have up-to-date assessments of the worldwide, regional, and national prevalence of EOPD, as well as trends over time, with a specific focus on measuring regional disparities and SDI-based inequalities. These analyses aim to inform evidence-based health policies, strategies, and resource distribution for this disorder.

The study aims to analyze global, regional, and national trends in EOPD burden (1990–2021) and characterize geographic disparities (by WHO regions), socioeconomic inequalities (by SDI quintiles), and gender differences, providing insights for targeted interventions. The analysis was performed on a global, regional, and national scale, with detailed stratification by WHO regions and SDI categories to highlight disparities in disease burden and healthcare access. The research identified significant inequalities in the burden of this condition related to sex and sociodemographic development, providing valuable insights for healthcare institutions, policymakers, and the general public. The study was conducted as part of the GBD Collaborator Network, in accordance with the established GBD study protocol.

## Methods

### Study population and data collection

The study population and data collection for this research involved gathering information on the incidence, prevalence, Disability-Adjusted Life Years (DALYs), and mortality rates of EOPD at global, regional, and national levels, drawing from the Global Burden of Disease Study 2021 (GBD 2021^1^). EOPD is clinically defined as a diagnosis made before the age of 50 (3). However, the GBD Study 2021 presents data based on current age groups, such as 40–54 years, rather than on the age of onset. To estimate the burden of EOPD, we have selected the 40–54 age group as a practical proxy. This approach aligns with previous GBD analyses of early-onset conditions and is supported by epidemiological studies (8, 10).

This study offers a comprehensive analysis of 369 diseases and 87 risk factors across 204 countries and territories. The countries and territories were categorized into 46 regions based on epidemiological similarities and geographical proximity. Additionally, they were further grouped into five categories according to the SDI.

SDI incorporates income per capita, educational attainment, and total fertility rate, capturing various aspects of development that purely income-based metrics overlook. We chose SDI over income level for three main reasons: first, Multidimensionality: Income alone fails to reflect the effects of education on health literacy or changes in fertility-related demographics. For instance, regions with similar income levels may differ in educational attainment, resulting in varying burdens of EOPD. Second, GBD Framework Consistency: SDI is the standard socioeconomic metric used in GBD studies, allowing for better comparability across regions in previous EOPD research (11–13). Third, Burden Driver Discrimination: Our decomposition analysis revealed that trends stratified by SDI show different drivers of disease burden. For example, population growth significantly impacted DALYs in middle-SDI regions, while aging was a more significant factor in high-SDI regions. These nuances cannot be captured through income-level analysis.

The data utilized in this study can be accessed through the Global Health Data Exchange query tool,^2^ with data analysis finalized on March 25, 2024. Ethical oversight for GBD research was provided by the Institutional Review Board of the University of Washington, which granted a waiver of informed consent. Further details on ethical standards can be found on the official website (see text footnote 1).

### Statistical analysis

Prior research has thoroughly explained the methodologies used in the GBD Study 2021 (12, 13). This study computed a 95% uncertainty interval (UI) for each variable, confirming that all age-standardized rates (incidence, prevalence, DALYs, and death rates) are expressed per 100,000 population. Significance was determined using two-sided tests with a threshold of *p* < 0.05. The analysis of age-standardized incidence, prevalence, DALYs, and death rates was conducted and stratified by global, regional, national, gender, and SDI categories.

Using the Joint Command Line version, a join-point regression analysis was performed to evaluate trends in the burden of EOPD. This software monitors data trends over time and applies the simplest feasible model by linking multiple line segments on a logarithmic scale. Average annual percentage changes (AAPC) were computed to analyze these trends. AAPC represents a geometrically weighted mean of the various annual percentage changes derived from the join-point trend analysis, with weights corresponding to the duration of each period within the designated time frame (14). Additionally, the linear regression model successfully calculated a 95% confidence interval for the AAPC value, indicating a significant relationship between the AAPC value and the corresponding age-standardized rate (ASR) (10).

Decomposition analysis was used to visually demonstrate the effects of aging, population dynamics, and epidemiological changes on DALY variations from 1990 to 2021, with epidemiological changes accounting for adjustments related to age and population-specific mortality and morbidity rates (15).

We conducted a frontier analysis to further investigate the relationship between the burden of EOPD and sociodemographic development. The difference between the observed age-standardized DALYs rate in a country and its frontier indicates a potential health gain that could be achieved considering the current level of development in that country or region. By quantifying the gap between the observed DALYs and this frontier, we can identify unexploited health gains, which is essential for our goal of finding areas where intervention is needed. We used nonparametric data envelope analysis and referenced detailed descriptions in previous studies (16).

Our study assessed distributive inequality in the burden of EOPD using the slope index of inequality and health inequality concentration index, which are commonly used metrics for measuring absolute and relative gradient inequality (17, 18). The slope index of inequality was calculated through a regression analysis of country-level age-standardized years of life lost (YLL) rates due to EOPD across all age groups. This analysis utilized a relative social position scale, determined by the midpoint of cumulative class intervals of the population, ranked by gross domestic product (GDP) per capita. To address heteroskedasticity, a weighted regression model was employed, and a logarithmic transformation of the relative social position value was used to correct for non-linearity resulting from marginal utility. Additionally, the Health Inequality Concentration Index was calculated by fitting a Lorenz concentration curve to the observed cumulative relative distributions of the population ranked by income and the burden of disease measured in Years of Life Lost. This was followed by the numerical integration of the area under the curve. These indices were derived from country-level data on age-standardized YLL rates and socioeconomic indicators, such as GDP per capita, obtained from the Global Burden of Disease Study 2021 (12, 13).

All statistical analyses and graphical representations were conducted using R version 4.5.0 and GraphPad Prism 8.

## Results

### Global early-onset Parkinson’s disease burden

**Figure 1:**
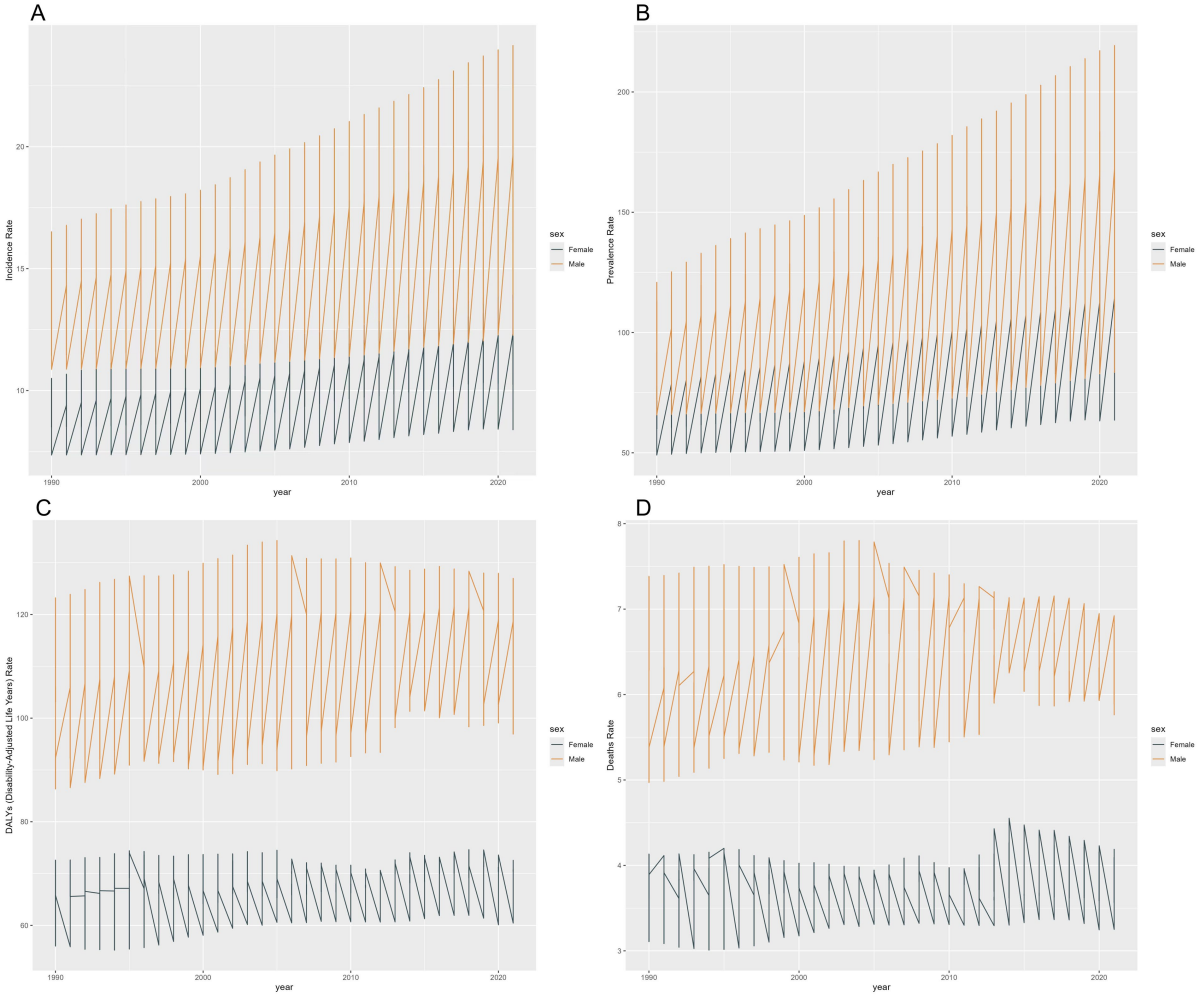
Sex-specific disparities in the burden of EOPD across various metrics, 1990–2021. **(A)** Age-standardized incidence rate; **(B)** age-standardized prevalence rate; **(C)** age-standardized DALY rate; **(D)** age-standardized death rate.

In 2021, global estimates indicated there were approximately 133,052 new cases and 909,753 prevalent cases of EOPD. This corresponds to 351,260 DALYs per 100,000 population attributed to the condition. Additionally, the same year recorded about 5,105 EOPD-related deaths. Sex-specific analysis showed a consistent male predominance across all burden metrics. In 2021, the male-to-female ratios were 1.72 for incidence, 1.65 for prevalence, 1.69 for DALYs, and 1.81 for mortality (Table 1; Figure 1).

**Table 1:** The global incidence, prevalence, disability-adjusted life-years, and deaths of early onset Parkinson’s disease in 2021 for both sexes, sex-specific and all SDI, with AAPC from 2009 and 2021.

Variables	Location	1990		2021		AAPC % (95% CI) 1990–2019
		Cases (95% UI)	ASR (per 100, 000) (95% UI)	Cases (95% UI)	ASR (per 100, 000) (95% UI)	
Incidence	Global	35441.90 (50248.49 to 22702.09)	4.91 (6.96 to 3.14)	133051.85 (182904.58 to 91891.59)	9.17 (12.63 to 6.32)	2.02 (1.81 to 2.23)
	Female	15560.22 (22000.88 to 9829.93)	4.39 (6.21 to 2.76)	54086.32 (74602.55 to 36954.93)	7.47 (10.32 to 5.10)	1.68 (1.56 to 1.8)
	Male	19881.68 (28207.25 to 12833.81)	5.41 (7.69 to 3.49)	78965.52 (108716.71 to 54933.75)	10.87 (14.98 to 7.55)	2.24 (2.05 to 2.43)
	High SDI	7500.99 (10581.71 to 4815.34)	4.75 (6.70 to 3.04)	14885.68 (19633.44 to 10645.56)	6.38 (8.46 to 4.54)	0.95 (0.92 to 0.98)
	High-middle SDI	8994.53 (12637.78 to 5781.47)	5.37 (7.59 to 3.42)	39402.08 (54233.28 to 27439.12)	12.99 (17.94 to 9.01)	2.88 (2.63 to 3.12)
	Middle SDI	10978.25 (15583.06 to 7041.42)	5.11 (7.26 to 3.28)	55518.14 (76423.31 to 38586.21)	11.06 (15.26 to 7.67)	2.39 (2.14 to 2.63)
	Low-middle SDI	6051.41 (8755.24 to 3807.48)	4.57 (6.60 to 2.88)	17872.09 (25488.05 to 11745.83)	6.10 (8.69 to 4.01)	0.9 (0.8 to 1)
	Low SDI	1883.93 (2778.19 to 1124.14)	3.78 (5.57 to 2.26)	5312.80 (7742.36 to 3252.07)	4.38 (6.35 to 2.68)	0.47 (0.43 to 0.51)
Prevalence	Global	266610.27 (369684.20 to 186707.43)	36.97 (51.18 to 25.92)	909753.30 (1228381.75 to 666184.39)	62.47 (84.42 to 45.69)	1.66 (1.55 to 1.78)
	Female	112145.11 (155156.21 to 77703.35)	31.64 (43.72 to 21.95)	366765.10 (500896.75 to 261723.21)	50.42 (68.95 to 35.96)	1.45 (1.35 to 1.55)
	Male	154465.16 (213026.52 to 109337.58)	42.12 (58.00 to 29.84)	542988.20 (727112.67 to 400303.72)	74.48 (99.78 to 54.86)	1.81 (1.68 to 1.93)
	High SDI	56168.94 (77933.64 to 39232.86)	35.60 (49.30 to 24.89)	99726.51 (131274.51 to 73280.21)	42.57 (56.16 to 31.24)	0.58 (0.55 to 0.62)
	High-middle SDI	61553.40 (85736.37 to 42356.85)	36.72 (51.11 to 25.30)	247345.35 (337049.72 to 178123.71)	80.37 (109.75 to 57.80)	2.51 (2.38 to 2.64)
	Middle SDI	82855.55 (113800.19 to 58397.74)	38.70 (53.04 to 27.32)	376585.13 (506444.85 to 274363.18)	74.47 (100.27 to 54.19)	2.19 (1.92 to 2.45)
	Low-middle SDI	49776.70 (68974.53 to 35189.84)	37.69 (52.14 to 26.69)	141231.87 (194394.18 to 101320.70)	48.31 (66.42 to 34.70)	0.78 (0.71 to 0.85)
	Low SDI	16011.43 (22489.76 to 11067.92)	32.22 (45.20 to 22.31)	44410.34 (61736.39 to 30856.55)	36.73 (50.94 to 25.57)	0.41 (0.36 to 0.46)
Disability-adjusted life-years	Global	161635.72 (186792.25 to 139607.80)	22.42 (25.90 to 19.38)	351260.21 (422771.72 to 290342.77)	24.17 (29.10 to 19.98)	0.29 (0.19 to 0.38)
	Female	65160.69 (78261.20 to 53478.94)	18.40 (22.09 to 15.11)	133980.00 (164876.87 to 109480.14)	18.43 (22.69 to 15.05)	−0.01 (−0.13 to 0.12)
	Male	96475.03 (112063.02 to 82616.93)	26.31 (30.56 to 22.54)	217280.21 (262099.37 to 178179.79)	29.90 (36.06 to 24.52)	0.46 (0.37 to 0.54)
	High SDI	27452.61 (32242.87 to 23820.64)	17.46 (20.48 to 15.16)	43784.03 (50994.44 to 37804.68)	18.67 (21.78 to 16.11)	0.22 (0.13 to 0.32)
	High-middle SDI	38868.80 (44945.20 to 33219.64)	23.16 (26.80 to 19.78)	79337.10 (101844.12 to 63275.00)	25.96 (33.32 to 20.73)	0.4 (0.32 to 0.49)
	Middle SDI	56363.22 (64871.57 to 47767.80)	26.36 (30.33 to 22.34)	136263.92 (166052.97 to 111012.98)	27.07 (33.00 to 22.07)	0.12 (0.03 to 0.2)
	Low-middle SDI	28195.26 (33651.67 to 23568.84)	21.44 (25.57 to 17.93)	67159.62 (80359.99 to 56070.27)	22.99 (27.50 to 19.21)	0.22 (0.15 to 0.29)
	Low SDI	10624.13 (12993.33 to 8548.99)	21.44 (26.23 to 17.24)	24490.98 (29838.15 to 19569.14)	20.25 (24.64 to 16.20)	−0.16 (−0.31 to −0.02)
Deaths	Global	2900.73 (3207.04 to 2548.66)	0.40 (0.45 to 0.35)	5105.23 (5679.35 to 4570.92)	0.35 (0.39 to 0.31)	−0.46 (−0.55 to −0.37)
	Female	1156.49 (1355.39 to 907.74)	0.33 (0.38 to 0.26)	1888.55 (2309.92 to 1566.05)	0.26 (0.32 to 0.21)	−0.8 (−0.96 to −0.65)
	Male	1744.24 (1989.11 to 1529.39)	0.48 (0.54 to 0.42)	3216.68 (3671.37 to 2788.87)	0.44 (0.50 to 0.38)	−0.24 (−0.28 to −0.19)
	High SDI	452.41 (465.72 to 441.41)	0.29 (0.30 to 0.28)	689.41 (721.38 to 659.37)	0.29 (0.31 to 0.28)	0.09 (0 to 0.18)
	High-middle SDI	711.60 (797.17 to 624.25)	0.42 (0.47 to 0.37)	1004.21 (1196.77 to 861.41)	0.33 (0.39 to 0.28)	−0.77 (−0.88 to −0.66)
	Middle SDI	194.66 (241.81 to 154.64)	0.49 (0.55 to 0.41)	1898.52 (2181.11 to 1657.79)	0.38 (0.43 to 0.33)	−0.86 (−0.9 to −0.82)
	Low-middle SDI	492.44 (585.64 to 410.69)	0.38 (0.45 to 0.31)	1092.41 (1262.47 to 932.60)	0.38 (0.43 to 0.32)	−0.02 (−0.13 to 0.1)
	Low SDI	1047.36 (1179.70 to 879.19)	0.40 (0.49 to 0.31)	416.95 (512.08 to 324.24)	0.35 (0.43 to 0.27)	−0.38 (−0.59 to −0.17)

ASR, age-standardized; SDI, sociodemographic index; AAPC, average annual percentage change; CI, confidence interval; UI, uncertainty interval.

**Figure 2:**
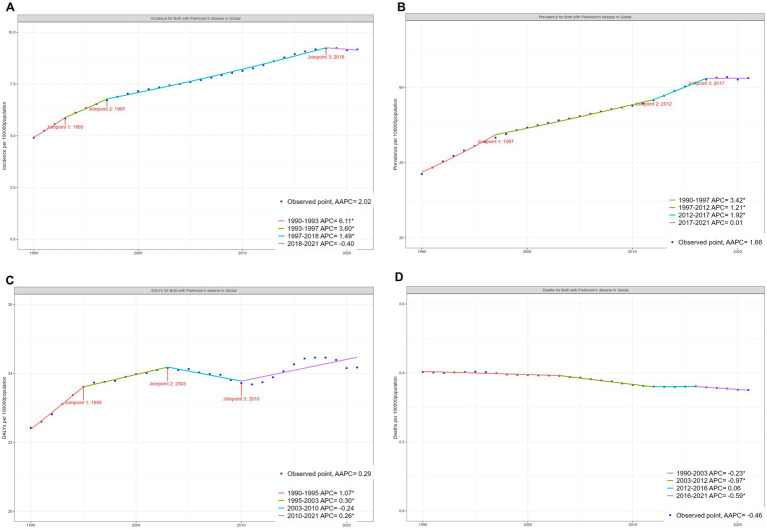
Global trends for age-standardized rates (per 100,000 population) of EOPD from 1990 to 2021. **(A)** Age-standardized incidence rate; **(B)** age-standardized prevalence rate; **(C)** age-standardized DALY rate; **(D)** age-standardized death rate. DALY, disability-adjusted life-year; AAPC, average annual percentage change; APC, annual percentage change.

A longitudinal analysis from 1990 to 2021 revealed differing trends in age-standardized rates. The age-standardized incidence rate (ASIR) experienced steady growth from 1997 to 2018, with an annual percentage change (APC) of 1.49% (95% CI: 1.43–1.55; *p* < 0.05), followed by a modest decline post-2018. Similarly, the age-standardized prevalence rate (ASPR) peaked between 1997 and 2012, with an APC of 1.21% (95% CI: 1.13–1.28), but showed a deceleration in growth after 2017. In contrast, the most significant increase in age-standardized DALY rates occurred earlier, between 1990 and 1995, with an APC of 1.07% (95% CI: 0.75–1.40; *p* < 0.05). Meanwhile, the age-standardized death rate (ASDR) has steadily declined since 1990, with an APC of −0.97% (95% CI: −1.08 to −0.85; *p* < 0.05). This decline may reflect advancements in therapeutic interventions and disease management strategies (Figure 2).

### Regional and national early-onset Parkinson’s disease burden

**Figure 3:**
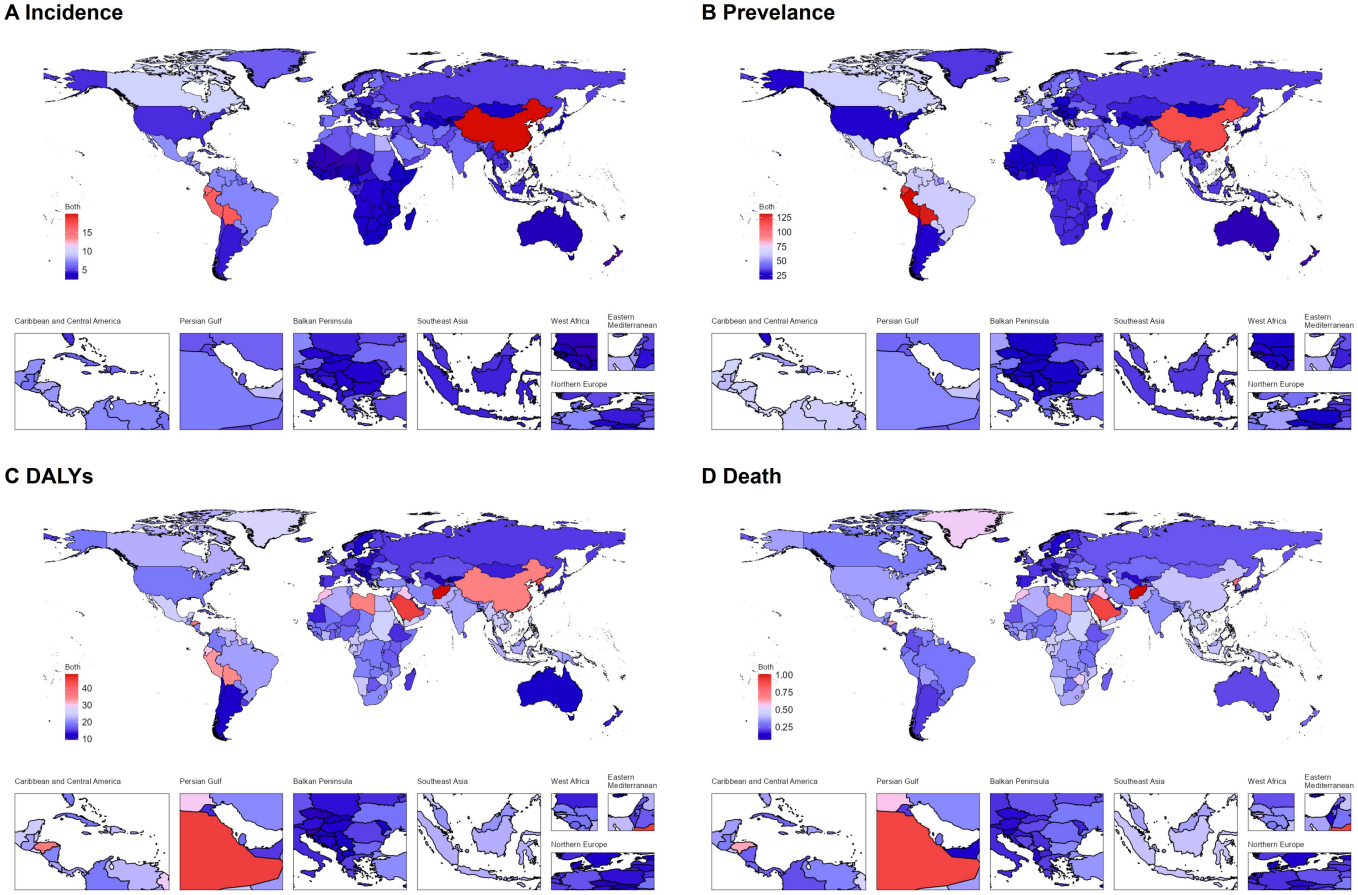
National age-standardized rates (per 100,000 population) of EOPD for both sexes combined in 2021. **(A)** Age-standardized incidence rate; **(B)** age-standardized prevalence rate; **(C)** age-standardized DALY rate; **(D)** age-standardized death rate. DALY, disability-adjusted life-year. The original data was obtained from the GBD studies. There might be problems in the regional division, which was not the critical point for this study.

**Figure 4:**
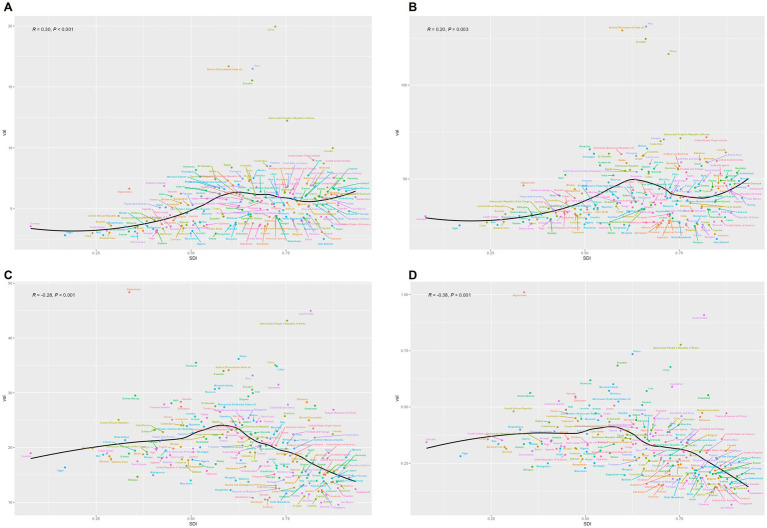
National age-standardized rates (per 100,000 population) of early-onset Parkinson’s disease for both sexes combined in 2021. **(A)** Age-standardized incidence rate; **(B)** age-standardized prevalence rate; **(C)** age-standardized DALY rate; **(D)** age-standardized death rate.

In 2021, regional disparities in EOPD burden were evident based on WHO classifications. East Asia experienced the highest ASIR globally, followed by the Western Pacific Region and Andean Latin America. Conversely, the lowest rates across all metrics, including ASIR, were consistently observed in the subregions of Africa (Western, Eastern, Southern, Northern, and Central Africa). Andean Latin America recorded the highest age-standardized prevalence rate (ASPR), surpassing both East Asia and the Western Pacific Region. Regarding disability burden, East Asia faced the highest age-standardized DALY rate, followed by Andean Latin America and the Western Pacific Region. For mortality, the highest age-standardized death rate (ASDR) was found in the Eastern Mediterranean Region, with North Africa and the Middle East next. Longitudinally (1990–2021), ASIR and ASPR accelerated in 83% of regions, with East Asia exhibiting the most dramatic surge; this contrasted sharply with High-income North America, where ASPR declined significantly (−0.82% APC) despite stable ASIR. Mortality trends revealed 27 regions with rising age-standardized DALY rates, particularly Southern Sub-Saharan Africa, which demonstrated the steepest increase (1.32% APC), while Central Europe achieved the most substantial decline (−1.15% APC). Nationally, China, Bolivia, and Peru had the highest ASIRs. Peru led in ASPR, followed by Bolivia and Ecuador. Afghanistan, Saudi Arabia, and North Korea recorded the highest ASDR and DALY rates. Geopolitically, 179 countries showed rising ASIR/ASPR trends, and 112 had declining mortality metrics, with Italy (−1.07% ASIR APC), Poland (−0.93% ASPR APC), and Kuwait (−2.41% ASDR APC) standing out as national leaders in improvement (Figures 3, 4; Supplementary Tables S1, S2).

### Early-onset Parkinson’s disease burden and SDI

**Figure 5:**
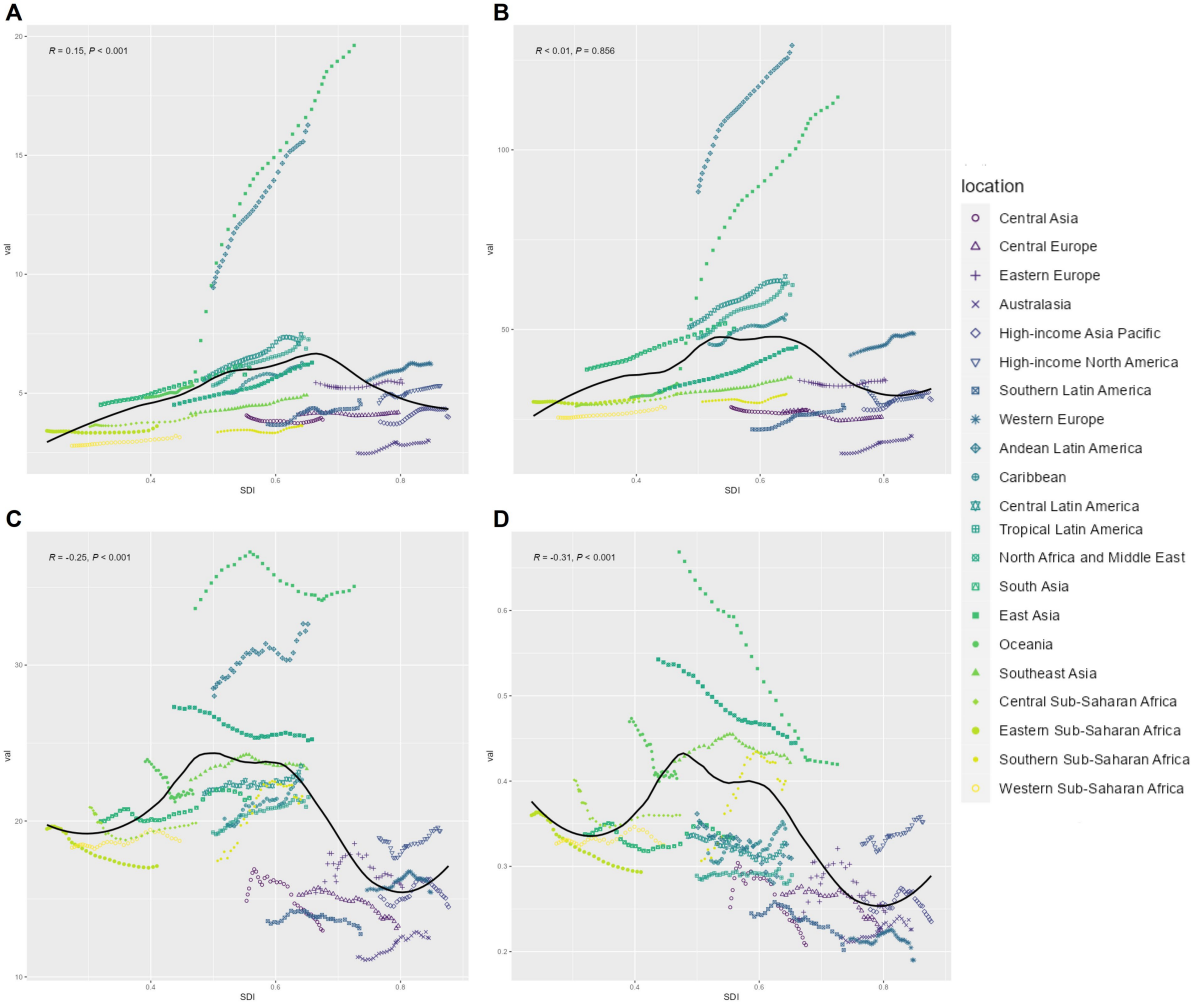
Trends for age-standardized rates (per 100,000 population) of EOPD among 46 regions by SDI for both sexes combined from 1990 to 2021. **(A)** Age-standardized incidence rate; **(B)** age-standardized prevalence rate; **(C)** age-standardized DALY rate; **(D)** age-standardized death rate.

In 2021, the burden of EOPD displayed significant socioeconomic disparities across the spectrum of the Socio-demographic Index (SDI). High-middle SDI countries had the highest age-standardized incidence rate (12.99 per 100,000; 95% UI: 9.01–17.94) and prevalence rate (80.37 per 100,000; 95% UI: 57.80–109.75). In contrast, middle SDI countries experienced the greatest disability burden, with the peak age-standardized disability-adjusted life years (DALY) rate at 27.07 per 100,000 (95% UI: 22.07–33.00) and a mortality rate of 0.38 per 100,000 (95% UI: 0.33–0.43). Geographically, East Asia recorded the highest age-standardized incidence rate (ASIR) of 19.62 and a DALY rate of 35.05, while Andean Latin America showed notable prevalence intensity with an age-standardized prevalence rate (ASPR) of 120.09. Notably, mortality rates were highest in North Africa and the Middle East (age-standardized death rate, ASDR = 0.45), surpassing other regions by 18–23% (see Figure 5).

The relationship between SDI and EOPD showed nonlinear dynamics. Both the ASIR and ASPR followed inverted U-shaped curves along the SDI continuum, peaking in upper-middle SDI territories before declining as economic development continued. This suggests that beyond certain levels of socioeconomic advancement, the occurrence of the disease may decrease. In terms of mortality metrics, age-standardized DALY and death rates initially increased before declining with higher SDI levels, ultimately revealing an overall downward trend. However, considerable regional variability remained; while 62% of high-SDI regions exhibited steady declines, 38% showed fluctuating patterns that were not related to SDI progression. These findings indicate that although global socioeconomic development generally correlates with a reduced burden of EOPD, localized factors—such as healthcare infrastructure and environmental exposures—likely influence the specific trajectories of the disease.

### Decomposition analysis of change in DALYs

**Figure 6:**
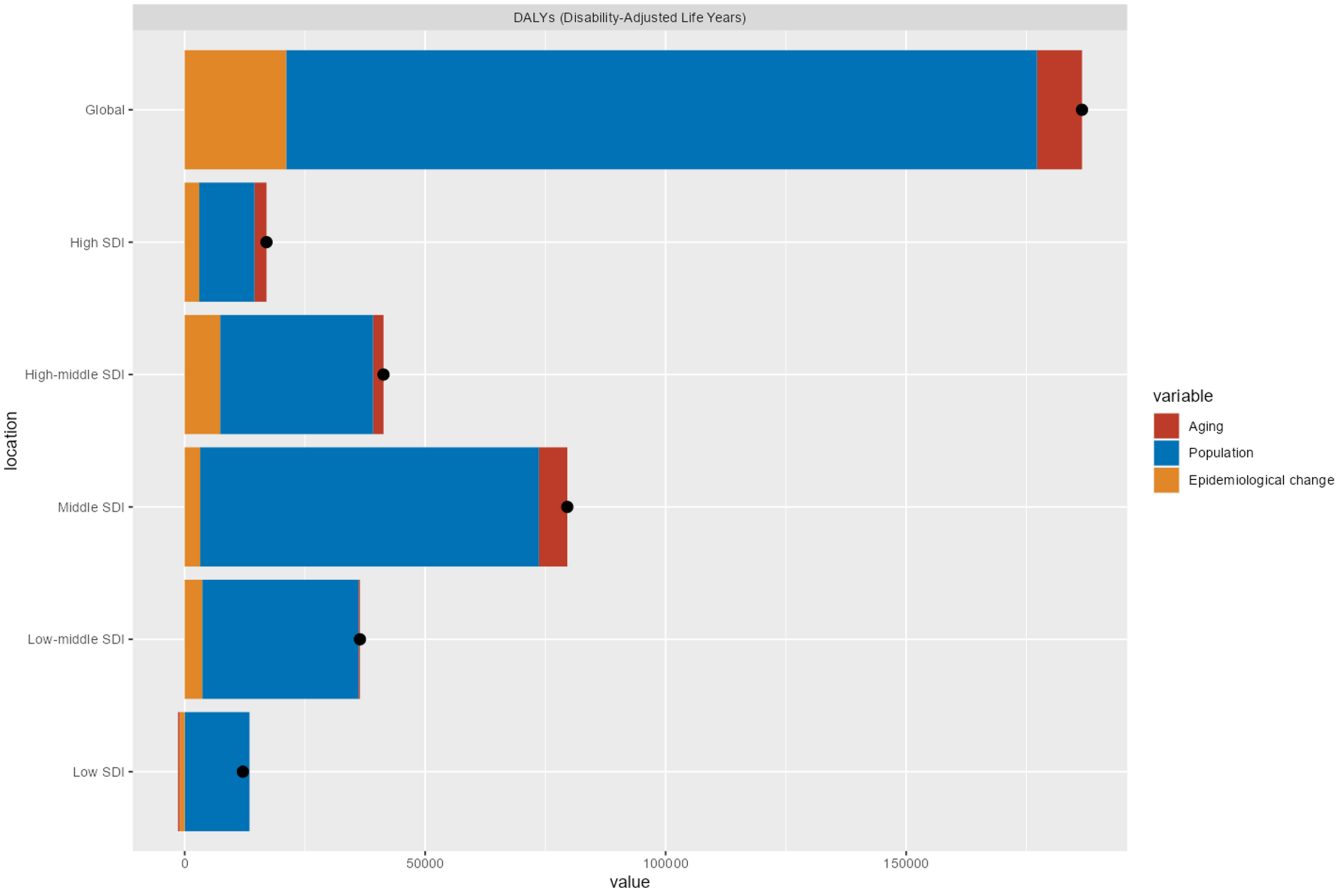
The changes in DALYs associated with EOPD have been analyzed from 1990 to 2021. This analysis explores population-level determinants, such as population growth, aging, and shifts in epidemiology, both globally and by SDI quintile. In the graph, the black dot represents the overall change contributed by all three components. A positive value indicates an increase in EOPD attributed to that component, while a negative value signifies a decrease in EOPD related to the corresponding factor.

**Figure 7:**
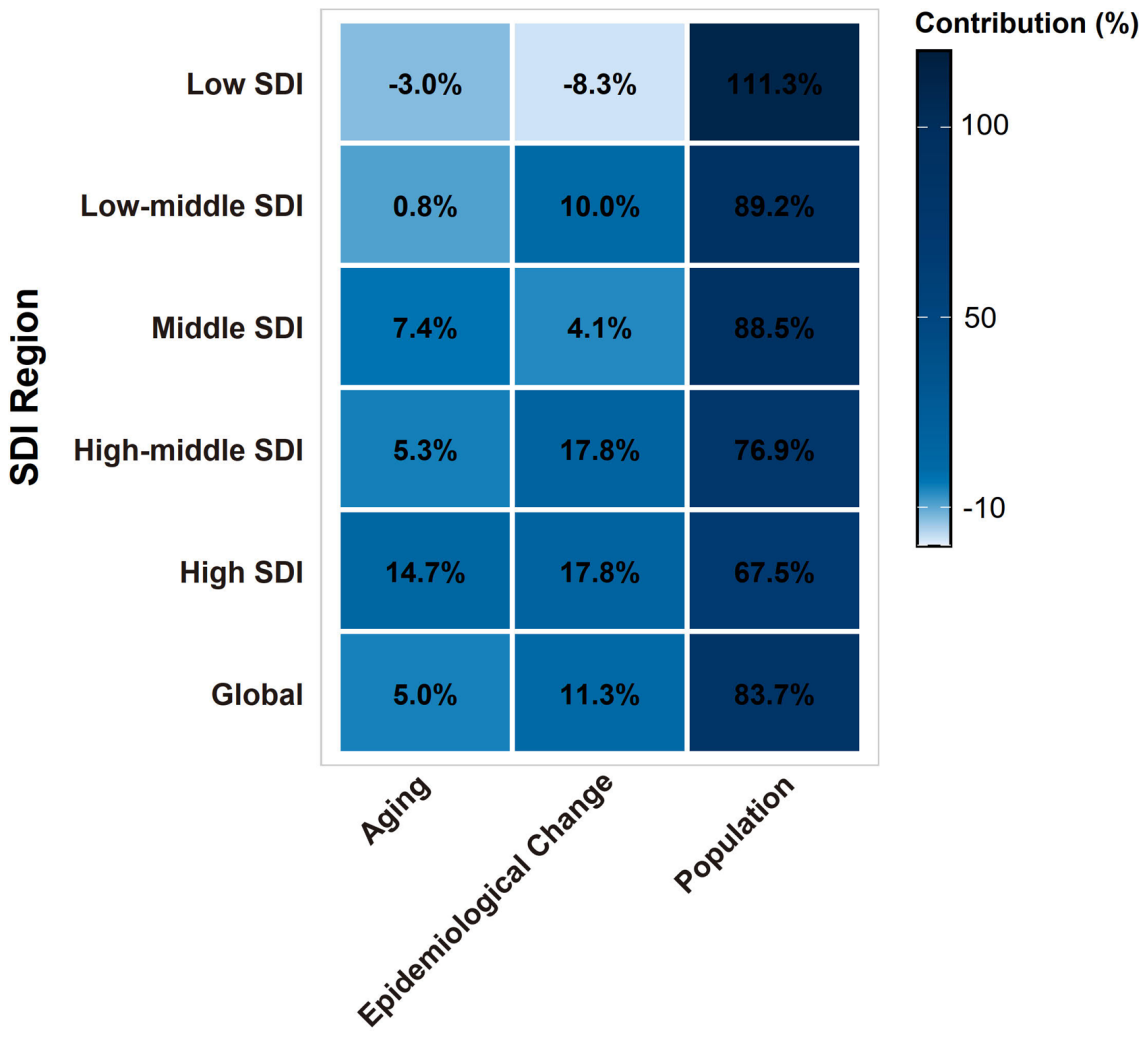
Heatmaps and regional maps for visualizing DALY inequalities in EOPD.

The demographic decomposition analysis of the burden of EOPD reveals distinct drivers across different levels of development (Figures 6, 7; Supplementary Table S3). Between 1990 and 2021, global DALYs related to EOPD increased by 62.3%. Notably, countries with middle SDI quintiles experienced the most significant rise at 89.1%, compared to 38.7% in high SDI and 41.2% in low SDI groups. Population growth emerged as the primary factor contributing to this increase, accounting for 83.7% of the global changes in DALYs, followed by epidemiological shifts at 11.3% and aging effects at 5.0%.

The SDI-stratified analysis identified critical thresholds in trends. The influence of population growth diminished at higher SDI levels, with it accounting for 67.2% in high SDI regions compared to 91.4% in low SDI areas. Epidemiological changes intensified the burden in middle-high and high SDI regions (both at +17.8%), while paradoxically reducing DALYs in low SDI areas by 8.3%.

Aging effects became clinically significant only in regions above the middle SDI threshold, contributing +14.7% in high SDI areas and +7.4% in middle SDI areas, with negligible to negative impacts in lower development levels.

These patterns suggest there are two distinct dynamics in disease burden: lower SDI regions primarily face an escalation in burden driven by population growth, whereas advanced economies contend with challenges intensified by aging and changes in epidemiology. The inverse relationship between SDI and the impact of population growth (*r* = −0.82, *p* < 0.001) highlights the need for development-stage-specific interventions.

### Frontier analysis s on the basis of age-standardized DALYs

**Figure 8:**
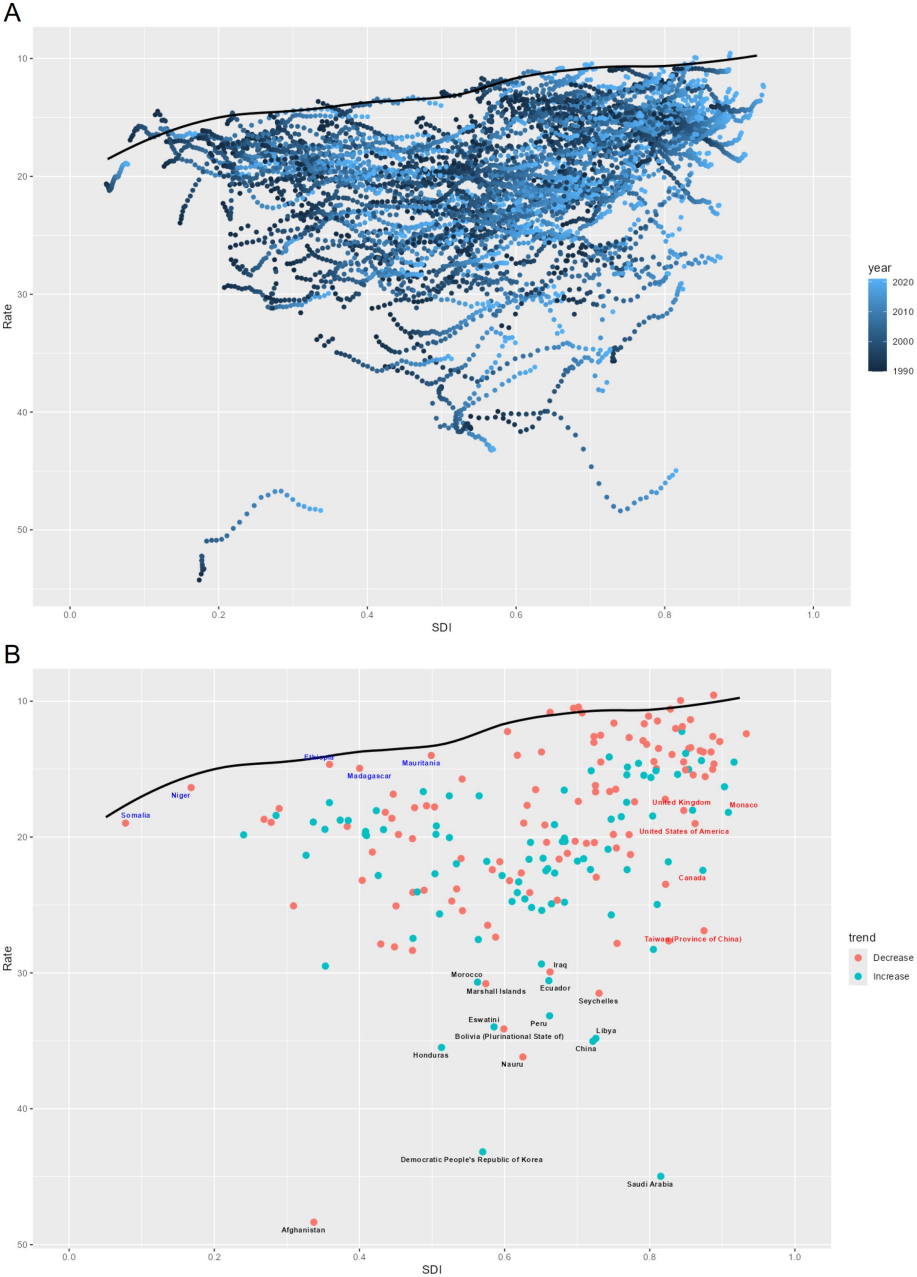
**(A)** Frontier analysis based on the SDI and the age-standardized DALYs rate of EOPD from 1990 to 2021. The color scale represents the years from 1990, shown in dark blue, to 2021, shown in light blue. A solid black line delineates the frontier. **(B)** Frontier analysis based on the SDI and age-standardized DALY rate of EOPD in 2021. The frontier is represented by the solid black line, with countries and territories depicted as dots. An increase in the age-standardized DALYs rate for EOPD from 1990 to 2021 is illustrated with blue dots, while a decrease is shown with red dots. The top 15 countries with the largest effective differences are labeled in black. Examples of frontier countries with a lower SDI (SDI < 0.5) and lower effective differences are labeled in blue, whereas countries and territories with a high SDI (SDI > 0.85) and relatively high effective differences are labeled in red.

To assess the performance of Disability-Adjusted Life Years (DALYs) and to identify practical differences among countries or regions with varying levels of sociodemographic development, we conducted a frontier analysis using age-standardized DALY data and the sociodemographic index from 1990 to 2021 (Figure 8A). The frontier represents the minimum achievable age-standardized DALYs for a given country or territory based on its sociodemographic index. Each dot on the graph indicates the actual age-standardized DALYs reported in these regions. The practical difference, which is the distance from the frontier, measures the gap between observed and theoretically achievable age-standardized DALYs based on the sociodemographic index.

We observed an inverse relationship between the SDI and, but significant variation was noted among countries with middle SDI (Figure 8B), where the most pronounced effective differences appeared.

The top 15 countries or regions with the largest practical differences from the frontier, which ranged from 18.66 to 34.30, included the Marshall Islands, Morocco, Iraq, Ecuador, Seychelles, Peru, Eswatini, Honduras, Bolivia, Libya, China, Nauru, the Democratic People’s Republic of Korea, Afghanistan, and the Kingdom of Saudi Arabia.

In contrast, the 10 countries or regions with the smallest effective differences from the frontier, with practical differences ranging from 0 to 0.59, were San Marino, Armenia, Azerbaijan, Uzbekistan, Slovenia, Somalia, the Czech Republic, Albania, Kyrgyzstan, and Mauritania. This indicates that these areas have achieved the expected burden of DALYs relative to their development status.

Among countries or regions with an SDI below 0.5, Somalia, Niger, Ethiopia, Madagascar, and Mauritania showed age-standardized DALY rates that were close to the frontier. Meanwhile, countries or regions with a higher SDI (>0.85) and relatively improved effective differences included Monaco, the United Kingdom, Canada, Taiwan (Province of China), and the United States of America.

### Cross-country social inequalities analysis

**Figure 9:**
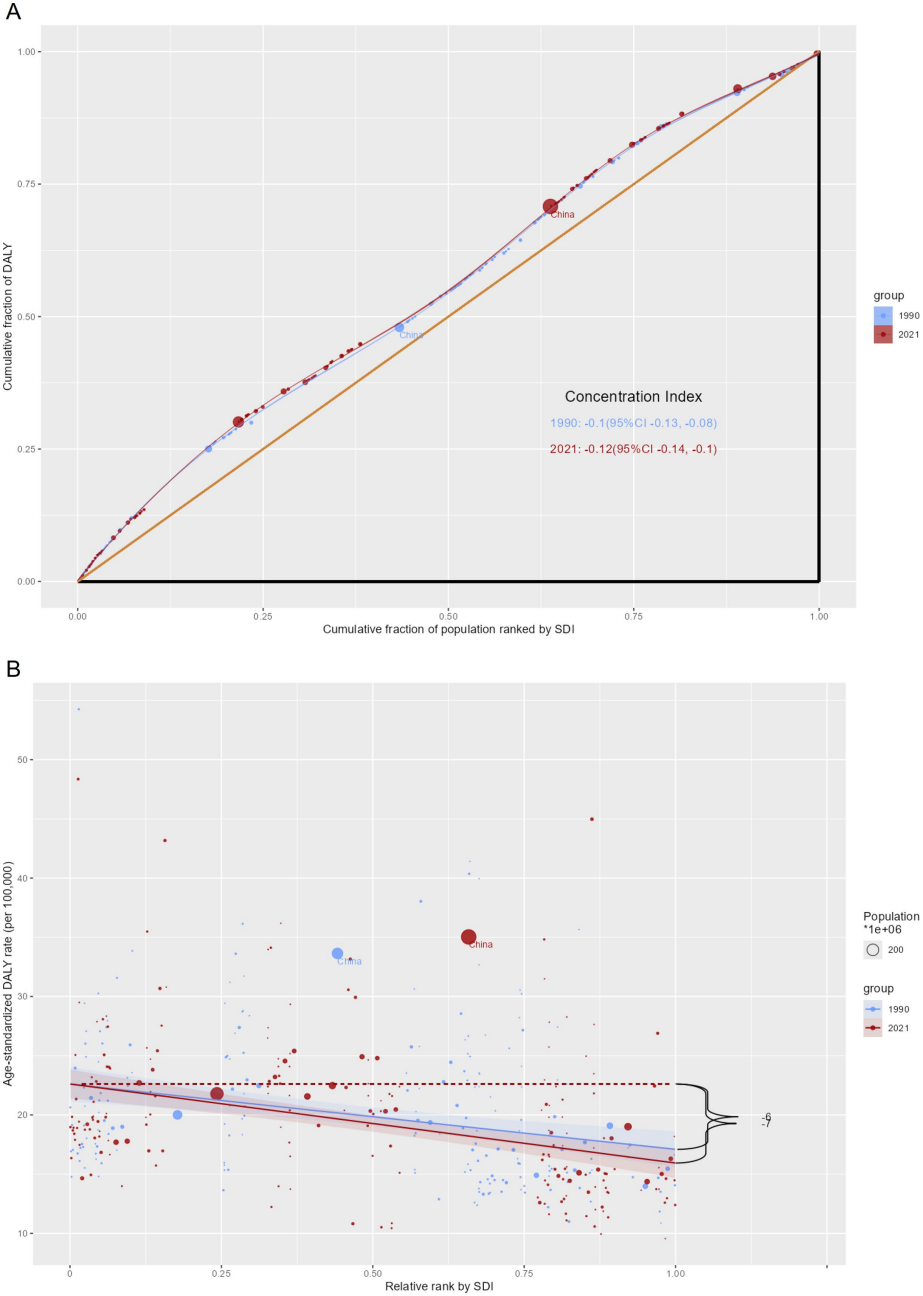
SDI-related cumulative fraction of the population and relative rank curves for the burden of EOPD at the country level for the years 1990 and 2021. **(A)** Cumulative fraction of people ranked by SDI curves. **(B)** Relative rank according to SDI curves.

Significant disparities, both absolute and relative, were evident in the EOPD burden across varying SDI levels (Figure 9). Nations with higher SDI values experienced a disproportionately lower EOPD burden.

The slope index of inequality (SII), quantifying the absolute gap between the highest and lowest SDI countries, showed a slight decrease in magnitude from 7 DALYs per 100,000 population in 1990 to 6 DALYs per 100,000 population in 2021. The concentration index (CI), a measure of relative inequality, worsened from −0.1 in 1990 to −0.12 in 2021, indicating a more pronounced concentration of EOPD burden among lower-SDI countries.

These contrasting trends highlight a complex landscape: while absolute gaps in disease burden have diminished, relative inequalities in burden distribution across socioeconomic strata have persisted or even increased. This underscores the need for targeted interventions to address both absolute and relative disparities in EOPD impact worldwide.

## Discussion

This study offers the most recent and comprehensive analysis of the burden of EOPD globally, regionally, and nationally for individuals aged 40–54 over the past three decades. It confirms findings from a Minnesota cohort study conducted between 2010 and 2015, which reported increasing incidence rates and a predominance of male mortality risk (19). Between 1990 and 2021, the burden of EOPD increased significantly: global incidence rose by 2.7 times and prevalence by 2.4 times, likely due to advancements in diagnostic techniques, such as door-to-door surveys (20) and prolonged disease duration due to increased life expectancy (21). Factors such as changes in lifestyle and dietary habits may also impact health (22, 23).

The age-standardized DALY rates for EOPD have increased by 2.1 times. The ASPR curve has shown fluctuations, beginning with a gradual increase from 1995 to 2003, followed by a decline, and then a resurgence since 2010. This trend is consistent with environmental exposures driven by global industrialization (24). The introduction of DBS for the treatment of movement disorders by Benabid in the late 1980s marked a significant advancement in functional procedures (25–27). However, DBS remains accessible only to a limited subset of eligible patients due to surgical criteria and disparities in healthcare (28). This may help explain the slowdown in the increase of DALYs for EOPD since 1959.

While deaths related to EOPD have shown a decrease in ASDRs, the absolute number of deaths has increased from 1990 to 2021, particularly among males. Although both males and females are experiencing a rise in EOPD cases, males consistently exhibit higher rates, potentially due to genetic factors (29). Global health strategies seem to have positively impacted the reduction of DALYs for both genders, suggesting that advancements in healthcare may be benefiting both males and females (24).

The analysis of trends in EOPD across various geographic regions and countries over the past three decades reveals a complex relationship among socio-demographic, economic, and healthcare factors. The significant disparity in ASPR between Andean Latin America, East Asia, and the Western Pacific Region in comparison to high-income North America suggests that regional influences—such as genetic predisposition, healthcare availability, and environmental factors—play a crucial role. The notable increase in ASPR in East Asia may be attributed to improvements in healthcare infrastructure and advancements in EOPD surveillance methods. In contrast, the rise observed in Africa could be linked to delays in medical progress (30). Furthermore, the substantial increase in ASDR in the Eastern Mediterranean, North Africa, and the Middle East indicates potential weaknesses in their healthcare systems and challenges in accessing timely treatment. Conversely, the decline in mortality rates in regions like West and East Africa may be connected to advancements in healthcare infrastructure and improved socioeconomic conditions that help mitigate risk factors (31).

Socioeconomic status significantly impacts health outcomes, with higher SDI scores generally associated with better health (17, 18, 32). This underscores the need to prioritize socioeconomic development in policy frameworks aimed at addressing the issue of EOPD. However, there is a paradox in regions with a middle SDI, where the age-standardized DALY rates are highest, despite the expectation that socioeconomic advancement would lead to improved health outcomes. This finding was supported by a decomposition analysis which showed that population growth accounted for 89.2% of the increases in DALYs in middle-SDI regions. In contrast, epidemiological shifts and aging were more prominent in high-SDI areas (Figure 6). Demographic dynamics and gaps in healthcare are significant issues in middle-SDI regions, such as China and Bolivia. These regions often face rapid population growth, but their healthcare infrastructure does not expand at the same pace. For instance, China’s incidence of EOPD doubled from 1990 to 2019, largely due to industrialization and a lag in diagnostic capacity (11). Additionally, middle-SDI countries have fewer neurologists per capita compared to their high-SDI counterparts (33).

Middle-SDI regions undergoing industrialization are experiencing increased environmental exposure to neurotoxic agents, such as pesticides and solvents, which are linked to the development of EOPD (34). For instance, rapid industrialization in countries like China has led to a significant increase in pesticide use (35) and heavy metals use (36, 37).

Studies have shown correlations between exposure to industrial chemicals and the risk of Parkinson’s disease (38). At the same time, these regions are undergoing demographic transitions, with aging populations contributing to a rise in disease prevalence. GBD analyses indicate that middle-SDI areas are experiencing accelerated aging trends (12), which further heighten the burden of neurodegenerative disorders. In contrast, high-SDI countries manage these risks more effectively through stricter environmental regulations and early intervention programs, resulting in lower rates of exposure-related diseases in those regions (39).

There are several underlying causes of inequality regarding EOPD. First, genetic variants specific to certain populations significantly influence EOPD risk in middle-SDI regions. For example, particular genetic mutations are more prevalent in certain ethnic groups, while different risk alleles have been identified in other cohorts (40). These genetic predispositions, combined with regional environmental exposures, such as pesticide use, create “gene–environment interaction hotspots” in areas where industrialization outpaces the capacity for genetic screening (41).

Second, middle-SDI regions experience systematic underreporting of EOPD cases due to structural flaws in diagnostic systems. Limited access to neuroimaging facilities and clinician biases—where infectious diseases are prioritized over neurodegenerative disorders in resource-constrained settings—lead to a significant underestimation of the disease’s burden, as seen in global health analyses (42).

Additionally, economic policies significantly contribute to health disparities (17). Despite rising average incomes, middle-SDI countries allocate disproportionately low healthcare budgets to neurology. This creates a vicious cycle of insufficient funding, limited diagnostic capacity, and ongoing neglect of resources. This policy gap exacerbates treatment inequities and highlights the complex interplay between socioeconomic development and healthcare access in shaping disparities related to EOPD.

The escalating prevalence of EOPD over the past 30 years has become a pressing public health issue, particularly impacting males disproportionately, highlighting the necessity for further investigation into the underlying factors contributing to these observed gender disparities. The age-standardized EOPD DALY rates have shown variability, including both declines and periods of stability, yet the projected rise in these rates raises apprehensions regarding the impact of an aging population on future disease prevalence. Despite the worrisome escalation in age-standardized incidence and prevalence rates of EOPD since 1990, the decline in age-standardized mortality rates indicates advancements in the disease. The increasing total DALYs attributed to EOPD highlights the enduring morbidity burden, underscoring the significance of improving patient quality of life post-treatment. These emerging patterns highlight the need for improved health policies aimed at reducing the incidence rates of EOPD and enhancing long-term patient outcomes through effective management and medical interventions.

Our research provides important information on worldwide patterns of EOPD, but it is important to interpret these findings cautiously. Variations in methodology and limitations in the studies may lead to prevalence estimates that differ significantly and underestimate the true burden of EOPD (43).

Addressing this significant health challenge necessitates proactive measures to prevent the disease where possible and enhance the quality of life for individuals impacted by the condition (44). Potential strategies include promoting increased physical activity in early adulthood (26) and minimizing pesticide exposure to prevent the disease (41). Enhancing global access to care and effective treatments, such as levodopa, is imperative. Furthermore, increased research funding to elucidate the underlying causes and develop novel therapies is crucial in effectively addressing this pressing health issue.

## Limitations

This study provides a comprehensive assessment of the global burden of EOPD. However, it presents several limitations that need to be addressed.

Firstly, the GBD framework uses indirect estimation techniques, which pose two significant challenges for accurately assessing the burden of EOPD. First, the inability to distinguish between EOPD subtypes, such as genetic versus idiopathic forms, obscures population-specific risk profiles and progression patterns. This limitation hinders the analysis of varying burdens among groups with different underlying causes (45). In low-and middle-income countries (LMICs), differences in medical record practices and inconsistencies in reporting can result in a systematic underestimation of EOPD prevalence (46, 47). This data sparsity, in turn, compounds the limitations of the SDI as a socioeconomic proxy, which overlooks modifiable risk factors like environmental pollution—factors that may be disproportionately prevalent in regions with poor data infrastructure. Additionally, low-SDI regions often lack detailed data on young-onset cases (48, 49), further biasing GBD’s extrapolation of adult-based estimates.

Finally, the analysis ultimately left out important genetic and biomarker data, such as PRKN/PINK1 mutation profiles and dopamine transporter imaging, which are essential for subclassifying EOPD (50, 51). By excluding this information, the study may overlook key risk factors that are specific to subtypes—for instance, environmental exposures in idiopathic cases compared to genetic predispositions in familial cases. This omission could restrict our understanding of the underlying mechanisms of the disease.

Future research should integrate these factors for a more comprehensive view of EOPD epidemiology.

## Conclusion

This research provides a thorough analysis of the global distribution and 30-year trends of EOPD. It reveals a consistent increase in the global burden of this condition, with significant disparities observed in nations with lower SDI. The findings have important policy implications, highlighting the need for targeted resource allocation to enhance neurological healthcare capacity in low-SDI regions. Additionally, there is a call for the development of stronger surveillance systems in resource-constrained settings to address issues of underdiagnosis and inconsistencies in data collection. The research also emphasizes the importance of cross-regional collaborations to share effective intervention models for integrating EOPD management into primary healthcare. These recommendations stress the necessity for tailored strategies to reduce disparities, as well as the need for further research into region-specific risk factors and affordable diagnostic tools that meet the needs of populations that bear a high burden of the disease.

## Data Availability

The datasets presented in this study can be found in online repositories. The names of the repository/repositories and accession number(s) can be found at: https://ghdx.healthdata.org/gbd-results-tool.

## Footnotes

^1^ https://ghdx.healthdata.org/gbd-results-tool

^2^ http://ghdx.healthdata.org/gbd-results-tool
