# Supplementary material for: Mapping the global burden of early-onset Parkinson’s disease: socioeconomic and regional inequalities from the Global Burden of Disease Study 2021
Source: Front Public Health. 2025 Jul 22;13:1618533. doi: 10.3389/fpubh.2025.1618533 (PMC12321513; doi:10.3389/fpubh.2025.1618533)
Supplement: Supplementary file 1 [file Table_1.docx]

**Supporting Materials**

**Catalogue**

[List of abbreviations 2](#_Toc202446892)

[Table S1. Burden of early-onset Parkinson's disease across 21 regions in 2021 for both genders and gender-specific, with AAPC from 1990 and 2021. 3](#_Toc202446893)

[Table S2. Burden of early-onset Parkinson's disease across 204 countries and territories in 2021 for both genders combined, with AAPC from 1990 and 2021. 11](#_Toc202446894)

[Table S3. Changes in DALYs number according to population-level determinants and causes from 1990 to 2021. 35](#_Toc202446895)

[Table S4. Frontier age-standardized disability-adjusted life-years and corresponding effective difference of EOPD in 2021 in 204 countries or territories. 36](#_Toc202446896)

# List of abbreviations

APC: annual percentage change

AAPC: average annual percentage

ASR: age-standardized rate

ASIR: age-standardized incidence rate

ASPR: age-standardized prevalence rate

ASDR: age-standardized death rate

CI: confidence interval

DALYs: disability-adjusted life years

DBS: deep brain stimulation

EOPD: early-onset Parkinson's disease

GBD: the Global Burden of Diseases

LOPD: late-onset Parkinson's Disease

LMICs: low- and middle-income countries

SDI: socio-demographic index

UI: uncertainty interval

YLL: years of life lost

# Table S1. Burden of early-onset Parkinson's disease across 21 regions in 2021 for both genders and gender-specific, with AAPC from 1990 and 2021.

| **Location** | **Sex** | **Incidence** | | | **Prevalence** | | | **DALYs** | | | **Death** | | |
| --- | --- | --- | --- | --- | --- | --- | --- | --- | --- | --- | --- | --- | --- |
|  |  | **Cases**  **(95% UI)** | **ASIR (per 100, 000) (95% UI)** | **AAPC % (95%CI) 1990–2019** | **Cases**  **(95% UI)** | **ASPR (per 100, 000) (95% UI)** | **AAPC % (95%CI) 1990–2019** | **Cases**  **(95% UI)** | **Age-standardized**  **DALY rate (per**  **100,000) (95% UI)** | **AAPC % (95%CI) 1990–2019** | **Cases (95% UI)** | **ASDR (per 100, 000) (95% UI)** | **AAPC % (95%CI) 1990–2019** |
| Andean Latin America | Both | 1782.62 (2444.00 to 1204.30) | 16.27(22.31 to 10.99) | 1.75  (1.67 to 1.84) | 9583.97 (6599.75 to 12954.34) | 129.09(172.99 to 93.93) | 1.22 (1.17 to 1.27) | 3549.00 (4799.19 to 2642.44) | 32.63(44.07 to 24.32) | 0.49 (0.33 to 0.64) | 33.55 (42.62 to 26.23) | 0.31(0.39 to 0.24) | -0.43 (-1.02 to 0.15) |
|  | Female | 583.95 (844.87 to 372.07) | 10.44(15.11 to 6.66) | 1.54  (1.37 to 1.7) | 4885.29 (6761.43 to 3372.97) | 87.86(121.47 to 60.75) | 1.05 (0.92 to 1.17) | 1251.29 (1797.98 to 844.24) | 22.53(32.33 to 15.21) | 0.31 (0.01 to 0.61) | 12.28 (16.00 to 9.00) | 0.22(0.29 to 0.16) | -0.51 (-1.17 to 0.15) |
|  | Male | 1198.67 (1661.93 to 803.07) | 22.34(30.98 to 14.96) | 1.87  (1.7 to 2.04) | 9162.91 (12353.15 to 6585.95) | 172.06(231.69 to 123.77) | 1.36 (1.23 to 1.48) | 2297.70 (3148.13 to 1664.84) | 43.16(59.07 to 31.31) | 0.56 (0.42 to 0.71) | 21.27 (27.40 to 16.37) | 0.40(0.52 to 0.31) | -0.31 (-0.69 to 0.08) |
| Australasia | Both | 186.79 (282.87 to 106.43) | 3.00(4.57 to 1.69) | 0.62 (0.53 to 0.71) | 1251.46 (1859.05 to 801.04) | 20.25(30.19 to 12.92) | 0.92 (0.82 to 1.01) | 778.20 (916.60 to 673.07) | 12.49(14.75 to 10.78) | 0.33 (0.17 to 0.48) | 14.21 (15.05 to 13.39) | 0.23(0.24 to 0.21) | 0.16 (0.02 to 0.3) |
|  | Female | 76.82  (124.32 to 39.21) | 2.42(3.95 to 1.23) | 0.52 (0.32 to 0.72) | 529.77 (813.64 to 315.89) | 16.84(25.93 to 9.97) | 0.74 (0.53 to 0.94) | 303.96 (368.42 to 257.84) | 9.56(11.62 to 8.09) | 0.16 (0.03 to 0.29) | 5.35 (5.72 to 4.98) | 0.17(0.18 to 0.15) | -0.1 (-0.25 to 0.06) |
|  | Male | 109.97 (165.58 to 62.04) | 3.60(5.45 to 2.01) | 0.77 (0.65 to 0.88) | 721.69 (1073.20 to 450.21) | 23.80(35.51 to 14.81) | 1.05 (0.9 to 1.2) | 474.24 (562.25 to 405.04) | 15.53(18.45 to 13.25) | 0.55 (0.38 to 0.71) | 8.86 (9.55 to 8.18) | 0.29(0.31 to 0.27) | 0.31 (0.1 to 0.51) |
| Caribbean | Both | 546.04 (788.73 to 340.64) | 6.28(9.09 to 3.92) | 0.71 (0.57 to 0.85) | 4748.33 (6522.75 to 3326.78) | 54.16(74.53 to 37.83) | 0.52 (0.44 to 0.61) | 1996.64 (2451.13 to 1629.68) | 22.66(27.85 to 18.45) | 0.44 (0.28 to 0.61) | 30.60 (36.78 to 25.59) | 0.34(0.41 to 0.29) | 0.43 (0.2 to 0.65) |
|  | Female | 217.08 (327.68 to 128.59) | 4.90(7.41 to 2.90) | 0.72 (0.52 to 0.92) | 1904.68 (2700.08 to 1301.53) | 42.73(60.69 to 29.12) | 0.57 (0.5 to 0.64) | 748.79 (958.58 to 581.78) | 16.69(21.38 to 12.95) | 0.32 (0.13 to 0.5) | 10.91 (13.56 to 8.84) | 0.24(0.30 to 0.19) | 0.15 (-0.17 to 0.46) |
|  | Male | 328.95 (471.44 to 208.53) | 7.71(11.06 to 4.89) | 0.69 (0.55 to 0.83) | 2843.65 (3889.13 to 2003.23) | 66.02(90.52 to 46.34) | 0.5 (0.4 to 0.6) | 1247.85 (1535.23 to 1000.75) | 28.84(35.55 to 23.08) | 0.52 (0.33 to 0.71) | 19.69 (24.07 to 16.03) | 0.45(0.55 to 0.37) | 0.57 (0.25 to 0.89) |
| Central Asia | Both | 630.33 (951.92 to 365.01) | 3.89(5.88 to 2.25) | -0.09 (-0.18 to 0) | 4469.68 (6493.75 to 2897.68) | 27.58(40.07 to 17.88) | -0.08 (-0.1 to -0.05) | 2102.62 (2593.37 to 1741.62) | 12.97(16.00 to 10.75) | -0.42 (-0.73 to -0.1) | 33.67 (38.01 to 29.31) | 0.21(0.23 to 0.18) | -0.58 (-0.97 to -0.19) |
|  | Female | 264.72 (403.94 to 149.77) | 3.17(4.84 to 1.79) | -0.13 (-0.16 to -0.09) | 1864.06 (2744.71 to 1159.05) | 22.31(32.86 to 13.86) | -0.06 (-0.1 to -0.03) | 847.75 (1061.15 to 687.57) | 10.13(12.69 to 8.22) | -0.35 (-0.62 to -0.07) | 13.48 (15.75 to 11.51) | 0.16(0.19 to 0.14) | -0.47 (-0.82 to -0.11) |
|  | Male | 365.61 (553.39 to 210.63) | 4.66(7.05 to 2.68) | -0.06 (-0.21 to 0.08) | 2605.62 (3821.33 to 1685.07) | 33.19(48.65 to 21.47) | -0.08 (-0.14 to -0.02) | 1254.88 (1533.61 to 1032.13) | 15.99(19.54 to 13.15) | -0.44 (-0.76 to -0.12) | 20.19 (22.79 to 17.76) | 0.26(0.29 to 0.23) | -0.61 (-1.01 to -0.21) |
| Central Europe | Both | 1089.34 (1525.69 to 719.45) | 4.19(5.87 to 2.76) | 0.06 (0.03 to 0.09) | 6662.66 (9192.80 to 4604.11) | 25.61(35.37 to 17.68) | -0.18 (-0.21 to -0.16) | 3457.23 (4048.93 to 2996.81) | 13.27(15.55 to 11.50) | -0.45 (-0.69 to -0.22) | 58.77 (63.67 to 54.11) | 0.22(0.24 to 0.21) | -0.64 (-1.03 to -0.26) |
|  | Female | 435.13 (613.00 to 280.45) | 3.37(4.75 to 2.17) | -0.03 (-0.05 to -0.02) | 2655.69 (3687.96 to 1814.22) | 20.55(28.56 to 14.03) | -0.33 (-0.39 to -0.28) | 1355.94 (1614.02 to 1159.36) | 10.48(12.48 to 8.95) | -0.49 (-0.58 to -0.39) | 22.88 (25.27 to 20.83) | 0.18(0.19 to 0.16) | -0.57 (-0.9 to -0.24) |
|  | Male | 654.22 (918.80 to 426.39) | 5.00(7.02 to 3.25) | 0.11 (0.08 to 0.14) | 4006.97 (5513.65 to 2797.37) | 30.59(42.13 to 21.35) | -0.1 (-0.16 to -0.04) | 2101.29 (2468.81 to 1809.59) | 16.02(18.83 to 13.79) | -0.46 (-0.71 to -0.21) | 35.89 (38.85 to 32.90) | 0.27(0.30 to 0.25) | -0.64 (-0.89 to -0.4) |
| Central Latin America | Both | 3413.43 (4810.51 to 2220.72) | 7.47(10.53 to 4.86) | 0.87 (0.67 to 1.07) | 29594.57 (39855.78 to 21299.28) | 64.74(87.21 to 46.59) | 0.77 (0.67 to 0.87) | 10754.84 (13182.00 to 8866.66) | 23.52(28.84 to 19.39) | 0.23 (0.15 to 0.3) | 149.63 (168.07 to 132.33) | 0.33(0.37 to 0.29) | -0.18 (-0.34 to -0.02) |
|  | Female | 1377.05 (1987.12 to 857.29) | 5.75(8.31 to 3.58) | 0.95 (0.83 to 1.07) | 12128.49 (16776.73 to 8552.87) | 50.62(70.04 to 35.68) | 0.8 (0.75 to 0.85) | 4317.51 (5400.53 to 3489.45) | 18.02(22.54 to 14.56) | 0.19 (0.09 to 0.3) | 58.74 (67.29 to 50.24) | 0.24(0.28 to 0.21) | -0.26 (-0.32 to -0.19) |
|  | Male | 2036.38 (2837.62 to 1340.77) | 9.37(13.05 to 6.17) | 0.87 (0.65 to 1.09) | 17466.08 (23290.89 to 12532.28) | 80.31(107.10 to 57.62) | 0.78 (0.63 to 0.94) | 6437.33 (8001.48 to 5272.86) | 29.60(36.79 to 24.24) | 0.3 (0.18 to 0.41) | 90.90 (104.54 to 78.82) | 0.42(0.48 to 0.36) | -0.11 (-0.26 to 0.04) |
| Central Sub-Saharan Africa | Both | 595.21 (900.69 to 343.13) | 4.07(6.13 to 2.35) | 0.41 (0.34 to 0.47) | 4720.90 (6777.60 to 3099.44) | 32.36(46.34 to 21.28) | 0.4 (0.38 to 0.43) | 2868.52 (3650.11 to 2124.21) | 19.87(25.28 to 14.72) | -0.17 (-0.22 to -0.13) | 51.45 (69.20 to 36.77) | 0.36(0.48 to 0.26) | -0.37 (-0.48 to -0.27) |
|  | Female | 252.93 (381.58 to 139.61) | 3.46(5.20 to 1.92) | 0.45 (0.27 to 0.62) | 1896.18 (2793.07 to 1201.17) | 26.04(38.25 to 16.55) | 0.42 (0.37 to 0.46) | 1126.67 (1535.20 to 783.31) | 15.69(21.41 to 10.91) | -0.03 (-0.1 to 0.03) | 20.12 (29.20 to 13.08) | 0.28(0.41 to 0.18) | -0.18 (-0.27 to -0.1) |
|  | Male | 342.28 (526.19 to 196.54) | 4.68(7.17 to 2.69) | 0.29 (0.1 to 0.48) | 2824.73 (4036.85 to 1877.71) | 38.68(55.13 to 25.76) | 0.32 (0.28 to 0.37) | 1741.85 (2271.01 to 1268.36) | 24.05(31.34 to 17.53) | -0.33 (-0.42 to -0.24) | 31.33 (42.66 to 22.24) | 0.44(0.59 to 0.31) | -0.55 (-0.68 to -0.42) |
| East Asia | Both | 72204.00 (98226.70 to 51138.32) | 19.62(26.82 to 13.79) | 3.88 (3.71 to 4.05) | 437067.39 (590472.43 to 315101.93) | 114.70(155.32 to 82.62) | 3.48 (3.37 to 3.59) | 131144.12 (170442.75 to 101059.06) | 35.05(45.47 to 27.11) | 0.11 (0.05 to 0.17) | 1566.94 (1981.13 to 1254.07) | 0.42(0.53 to 0.34) | -1.48 (-1.56 to -1.39) |
|  | Female | 27449.21 (37390.54 to 19106.23) | 15.12(20.72 to 10.42) | 3.28 (2.9 to 3.66) | 167116.64 (227612.26 to 117996.24) | 88.96(121.60 to 62.73) | 2.99 (2.72 to 3.26) | 48690.75 (64586.90 to 36366.98) | 26.17(34.84 to 19.55) | -0.42 (-0.5 to -0.35) | 569.85 (810.88 to 426.43) | 0.30(0.43 to 0.23) | -2.1 (-2.19 to -2) |
|  | Male | 44754.79 (61150.92 to 31942.46) | 23.98(32.90 to 17.00) | 4.39 (4.21 to 4.57) | 269950.75 (363535.03 to 195951.28) | 139.74(188.56 to 101.35) | 3.89 (3.78 to 4.01) | 82453.37 (108637.04 to 61014.82) | 43.66(57.35 to 32.45) | 0.49 (0.43 to 0.55) | 997.09 (1324.27 to 747.91) | 0.53(0.70 to 0.40) | -1.09 (-1.19 to -0.99) |
| Eastern Europe | Both | 2357.40 (3350.22 to 1491.55) | 5.42(7.71 to 3.43) | 0.01 (-0.08 to 0.1) | 15522.38 (21674.20 to 10608.88) | 35.70(49.88 to 24.39) | 0.01 (-0.04 to 0.06) | 6849.14 (8311.81 to 5701.49) | 15.74(19.11 to 13.10) | 0.1 (-0.33 to 0.53) | 107.30 (118.04 to 96.59) | 0.25(0.27 to 0.22) | 0.2 (-0.7 to 1.11) |
|  | Female | 1033.31 (1482.43 to 650.61) | 4.52(6.49 to 2.84) | 0 (-0.19 to 0.19) | 6668.68 (9380.27 to 4469.37) | 29.14(41.03 to 19.51) | -0.01 (-0.06 to 0.04) | 2968.77 (3670.17 to 2447.86) | 12.96(16.03 to 10.68) | 0.16 (-0.42 to 0.74) | 46.54 (53.81 to 40.56) | 0.20(0.23 to 0.18) | 0.18 (-1.06 to 1.45) |
|  | Male | 1324.09 (1884.60 to 839.76) | 6.43(9.15 to 4.08) | 0.09 (0.04 to 0.14) | 8853.70 (12334.45 to 6066.14) | 42.97(59.87 to 29.44) | 0.03 (-0.02 to 0.07) | 3880.37 (4763.14 to 3170.65) | 18.83(23.12 to 15.39) | 0.05 (-0.28 to 0.37) | 60.76 (69.90 to 52.66) | 0.29(0.34 to 0.26) | 0.17 (-0.71 to 1.05) |
| Eastern Sub-Saharan Africa | Both | 1565.00 (2319.80 to 911.23) | 3.62(5.33 to 2.11) | 0.18 (0.14 to 0.23) | 13579.50 (19063.66 to 9281.32) | 31.53(44.10 to 21.61) | 0.19 (0.15 to 0.22) | 7260.23 (8990.33 to 5645.71) | 17.11(21.16 to 13.32) | -0.43 (-0.48 to -0.38) | 122.30 (154.65 to 91.51) | 0.29(0.37 to 0.22) | -0.68 (-0.75 to -0.61) |
|  | Female | 635.42 (944.16 to 362.76) | 2.91(4.29 to 1.67) | 0.12 (0.03 to 0.22) | 5143.67 (7406.04 to 3413.72) | 23.71(33.92 to 15.83) | 0.11 (0.02 to 0.2) | 2706.73 (3533.27 to 1962.85) | 12.72(16.59 to 9.22) | -0.41 (-0.47 to -0.35) | 45.54 (61.53 to 31.32) | 0.22(0.29 to 0.15) | -0.63 (-0.71 to -0.55) |
|  | Male | 929.58 (1392.01 to 540.22) | 4.35(6.47 to 2.54) | 0.22 (0.18 to 0.25) | 8435.83 (11744.42 to 5820.01) | 39.54(54.93 to 27.36) | 0.23 (0.19 to 0.26) | 4553.50 (5682.73 to 3570.38) | 21.61(26.91 to 16.99) | -0.45 (-0.49 to -0.4) | 76.77 (99.15 to 58.27) | 0.37(0.48 to 0.28) | -0.71 (-0.78 to -0.64) |
| High-income Asia Pacific | Both | 1775.92 (2549.14 to 1078.09) | 4.00(5.77 to 2.42) | 0.15 (0.07 to 0.23) | 13535.82 (19063.16 to 9274.40) | 30.44(43.07 to 20.78) | 0.29 (0.19 to 0.39) | 6523.22 (7795.37 to 5564.45) | 14.49(17.37 to 12.33) | -0.12 (-0.29 to 0.05) | 107.40 (114.20 to 101.65) | 0.24(0.25 to 0.22) | -0.26 (-0.61 to 0.09) |
|  | Female | 746.85 (1097.76 to 434.66) | 3.42(5.04 to 1.98) | -0.02 (-0.16 to 0.13) | 5960.04 (8414.50 to 3989.97) | 27.24(38.61 to 18.18) | 0.08 (-0.1 to 0.26) | 2690.82 (3245.87 to 2247.77) | 12.14(14.69 to 10.11) | 0 (-0.12 to 0.12) | 42.63 (46.64 to 38.71) | 0.19(0.21 to 0.17) | -0.08 (-0.3 to 0.14) |
|  | Male | 1029.07 (1468.34 to 641.38) | 4.56(6.54 to 2.83) | 0.27 (0.21 to 0.32) | 7575.78 (10568.70 to 5239.90) | 33.55(47.04 to 23.10) | 0.46 (0.37 to 0.54) | 3832.40 (4593.08 to 3257.08) | 16.78(20.17 to 14.23) | -0.2 (-0.39 to 0) | 64.77 (69.74 to 60.72) | 0.28(0.30 to 0.26) | -0.42 (-0.85 to 0.01) |
| High-income North America | Both | 3867.93 (4729.43 to 3087.46) | 5.32(6.54 to 4.22) | 0.47 (0.43 to 0.51) | 22914.90 (27554.46 to 18889.96) | 31.45(37.88 to 25.87) | -0.36 (-0.42 to -0.3) | 14109.12 (15585.23 to 12861.29) | 19.37(21.40 to 17.64) | 0.08 (-0.17 to 0.33) | 258.44 (265.40 to 251.39) | 0.35(0.36 to 0.34) | 0.3 (0.04 to 0.56) |
|  | Female | 1343.35 (1682.67 to 1033.05) | 3.64(4.59 to 2.78) | 0.21 (0.17 to 0.24) | 8155.71 (9963.07 to 6576.70) | 22.12(27.06 to 17.79) | -0.58 (-0.63 to -0.53) | 5094.94 (5702.25 to 4615.86) | 13.81(15.46 to 12.51) | -0.15 (-0.25 to -0.04) | 93.71 (96.73 to 90.60) | 0.25(0.26 to 0.24) | 0.07 (-0.21 to 0.35) |
|  | Male | 2524.59 (3052.15 to 2043.80) | 7.04(8.55 to 5.67) | 0.61 (0.57 to 0.66) | 14759.19 (17612.19 to 12277.76) | 41.05(49.05 to 34.08) | -0.28 (-0.31 to -0.25) | 9014.18 (10009.37 to 8180.88) | 25.08(27.86 to 22.75) | 0.18 (-0.01 to 0.37) | 164.73 (170.41 to 158.58) | 0.46(0.47 to 0.44) | 0.41 (0.28 to 0.54) |
| North Africa and Middle East | Both | 6638.96 (9289.19 to 4459.06) | 6.28(8.77 to 4.23) | 1.06 (1.03 to 1.1) | 47365.57 (65365.57 to 33159.88) | 45.10(62.06 to 31.68) | 1.03 (0.99 to 1.07) | 26332.11 (31459.61 to 21896.62) | 25.24(30.11 to 21.00) | -0.25 (-0.3 to -0.2) | 459.96 (544.07 to 373.31) | 0.44(0.53 to 0.36) | -0.65 (-0.7 to -0.6) |
|  | Female | 3014.37(4184.43 to 2053.19) | 6.05(8.39 to 4.13) | 1.05 (0.98 to 1.13) | 21706.96(29594.34 to 15197.55) | 43.98(59.72 to 30.89) | 0.97 (0.94 to 1) | 11506.30(14173.86 to 8816.94) | 23.44(28.83 to 17.96) | -0.37 (-0.42 to -0.31) | 197.93(246.42 to 137.27) | 0.41(0.51 to 0.28) | -0.78 (-0.85 to -0.71) |
|  | Male | 3624.60(5133.48 to 2353.56) | 6.49(9.16 to 4.23) | 1.08 (1.05 to 1.12) | 25658.61(35977.62 to 17930.53) | 46.10(64.48 to 32.30) | 1.08 (1.03 to 1.12) | 14825.81(17833.46 to 12333.22) | 26.84(32.25 to 22.36) | -0.17 (-0.22 to -0.12) | 262.03(309.88 to 220.23) | 0.48(0.57 to 0.40) | -0.56 (-0.62 to -0.5) |
| Oceania | Both | 102.01 (146.85 to 65.55) | 5.33(7.64 to 3.44) | 0.38 (0.3 to 0.46) | 661.53 (937.81 to 444.39) | 34.62(48.95 to 23.29) | 0.39 (0.36 to 0.42) | 409.65 (564.92 to 305.02) | 21.73(29.99 to 16.17) | -0.3 (-0.44 to -0.15) | 7.53 (11.18 to 5.31) | 0.40(0.60 to 0.28) | -0.49 (-0.69 to -0.29) |
|  | Female | 48.73(70.96 to 30.68) | 5.27(7.64 to 3.34) | 0.43 (0.31 to 0.56) | 279.49(417.14 to 177.36) | 30.29(45.05 to 19.31) | 0.41 (0.32 to 0.49) | 192.99(279.56 to 130.24) | 21.40(31.05 to 14.41) | -0.24 (-0.29 to -0.19) | 3.73(5.87 to 2.29) | 0.42(0.66 to 0.26) | -0.39 (-0.61 to -0.18) |
|  | Male | 53.28(79.00 to 33.31) | 5.39(7.98 to 3.38) | 0.32 (0.25 to 0.39) | 382.04(540.64 to 257.22) | 38.76(54.79 to 26.13) | 0.38 (0.33 to 0.43) | 216.66(303.55 to 158.90) | 22.12(31.03 to 16.22) | -0.34 (-0.48 to -0.19) | 3.80(5.74 to 2.66) | 0.39(0.59 to 0.27) | -0.58 (-0.79 to -0.37) |
| South Asia | Both | 17888.05 (25997.43 to 11273.56) | 6.04(8.78 to 3.81) | 0.87 (0.77 to 0.98) | 148119.95 (204764.30 to 104201.10) | 50.18(69.32 to 35.34) | 0.79 (0.68 to 0.9) | 63270.96 (77568.56 to 50707.89) | 21.48(26.32 to 17.22) | 0.23 (0.09 to 0.38) | 977.28 (1173.39 to 798.45) | 0.33(0.40 to 0.27) | -0.04 (-0.27 to 0.18) |
|  | Female | 9071.75(13084.19 to 5768.08) | 6.20(8.94 to 3.95) | 0.8 (0.63 to 0.96) | 69646.21(96466.83 to 48964.32) | 47.75(66.08 to 33.61) | 0.7 (0.56 to 0.83) | 25415.15(32673.28 to 19659.18) | 17.46(22.43 to 13.51) | 0.04 (-0.17 to 0.25) | 358.99(450.89 to 263.07) | 0.25(0.31 to 0.18) | -0.36 (-0.68 to -0.04) |
|  | Male | 8816.30(12979.46 to 5385.41) | 5.89(8.66 to 3.60) | 0.94 (0.65 to 1.22) | 78473.74(107472.60 to 55260.68) | 52.55(71.94 to 37.06) | 0.87 (0.8 to 0.94) | 37855.82(47429.14 to 29174.00) | 25.42(31.84 to 19.59) | 0.4 (0.29 to 0.51) | 618.29(801.09 to 469.08) | 0.42(0.54 to 0.32) | 0.2 (-0.01 to 0.41) |
| Southeast Asia | Both | 6611.69 (9471.14 to 4200.26) | 4.92(7.05 to 3.12) | 0.54 (0.49 to 0.6) | 49237.50 (68846.66 to 34250.81) | 36.61(51.21 to 25.47) | 0.45 (0.41 to 0.49) | 31386.04 (37390.85 to 26593.04) | 23.34(27.81 to 19.78) | 0.13 (0.07 to 0.19) | 567.27 (669.30 to 486.72) | 0.42(0.50 to 0.36) | 0.02 (-0.05 to 0.1) |
|  | Female | 2712.10(3938.20 to 1659.26) | 4.00(5.82 to 2.44) | 0.29 (0.25 to 0.33) | 19555.90(27890.68 to 13400.49) | 28.85(41.16 to 19.76) | 0.3 (0.24 to 0.35) | 11415.48(14559.79 to 9249.01) | 16.84(21.47 to 13.64) | -0.18 (-0.21 to -0.14) | 200.82(264.44 to 157.76) | 0.30(0.39 to 0.23) | -0.35 (-0.44 to -0.26) |
|  | Male | 3899.58(5588.85 to 2489.51) | 5.84(8.38 to 3.73) | 0.73 (0.61 to 0.85) | 29681.60(40758.11 to 20855.85) | 44.49(61.09 to 31.26) | 0.53 (0.49 to 0.57) | 19970.56(23958.30 to 16411.46) | 29.94(35.91 to 24.60) | 0.29 (0.22 to 0.36) | 366.46(441.20 to 300.18) | 0.55(0.66 to 0.45) | 0.19 (0.14 to 0.24) |
| Southern Latin America | Both | 578.51 (840.08 to 335.85) | 4.53(6.59 to 2.63) | 0.67 (0.42 to 0.93) | 3591.47 (5173.78 to 2297.37) | 28.15(40.58 to 18.00) | 0.82 (0.68 to 0.97) | 1627.72 (2005.40 to 1345.63) | 12.75(15.71 to 10.54) | -0.16 (-0.3 to -0.02) | 25.81 (27.16 to 24.39) | 0.20(0.21 to 0.19) | -0.53 (-0.72 to -0.34) |
|  | Female | 202.00(314.43 to 110.05) | 3.08(4.79 to 1.68) | 0.67 (0.54 to 0.8) | 1310.77(1968.87 to 807.87) | 20.00(30.06 to 12.32) | 0.8 (0.67 to 0.92) | 608.64(757.22 to 491.42) | 9.27(11.54 to 7.49) | -0.15 (-0.28 to -0.02) | 9.69(10.38 to 9.03) | 0.15(0.16 to 0.14) | -0.53 (-0.72 to -0.35) |
|  | Male | 376.52(553.01 to 215.17) | 6.08(8.92 to 3.47) | 0.66 (0.35 to 0.98) | 2280.70(3338.87 to 1466.40) | 36.79(53.87 to 23.65) | 0.85 (0.61 to 1.09) | 1019.08(1296.22 to 827.73) | 16.43(20.90 to 13.35) | -0.15 (-0.38 to 0.07) | 16.12(17.31 to 15.00) | 0.26(0.28 to 0.24) | -0.51 (-0.77 to -0.26) |
| Southern Sub-Saharan Africa | Both | 439.23 (652.78 to 258.86) | 3.63(5.38 to 2.14) | 0.24 (0.18 to 0.3) | 3864.07 (5399.88 to 2716.36) | 32.00(44.62 to 22.51) | 0.22 (0.19 to 0.25) | 2589.13 (3027.17 to 2187.12) | 21.59(25.21 to 18.25) | 0.65 (0.31 to 1) | 47.55 (55.15 to 40.22) | 0.40(0.46 to 0.34) | 0.8 (0.36 to 1.25) |
|  | Female | 175.06(266.03 to 96.76) | 2.79(4.24 to 1.55) | 0.29 (0.15 to 0.43) | 1476.34(2099.16 to 992.43) | 23.61(33.52 to 15.90) | 0.28 (0.19 to 0.36) | 938.25(1191.60 to 730.05) | 15.04(19.08 to 11.72) | 0.58 (0.22 to 0.93) | 16.93(22.13 to 12.76) | 0.27(0.36 to 0.21) | 0.78 (0.46 to 1.11) |
|  | Male | 264.17(393.16 to 157.78) | 4.52(6.71 to 2.70) | 0.17 (0.09 to 0.25) | 2387.74(3304.10 to 1679.72) | 41.03(56.60 to 28.93) | 0.18 (0.14 to 0.21) | 1650.88(1956.83 to 1375.46) | 28.71(34.02 to 23.95) | 0.62 (0.34 to 0.9) | 30.61(36.50 to 25.26) | 0.54(0.64 to 0.45) | 0.76 (0.4 to 1.13) |
| Tropical Latin America | Both | 3236.06 (4614.55 to 2057.00) | 7.26(10.36 to 4.61) | 1.03 (0.91 to 1.15) | 27763.70 (37511.81 to 19677.12) | 62.36(84.24 to 44.22) | 0.87 (0.64 to 1.09) | 9581.68 (11757.04 to 7879.24) | 21.53(26.42 to 17.71) | 0.38 (0.32 to 0.44) | 128.83 (133.31 to 124.26) | 0.29(0.30 to 0.28) | 0.02 (-0.2 to 0.24) |
|  | Female | 1321.80(1903.21 to 811.93) | 5.74(8.27 to 3.52) | 1.23 (1.12 to 1.34) | 11298.73(15437.40 to 7907.97) | 49.05(67.00 to 34.34) | 0.94 (0.81 to 1.08) | 3799.86(4771.94 to 3044.23) | 16.50(20.72 to 13.22) | 0.44 (0.37 to 0.51) | 49.80(52.07 to 47.38) | 0.22(0.23 to 0.21) | 0.15 (-0.18 to 0.48) |
|  | Male | 1914.26(2731.53 to 1235.61) | 8.90(12.70 to 5.74) | 0.85 (0.36 to 1.35) | 16464.97(22315.31 to 11776.78) | 76.64(103.81 to 54.86) | 0.81 (0.51 to 1.11) | 5781.82(7178.09 to 4744.62) | 26.94(33.43 to 22.12) | 0.3 (0.22 to 0.38) | 79.03(82.79 to 75.25) | 0.37(0.39 to 0.35) | -0.01 (-0.23 to 0.21) |
| Western Europe | Both | 5971.81 (8411.86 to 3851.23) | 6.22(8.84 to 3.97) | 0.39 (0.29 to 0.5) | 46934.46 (64367.02 to 32823.04) | 48.80(67.23 to 34.05) | 0.43 (0.35 to 0.51) | 14982.78 (18960.17 to 11984.30) | 15.42(19.60 to 12.29) | -0.04 (-0.14 to 0.05) | 188.41 (193.39 to 183.21) | 0.19(0.20 to 0.18) | -0.43 (-0.62 to -0.24) |
|  | Female | 2432.00(3459.23 to 1523.69) | 5.04(7.22 to 3.12) | 0.32 (0.27 to 0.37) | 17238.25(24040.57 to 11641.36) | 35.46(49.71 to 23.85) | 0.44 (0.39 to 0.49) | 5808.57(7374.93 to 4624.24) | 11.85(15.12 to 9.39) | -0.03 (-0.16 to 0.11) | 76.04(78.50 to 73.29) | 0.15(0.16 to 0.15) | -0.36 (-0.59 to -0.13) |
|  | Male | 3539.81(5006.95 to 2296.71) | 7.41(10.58 to 4.76) | 0.46 (0.35 to 0.58) | 29696.22(40327.26 to 20931.80) | 62.22(84.85 to 43.71) | 0.39 (0.26 to 0.53) | 9174.21(11797.33 to 7227.65) | 19.02(24.56 to 14.94) | -0.07 (-0.18 to 0.05) | 112.37(115.91 to 108.73) | 0.23(0.24 to 0.22) | -0.47 (-0.64 to -0.29) |
| Western Sub-Saharan Africa | Both | 1571.51 (2379.61 to 887.41) | 3.15(4.74 to 1.78) | 0.43 (0.39 to 0.47) | 14099.24 (19924.97 to 9518.83) | 28.22(39.81 to 19.07) | 0.35 (0.31 to 0.39) | 9687.25 (13462.65 to 7177.95) | 18.73(25.87 to 13.93) | 0.07 (-0.03 to 0.17) | 168.32 (245.56 to 112.88) | 0.33(0.47 to 0.22) | -0.03 (-0.17 to 0.11) |
|  | Female | 692.70(1046.89 to 387.23) | 2.68(4.04 to 1.51) | 0.53 (0.41 to 0.65) | 5343.57(7721.12 to 3427.66) | 20.71(29.85 to 13.32) | 0.43 (0.37 to 0.49) | 2190.84(2809.21 to 1571.73) | 8.59(11.00 to 6.17) | -0.03 (-0.1 to 0.04) | 32.62(41.64 to 20.66) | 0.13(0.17 to 0.08) | -0.28 (-0.37 to -0.18) |
|  | Male | 878.81(1363.90 to 473.82) | 3.65(5.64 to 1.97) | 0.43 (0.4 to 0.46) | 8755.68(12284.85 to 6009.03) | 36.33(50.89 to 24.95) | 0.47 (0.45 to 0.49) | 7496.41(10928.30 to 5343.72) | 29.62(42.99 to 21.20) | 0.34 (0.2 to 0.48) | 135.70(208.95 to 88.80) | 0.54(0.82 to 0.35) | 0.31 (0.16 to 0.46) |

# Table S2. Burden of early-onset Parkinson's disease across 204 countries and territories in 2021 for both genders combined, with AAPC from 1990 and 2021.

| **Location** | **Incidence** | | | **Prevalence** | | | **DALYs** | | | **Death** | | |
| --- | --- | --- | --- | --- | --- | --- | --- | --- | --- | --- | --- | --- |
|  | **Cases**  **(95% UI)** | **ASIR (per 100, 000) (95% UI)** | **AAPC % (95%CI) 1990–2019** | **Cases**  **(95% UI)** | **ASPR (per 100, 000) (95% UI)** | **AAPC % (95%CI) 1990–2019** | **Cases**  **(95% UI)** | **Age-standardized**  **DALY rate (per**  **100,000) (95% UI)** | **AAPC % (95%CI) 1990–2019** | **Cases (95% UI)** | **ASDR (per 100, 000) (95% UI)** | **AAPC % (95%CI) 1990–2019** |
| Afghanistan | 241.09 (341.29 to 162.40) | 6.65 (9.34 to 4.51) | 0.52 (0.49 to 0.55) | 1690.33 (2302.91 to 1203.92) | 46.56 (63.68 to 33.02) | 0.7 (0.68 to 0.72) | 1758.90 (2760.51 to 968.02) | 48.35 (75.72 to 26.66) | -0.36 (-0.44 to -0.28) | 36.70 (61.46 to 18.00) | 1.01 (1.69 to 0.50) | -0.52 (-0.61 to -0.42) |
| Albania | 23.21 (34.28 to 13.45) | 4.33 (6.47 to 2.47) | -0.02 (-0.07 to 0.03) | 149.22 (218.39 to 95.57) | 27.93 (41.05 to 17.83) | 0 (-0.02 to 0.02) | 57.90 (77.21 to 43.36) | 10.86 (14.50 to 8.13) | -0.54 (-1.04 to -0.04) | 0.83 (1.08 to 0.64) | 0.15 (0.20 to 0.12) | -0.92 (-1.65 to -0.19) |
| Algeria | 433.34 (633.68 to 267.63) | 5.67 (8.29 to 3.50) | 0.83 (0.79 to 0.88) | 3267.00 (4635.16 to 2192.23) | 42.92 (60.80 to 28.86) | 0.85 (0.82 to 0.88) | 1691.28 (2236.72 to 1267.15) | 22.32 (29.52 to 16.72) | 0.01 (-0.06 to 0.07) | 28.55 (38.57 to 20.13) | 0.38 (0.51 to 0.27) | -0.32 (-0.46 to -0.18) |
| American Samoa | 0.44 (0.65 to 0.27) | 4.42 (6.47 to 2.64) | 0.34 (0.25 to 0.44) | 3.03 (4.33 to 1.97) | 30.15 (43.17 to 19.57) | 0.33 (0.3 to 0.35) | 2.08 (2.72 to 1.59) | 20.41 (26.67 to 15.59) | -0.32 (-0.41 to -0.22) | 0.04 (0.05 to 0.03) | 0.39 (0.50 to 0.29) | -0.47 (-0.6 to -0.33) |
| Andorra | 1.52 (2.31 to 0.88) | 6.17 (9.43 to 3.56) | 0.56 (0.49 to 0.63) | 11.77 (17.07 to 7.75) | 47.77 (69.62 to 31.47) | 0.48 (0.43 to 0.53) | 3.38 (4.84 to 2.19) | 13.65 (19.60 to 8.81) | -0.41 (-0.58 to -0.23) | 0.04 (0.05 to 0.02) | 0.15 (0.22 to 0.10) | -1.32 (-1.49 to -1.15) |
| Angola | 130.32 (201.70 to 72.35) | 4.05 (6.23 to 2.26) | 0.31 (0.28 to 0.34) | 1037.56 (1540.07 to 672.56) | 32.28 (47.73 to 20.97) | 0.34 (0.29 to 0.38) | 628.97 (810.47 to 465.71) | 19.81 (25.52 to 14.67) | -0.27 (-0.38 to -0.17) | 11.22 (15.26 to 8.08) | 0.36 (0.49 to 0.26) | -0.46 (-0.59 to -0.33) |
| Antigua and Barbuda | 1.33 (2.02 to 0.81) | 6.75 (10.29 to 4.11) | 0.46 (0.27 to 0.65) | 11.33 (16.39 to 7.72) | 56.95 (82.54 to 38.70) | 0.27 (0.14 to 0.4) | 3.97 (5.21 to 3.02) | 19.81 (26.10 to 15.00) | 0.03 (-0.58 to 0.64) | 0.05 (0.06 to 0.05) | 0.27 (0.30 to 0.24) | -0.33 (-1.34 to 0.7) |
| Argentina | 368.19 (547.86 to 213.85) | 4.41 (6.55 to 2.57) | 0.5 (0.26 to 0.73) | 2322.13 (3401.90 to 1453.71) | 27.79 (40.68 to 17.41) | 0.68 (0.55 to 0.81) | 1051.10 (1330.87 to 851.62) | 12.60 (15.95 to 10.21) | -0.15 (-0.36 to 0.06) | 16.58 (17.74 to 15.40) | 0.20 (0.21 to 0.19) | -0.5 (-0.7 to -0.3) |
| Armenia | 20.18 (30.51 to 11.49) | 3.75 (5.68 to 2.13) | 0.03 (-0.09 to 0.15) | 142.13 (210.25 to 89.39) | 26.44 (39.07 to 16.66) | 0.03 (-0.01 to 0.07) | 56.07 (72.63 to 42.81) | 10.44 (13.51 to 7.97) | -0.56 (-0.92 to -0.21) | 0.81 (0.94 to 0.67) | 0.15 (0.18 to 0.12) | -0.83 (-1.59 to -0.06) |
| Australia | 161.09 (245.84 to 89.52) | 3.10 (4.75 to 1.70) | 0.65 (0.55 to 0.75) | 1068.75 (1609.41 to 659.38) | 20.69 (31.25 to 12.71) | 0.95 (0.84 to 1.06) | 635.83 (756.48 to 542.63) | 12.22 (14.57 to 10.42) | 0.36 (0.19 to 0.52) | 11.41 (12.18 to 10.63) | 0.22 (0.23 to 0.20) | 0.13 (-0.02 to 0.29) |
| Austria | 119.59 (177.29 to 68.22) | 5.82 (8.72 to 3.29) | 0.56 (0.52 to 0.6) | 929.20 (1340.73 to 614.91) | 45.02 (65.10 to 29.73) | 0.45 (0.39 to 0.51) | 281.46 (390.05 to 204.83) | 13.48 (18.76 to 9.76) | -0.4 (-0.59 to -0.2) | 3.38 (3.60 to 3.15) | 0.16 (0.17 to 0.15) | -1.1 (-1.42 to -0.78) |
| Azerbaijan | 73.31 (111.87 to 40.46) | 3.58 (5.49 to 1.96) | -0.1 (-0.3 to 0.09) | 525.23 (773.44 to 327.83) | 25.65 (37.83 to 15.98) | -0.11 (-0.14 to -0.07) | 216.13 (287.87 to 161.76) | 10.52 (14.01 to 7.87) | -1.06 (-1.24 to -0.89) | 3.22 (4.21 to 2.47) | 0.16 (0.20 to 0.12) | -1.52 (-2 to -1.03) |
| Bahamas | 5.76 (8.47 to 3.49) | 7.07 (10.41 to 4.28) | 0.36 (0.24 to 0.47) | 49.46 (69.39 to 33.78) | 60.35 (84.82 to 41.12) | 0.26 (0.15 to 0.36) | 23.23 (29.90 to 17.65) | 28.27 (36.40 to 21.47) | 0.21 (-0.22 to 0.64) | 0.38 (0.48 to 0.29) | 0.46 (0.59 to 0.35) | 0.24 (-0.41 to 0.91) |
| Bahrain | 19.40 (29.36 to 11.18) | 5.50 (8.25 to 3.21) | 0.59 (0.55 to 0.64) | 141.06 (206.76 to 92.66) | 40.19 (58.69 to 26.47) | 0.71 (0.67 to 0.74) | 57.43 (77.21 to 42.35) | 16.48 (22.07 to 12.17) | -1.15 (-1.31 to -0.98) | 0.84 (1.12 to 0.63) | 0.25 (0.33 to 0.18) | -2.02 (-2.3 to -1.73) |
| Bangladesh | 1395.63 (2122.23 to 791.74) | 5.30 (8.05 to 3.01) | 0.62 (0.53 to 0.71) | 11114.38 (16014.04 to 7365.84) | 42.28 (60.86 to 28.06) | 0.59 (0.54 to 0.64) | 4634.00 (6441.97 to 3197.86) | 17.70 (24.59 to 12.22) | -0.6 (-0.85 to -0.35) | 70.07 (106.61 to 47.25) | 0.27 (0.41 to 0.18) | -1.16 (-1.47 to -0.85) |
| Barbados | 4.12 (5.94 to 2.49) | 6.39 (9.24 to 3.87) | 0.53 (0.42 to 0.63) | 36.35 (50.61 to 25.13) | 55.66 (77.91 to 38.27) | 0.42 (0.35 to 0.49) | 12.29 (16.64 to 9.04) | 18.69 (25.36 to 13.69) | 0.09 (-0.26 to 0.44) | 0.16 (0.21 to 0.12) | 0.24 (0.32 to 0.18) | 0.03 (-0.43 to 0.49) |
| Belarus | 106.61 (162.15 to 60.90) | 5.34 (8.15 to 3.04) | 0.03 (-0.11 to 0.18) | 656.94 (972.74 to 418.32) | 32.94 (48.91 to 20.94) | 0.01 (-0.07 to 0.09) | 291.02 (374.43 to 223.59) | 14.57 (18.77 to 11.20) | 0.11 (-0.38 to 0.59) | 4.58 (5.68 to 3.63) | 0.23 (0.28 to 0.18) | 0.25 (-0.4 to 0.91) |
| Belgium | 147.26 (226.65 to 86.28) | 6.14 (9.50 to 3.58) | 0.69 (0.64 to 0.75) | 1153.38 (1687.13 to 763.52) | 47.98 (70.38 to 31.74) | 0.62 (0.55 to 0.68) | 363.71 (507.88 to 265.76) | 15.02 (21.03 to 10.94) | 0.21 (0.12 to 0.3) | 4.51 (4.84 to 4.20) | 0.18 (0.20 to 0.17) | -0.15 (-0.32 to 0.02) |
| Belize | 4.59 (6.75 to 2.77) | 6.92 (10.16 to 4.17) | 0.69 (0.6 to 0.78) | 38.59 (53.94 to 26.56) | 58.53 (81.68 to 40.32) | 0.51 (0.44 to 0.57) | 16.28 (20.75 to 12.75) | 24.74 (31.49 to 19.39) | 0.91 (0.62 to 1.2) | 0.25 (0.29 to 0.21) | 0.38 (0.44 to 0.32) | 1.1 (0.71 to 1.49) |
| Benin | 41.14 (65.37 to 21.77) | 3.20 (5.04 to 1.70) | 0.39 (0.33 to 0.46) | 367.56 (543.74 to 247.47) | 28.52 (42.06 to 19.28) | 0.46 (0.41 to 0.51) | 252.51 (364.93 to 170.88) | 18.75 (26.97 to 12.73) | 0.1 (-0.03 to 0.22) | 4.37 (6.52 to 2.87) | 0.32 (0.48 to 0.21) | -0.04 (-0.18 to 0.11) |
| Bermuda | 1.03 (1.51 to 0.62) | 6.94 (10.19 to 4.15) | 0.32 (0.1 to 0.55) | 8.96 (12.63 to 6.13) | 59.20 (83.87 to 40.34) | 0.26 (0.14 to 0.39) | 2.62 (3.78 to 1.78) | 17.22 (24.87 to 11.66) | -0.98 (-1.06 to -0.9) | 0.03 (0.04 to 0.02) | 0.19 (0.29 to 0.13) | -2 (-2.17 to -1.84) |
| Bhutan | 6.51 (9.96 to 3.72) | 5.47 (8.33 to 3.14) | 0.89 (0.78 to 1.01) | 52.88 (77.60 to 34.07) | 44.64 (65.29 to 28.87) | 0.73 (0.67 to 0.79) | 23.66 (31.43 to 17.14) | 20.12 (26.67 to 14.58) | -0.1 (-0.19 to -0.02) | 0.37 (0.52 to 0.26) | 0.32 (0.44 to 0.22) | -0.49 (-0.61 to -0.38) |
| Bolivia (Plurinational State of) | 303.51 (427.31 to 200.73) | 16.69 (23.50 to 11.02) | 1.23 (1.14 to 1.31) | 2316.90 (3174.49 to 1657.06) | 129.11 (176.56 to 92.57) | 0.51 (0.44 to 0.57) | 611.62 (847.01 to 420.77) | 34.12 (47.12 to 23.51) | -0.17 (-0.25 to -0.09) | 6.27 (8.79 to 4.37) | 0.35 (0.49 to 0.25) | -0.89 (-0.96 to -0.82) |
| Bosnia and Herzegovina | 32.32 (47.97 to 18.86) | 4.39 (6.55 to 2.55) | -0.37 (-0.42 to -0.31) | 195.61 (286.71 to 122.25) | 26.60 (39.08 to 16.59) | -0.17 (-0.22 to -0.13) | 96.31 (124.17 to 72.67) | 13.03 (16.81 to 9.82) | -0.44 (-0.88 to -0.01) | 1.61 (2.05 to 1.17) | 0.22 (0.28 to 0.16) | -0.54 (-1.17 to 0.1) |
| Botswana | 14.35 (22.50 to 8.01) | 3.92 (6.10 to 2.19) | 0.47 (0.39 to 0.56) | 119.76 (174.83 to 80.28) | 32.94 (47.95 to 22.16) | 0.4 (0.35 to 0.45) | 59.06 (79.10 to 43.08) | 16.51 (22.07 to 12.03) | -0.73 (-1.16 to -0.3) | 0.96 (1.33 to 0.67) | 0.27 (0.38 to 0.19) | -1.16 (-1.76 to -0.56) |
| Brazil | 3154.51 (4503.82 to 2001.70) | 7.26 (10.37 to 4.60) | 1.03 (0.91 to 1.15) | 27087.00 (36554.15 to 19150.55) | 62.36 (84.14 to 44.12) | 0.87 (0.64 to 1.09) | 9360.96 (11490.81 to 7684.24) | 21.56 (26.46 to 17.71) | 0.36 (0.31 to 0.42) | 126.01 (130.65 to 121.52) | 0.29 (0.30 to 0.28) | 0 (-0.22 to 0.22) |
| Brunei Darussalam | 4.72 (6.95 to 2.88) | 5.27 (7.74 to 3.23) | 0.6 (0.42 to 0.78) | 31.72 (45.33 to 20.74) | 35.38 (50.42 to 23.18) | 0.75 (0.69 to 0.8) | 22.21 (28.59 to 14.93) | 24.96 (32.10 to 16.79) | 0.5 (0.39 to 0.61) | 0.42 (0.56 to 0.26) | 0.47 (0.63 to 0.29) | 0.44 (0.29 to 0.59) |
| Bulgaria | 65.37 (96.04 to 37.32) | 4.07 (6.00 to 2.32) | -0.05 (-0.21 to 0.1) | 407.98 (583.26 to 259.75) | 25.43 (36.46 to 16.16) | 0.22 (0.14 to 0.3) | 280.79 (338.15 to 233.94) | 17.44 (21.01 to 14.52) | 0.73 (0.54 to 0.93) | 5.29 (6.27 to 4.40) | 0.33 (0.39 to 0.27) | 0.93 (0.71 to 1.15) |
| Burkina Faso | 65.17 (102.80 to 34.28) | 3.04 (4.79 to 1.60) | 0.4 (0.37 to 0.42) | 584.08 (847.91 to 375.23) | 27.27 (39.49 to 17.56) | 0.46 (0.42 to 0.49) | 412.52 (586.93 to 272.16) | 18.41 (26.08 to 12.18) | 0.23 (0.07 to 0.38) | 7.23 (10.76 to 4.50) | 0.32 (0.48 to 0.20) | 0.13 (-0.09 to 0.36) |
| Burundi | 44.35 (68.88 to 23.96) | 3.62 (5.61 to 1.95) | 0.02 (-0.05 to 0.08) | 382.49 (567.20 to 251.67) | 31.33 (46.30 to 20.69) | 0.13 (0.11 to 0.16) | 214.97 (298.35 to 140.18) | 17.90 (24.87 to 11.67) | -0.75 (-0.85 to -0.65) | 3.71 (5.49 to 2.19) | 0.31 (0.46 to 0.19) | -1.02 (-1.16 to -0.88) |
| Cabo Verde | 3.14 (4.93 to 1.58) | 3.58 (5.62 to 1.80) | 1.08 (0.85 to 1.3) | 29.57 (42.39 to 19.36) | 33.74 (48.32 to 22.11) | 0.97 (0.92 to 1.03) | 19.70 (27.19 to 14.62) | 21.97 (30.30 to 16.31) | 0.84 (0.67 to 1.02) | 0.34 (0.47 to 0.24) | 0.38 (0.52 to 0.27) | 0.83 (0.63 to 1.03) |
| Cambodia | 115.29 (173.11 to 67.28) | 4.64 (6.97 to 2.70) | 0.33 (0.3 to 0.35) | 849.37 (1239.78 to 558.56) | 34.18 (49.82 to 22.50) | 0.29 (0.26 to 0.32) | 597.97 (808.47 to 440.80) | 24.07 (32.55 to 17.73) | -0.25 (-0.33 to -0.16) | 11.17 (15.52 to 7.91) | 0.45 (0.63 to 0.32) | -0.4 (-0.46 to -0.33) |
| Cameroon | 109.72 (173.97 to 57.05) | 3.40 (5.36 to 1.78) | 0.47 (0.3 to 0.64) | 977.26 (1428.07 to 646.78) | 30.29 (44.21 to 20.11) | 0.49 (0.45 to 0.53) | 820.20 (1209.61 to 530.19) | 24.04 (35.19 to 15.64) | 0.4 (0.33 to 0.47) | 14.95 (23.61 to 8.93) | 0.44 (0.69 to 0.26) | 0.32 (0.21 to 0.43) |
| Canada | 749.36 (954.30 to 545.32) | 9.98 (12.78 to 7.21) | 1.43 (1.35 to 1.52) | 4829.49 (6144.27 to 3635.75) | 64.14 (81.72 to 48.15) | 1.47 (1.32 to 1.61) | 1693.36 (2200.02 to 1322.57) | 22.45 (29.17 to 17.52) | 0.75 (0.51 to 0.99) | 23.19 (24.90 to 21.60) | 0.30 (0.33 to 0.28) | 0.49 (0.25 to 0.72) |
| Central African Republic | 28.26 (41.01 to 16.97) | 4.41 (6.39 to 2.66) | 0.16 (0.08 to 0.24) | 216.71 (306.83 to 143.18) | 33.92 (47.88 to 22.44) | 0.22 (0.17 to 0.28) | 158.59 (227.31 to 104.04) | 25.06 (35.90 to 16.45) | -0.26 (-0.43 to -0.09) | 3.02 (4.66 to 1.74) | 0.48 (0.74 to 0.28) | -0.38 (-0.58 to -0.18) |
| Chad | 43.67 (68.74 to 22.73) | 3.01 (4.71 to 1.58) | 0.49 (0.27 to 0.71) | 390.70 (562.12 to 254.74) | 26.86 (38.54 to 17.53) | 0.49 (0.37 to 0.61) | 299.29 (415.58 to 210.62) | 19.84 (27.46 to 13.99) | 1.01 (0.83 to 1.19) | 5.37 (7.61 to 3.64) | 0.36 (0.50 to 0.24) | 1.17 (0.94 to 1.4) |
| Chile | 176.13 (258.87 to 100.59) | 4.68 (6.90 to 2.66) | 1.08 (0.85 to 1.31) | 1064.00 (1536.21 to 678.21) | 28.40 (41.14 to 18.06) | 1.04 (0.89 to 1.18) | 476.76 (607.55 to 385.53) | 12.68 (16.18 to 10.24) | -0.36 (-0.73 to 0.02) | 7.57 (8.12 to 7.09) | 0.20 (0.21 to 0.19) | -0.89 (-1.65 to -0.12) |
| China | 70981.31 (96610.61 to 50141.52) | 19.95 (27.29 to 13.98) | 3.94 (3.76 to 4.11) | 429706.07 (581420.52 to 309316.55) | 116.54 (158.08 to 83.82) | 3.54 (3.42 to 3.65) | 126812.05 (165923.29 to 97265.65) | 35.03 (45.77 to 26.96) | 0.1 (0.04 to 0.16) | 1489.12 (1900.99 to 1176.76) | 0.41 (0.53 to 0.33) | -1.53 (-1.62 to -1.45) |
| Colombia | 654.67 (951.07 to 388.24) | 7.37 (10.74 to 4.37) | 0.88 (0.68 to 1.09) | 5583.33 (7808.29 to 3779.34) | 62.51 (87.51 to 42.29) | 0.79 (0.66 to 0.91) | 1708.15 (2335.66 to 1258.02) | 19.11 (26.13 to 14.07) | -0.13 (-0.26 to 0) | 20.29 (24.94 to 16.22) | 0.23 (0.28 to 0.18) | -0.92 (-1.27 to -0.56) |
| Comoros | 4.03 (6.41 to 2.22) | 3.60 (5.71 to 1.99) | 0.1 (0.02 to 0.19) | 34.48 (51.96 to 22.52) | 30.84 (46.42 to 20.16) | 0.12 (0.08 to 0.16) | 19.86 (26.44 to 14.01) | 17.84 (23.75 to 12.58) | -0.44 (-0.75 to -0.14) | 0.35 (0.49 to 0.24) | 0.31 (0.44 to 0.21) | -0.61 (-1.03 to -0.19) |
| Congo | 35.57 (53.60 to 20.49) | 4.56 (6.84 to 2.64) | 0.14 (-0.01 to 0.3) | 275.79 (396.36 to 178.32) | 35.47 (50.77 to 23.00) | 0.16 (0.12 to 0.2) | 172.26 (231.47 to 125.15) | 22.41 (30.08 to 16.30) | -0.73 (-0.94 to -0.51) | 3.11 (4.45 to 2.07) | 0.41 (0.59 to 0.27) | -0.97 (-1.24 to -0.7) |
| Cook Islands | 0.20 (0.29 to 0.12) | 5.61 (8.27 to 3.35) | 0.18 (0.08 to 0.27) | 1.34 (1.94 to 0.86) | 37.24 (54.05 to 23.81) | 0.26 (0.22 to 0.3) | 0.64 (0.90 to 0.46) | 17.40 (24.57 to 12.54) | -0.81 (-0.88 to -0.74) | 0.01 (0.02 to 0.01) | 0.29 (0.43 to 0.19) | -1.25 (-1.43 to -1.06) |
| Costa Rica | 74.29 (110.30 to 45.41) | 8.51 (12.65 to 5.18) | 0.77 (0.64 to 0.9) | 614.85 (867.56 to 416.25) | 70.13 (98.98 to 47.47) | 0.72 (0.69 to 0.75) | 190.76 (262.09 to 139.03) | 21.77 (29.93 to 15.86) | 0.49 (0.25 to 0.73) | 2.34 (2.66 to 2.01) | 0.27 (0.30 to 0.23) | 0.23 (-0.28 to 0.74) |
| Coted'Ivoire | 104.83 (163.24 to 54.48) | 3.37 (5.22 to 1.76) | 0.38 (0.31 to 0.45) | 938.35 (1344.62 to 621.18) | 30.17 (43.08 to 20.02) | 0.37 (0.34 to 0.41) | 756.36 (1092.74 to 515.23) | 22.83 (32.71 to 15.65) | 0.15 (-0.12 to 0.43) | 13.61 (20.68 to 8.70) | 0.41 (0.62 to 0.26) | 0.07 (-0.28 to 0.42) |
| Croatia | 38.62 (58.86 to 22.53) | 4.21 (6.46 to 2.43) | 0.07 (0.04 to 0.11) | 237.27 (354.70 to 146.39) | 25.90 (38.82 to 15.93) | 0.08 (0.06 to 0.09) | 102.10 (130.54 to 80.30) | 11.10 (14.20 to 8.73) | -0.67 (-1.19 to -0.15) | 1.59 (1.85 to 1.33) | 0.17 (0.20 to 0.14) | -1 (-1.72 to -0.27) |
| Cuba | 169.71 (251.67 to 99.03) | 6.22 (9.29 to 3.61) | 1.02 (0.76 to 1.28) | 1484.40 (2097.03 to 990.75) | 52.91 (75.54 to 35.00) | 0.71 (0.61 to 0.81) | 547.13 (708.90 to 417.85) | 19.09 (24.96 to 14.43) | 0.6 (0.38 to 0.82) | 7.73 (8.99 to 6.50) | 0.26 (0.30 to 0.22) | 0.6 (0.22 to 0.97) |
| Cyprus | 17.15 (26.39 to 9.99) | 6.04 (9.30 to 3.52) | 0.01 (-0.04 to 0.07) | 125.91 (187.76 to 81.13) | 44.34 (66.08 to 28.59) | 0.06 (0.04 to 0.08) | 34.04 (50.32 to 22.96) | 12.01 (17.75 to 8.10) | -0.95 (-1.19 to -0.7) | 0.34 (0.44 to 0.27) | 0.12 (0.16 to 0.10) | -1.96 (-2.51 to -1.41) |
| Czechia | 101.73 (160.94 to 55.14) | 4.12 (6.47 to 2.25) | 0.2 (0.16 to 0.24) | 634.90 (968.98 to 392.99) | 25.64 (39.20 to 15.87) | 0.21 (0.18 to 0.24) | 261.71 (341.96 to 205.85) | 10.58 (13.85 to 8.31) | -0.78 (-1.02 to -0.54) | 3.88 (4.46 to 3.27) | 0.16 (0.18 to 0.13) | -1.35 (-1.65 to -1.05) |
| Democratic People's Republic of Korea | 787.53 (1081.75 to 541.98) | 12.23 (16.88 to 8.34) | 1.78 (1.7 to 1.87) | 4677.40 (6262.27 to 3292.98) | 71.70 (96.64 to 50.28) | 1.58 (1.55 to 1.62) | 2784.80 (3868.48 to 1910.36) | 43.17 (60.07 to 29.61) | 0.43 (0.37 to 0.48) | 50.59 (75.03 to 32.22) | 0.78 (1.16 to 0.49) | 0.09 (0.03 to 0.16) |
| Democratic Republic of the Congo | 382.78 (587.52 to 214.80) | 3.99 (6.11 to 2.24) | 0.45 (0.3 to 0.59) | 3049.94 (4430.81 to 1970.42) | 31.86 (46.19 to 20.62) | 0.46 (0.44 to 0.49) | 1821.38 (2448.43 to 1271.27) | 19.22 (25.85 to 13.41) | -0.05 (-0.15 to 0.04) | 32.53 (46.51 to 21.41) | 0.35 (0.50 to 0.23) | -0.24 (-0.37 to -0.1) |
| Denmark | 75.01 (114.18 to 44.06) | 6.12 (9.37 to 3.57) | 0.9 (0.82 to 0.98) | 583.87 (841.98 to 386.78) | 47.48 (68.61 to 31.43) | 0.82 (0.79 to 0.84) | 161.02 (230.88 to 109.27) | 12.97 (18.67 to 8.75) | -0.07 (-0.16 to 0.02) | 1.73 (1.84 to 1.62) | 0.14 (0.15 to 0.13) | -0.93 (-1.08 to -0.77) |
| Djibouti | 7.05 (10.90 to 4.01) | 3.67 (5.64 to 2.10) | 0.46 (0.43 to 0.49) | 59.59 (87.10 to 39.04) | 31.16 (45.42 to 20.48) | 0.36 (0.31 to 0.41) | 31.48 (46.74 to 21.55) | 16.66 (24.78 to 11.41) | 0.15 (0.07 to 0.22) | 0.53 (0.86 to 0.32) | 0.28 (0.46 to 0.17) | 0.08 (-0.03 to 0.18) |
| Dominica | 0.82 (1.20 to 0.50) | 6.32 (9.34 to 3.82) | 0.42 (0.31 to 0.54) | 7.31 (10.33 to 5.03) | 55.10 (78.24 to 37.71) | 0.35 (0.25 to 0.45) | 3.47 (4.61 to 2.62) | 25.73 (34.21 to 19.33) | 0.25 (0.18 to 0.31) | 0.06 (0.08 to 0.04) | 0.42 (0.56 to 0.30) | 0.21 (0.12 to 0.3) |
| Dominican Republic | 115.71 (171.78 to 70.02) | 6.26 (9.29 to 3.79) | 0.91 (0.8 to 1.02) | 1013.03 (1432.39 to 691.19) | 54.89 (77.60 to 37.47) | 0.67 (0.57 to 0.76) | 429.65 (564.64 to 322.42) | 23.30 (30.62 to 17.49) | 0.52 (0.2 to 0.85) | 6.60 (8.71 to 4.93) | 0.36 (0.47 to 0.27) | 0.55 (0.04 to 1.06) |
| Ecuador | 450.49 (635.70 to 296.77) | 15.53 (21.91 to 10.23) | 2.11 (2.04 to 2.18) | 3590.83 (4933.75 to 2546.85) | 124.63 (170.98 to 88.49) | 1.56 (1.54 to 1.59) | 880.23 (1240.78 to 607.58) | 30.57 (43.04 to 21.13) | 0.85 (0.62 to 1.08) | 7.83 (10.19 to 5.84) | 0.27 (0.35 to 0.20) | 0.07 (-0.56 to 0.7) |
| Egypt | 1259.79 (1747.60 to 859.10) | 8.39 (11.63 to 5.73) | 1.49 (1.44 to 1.54) | 8117.55 (11225.66 to 5631.71) | 54.37 (74.99 to 37.84) | 1.33 (1.24 to 1.41) | 3455.40 (4643.19 to 2605.26) | 23.21 (31.20 to 17.49) | -0.55 (-0.82 to -0.28) | 53.69 (71.29 to 40.64) | 0.36 (0.48 to 0.27) | -1.28 (-1.71 to -0.86) |
| El Salvador | 75.29 (114.16 to 44.44) | 7.42 (11.25 to 4.38) | 0.87 (0.64 to 1.1) | 643.29 (951.36 to 433.86) | 63.51 (93.90 to 42.84) | 0.64 (0.57 to 0.7) | 278.95 (361.02 to 209.11) | 27.54 (35.64 to 20.65) | 0.21 (-0.46 to 0.88) | 4.40 (5.69 to 3.27) | 0.43 (0.56 to 0.32) | 0.08 (-0.96 to 1.13) |
| Equatorial Guinea | 6.24 (9.63 to 3.50) | 4.38 (6.73 to 2.46) | 0.49 (0.38 to 0.59) | 48.27 (70.48 to 31.25) | 33.95 (49.25 to 22.07) | 0.45 (0.41 to 0.5) | 28.52 (40.40 to 19.89) | 20.39 (28.94 to 14.21) | -0.53 (-0.72 to -0.35) | 0.50 (0.79 to 0.32) | 0.37 (0.57 to 0.23) | -0.85 (-1.07 to -0.63) |
| Eritrea | 32.39 (49.95 to 18.56) | 4.06 (6.23 to 2.34) | 0.21 (0.17 to 0.26) | 272.50 (394.70 to 180.27) | 34.29 (49.55 to 22.76) | 0.23 (0.21 to 0.25) | 181.94 (249.36 to 129.95) | 23.19 (31.74 to 16.57) | -0.25 (-0.41 to -0.1) | 3.34 (4.90 to 2.25) | 0.43 (0.63 to 0.29) | -0.43 (-0.66 to -0.2) |
| Estonia | 16.11 (23.85 to 9.70) | 5.95 (8.83 to 3.57) | -0.36 (-0.4 to -0.31) | 102.43 (148.91 to 66.51) | 37.80 (55.10 to 24.50) | -0.3 (-0.34 to -0.26) | 32.25 (44.67 to 23.14) | 11.87 (16.46 to 8.52) | -0.91 (-1.25 to -0.58) | 0.40 (0.46 to 0.34) | 0.14 (0.17 to 0.13) | -1.27 (-2.59 to 0.07) |
| Eswatini | 5.95 (8.86 to 3.69) | 4.37 (6.48 to 2.71) | 0.47 (0.4 to 0.55) | 49.97 (69.38 to 34.03) | 36.99 (50.95 to 25.33) | 0.43 (0.39 to 0.47) | 45.14 (65.75 to 28.04) | 33.97 (49.56 to 21.16) | 1.04 (0.84 to 1.23) | 0.90 (1.39 to 0.50) | 0.68 (1.06 to 0.38) | 1.18 (0.97 to 1.39) |
| Ethiopia | 380.62 (571.09 to 221.38) | 3.55 (5.30 to 2.07) | -0.27 (-0.39 to -0.15) | 3340.80 (4669.64 to 2319.30) | 31.32 (43.62 to 21.83) | -0.18 (-0.27 to -0.09) | 1536.70 (2216.31 to 1100.56) | 14.65 (21.13 to 10.49) | -1.61 (-1.72 to -1.5) | 24.01 (37.47 to 16.07) | 0.23 (0.36 to 0.16) | -2.17 (-2.26 to -2.07) |
| Fiji | 8.88 (12.64 to 5.61) | 5.62 (8.01 to 3.54) | 0.29 (0.2 to 0.37) | 56.99 (80.08 to 37.78) | 36.09 (50.67 to 23.95) | 0.3 (0.27 to 0.32) | 34.23 (45.86 to 25.60) | 21.63 (28.98 to 16.18) | -0.48 (-0.71 to -0.26) | 0.63 (0.85 to 0.44) | 0.40 (0.54 to 0.28) | -0.69 (-0.99 to -0.39) |
| Finland | 67.27 (103.57 to 39.84) | 6.41 (9.93 to 3.77) | 0.79 (0.75 to 0.84) | 517.98 (757.88 to 343.73) | 49.24 (72.13 to 32.63) | 0.67 (0.63 to 0.7) | 163.40 (224.24 to 118.24) | 15.43 (21.26 to 11.13) | -0.18 (-0.29 to -0.06) | 2.02 (2.17 to 1.88) | 0.19 (0.20 to 0.17) | -0.79 (-0.99 to -0.58) |
| France | 813.05 (1242.08 to 461.43) | 6.00 (9.21 to 3.38) | 0.68 (0.64 to 0.73) | 6231.26 (9098.17 to 4038.96) | 45.88 (67.26 to 29.69) | 0.54 (0.52 to 0.57) | 2104.63 (2875.80 to 1537.15) | 15.39 (21.11 to 11.20) | 0.07 (-0.05 to 0.2) | 28.02 (30.34 to 25.95) | 0.20 (0.22 to 0.19) | -0.22 (-0.4 to -0.04) |
| Gabon | 12.05 (18.02 to 7.27) | 4.91 (7.34 to 2.97) | 0.24 (0.15 to 0.32) | 92.63 (133.37 to 62.91) | 37.83 (54.42 to 25.73) | 0.26 (0.16 to 0.36) | 58.80 (79.86 to 42.63) | 24.09 (32.71 to 17.47) | -0.47 (-0.64 to -0.3) | 1.07 (1.55 to 0.73) | 0.44 (0.64 to 0.30) | -0.69 (-1.01 to -0.37) |
| Gambia | 7.52 (11.77 to 3.88) | 3.24 (5.03 to 1.68) | 0.65 (0.54 to 0.77) | 66.47 (96.08 to 43.55) | 28.62 (41.24 to 18.78) | 0.59 (0.51 to 0.66) | 48.96 (74.41 to 31.54) | 19.89 (29.86 to 12.89) | 0.74 (0.06 to 1.43) | 0.86 (1.41 to 0.53) | 0.35 (0.56 to 0.22) | 0.8 (-0.11 to 1.73) |
| Georgia | 29.56 (45.46 to 16.47) | 4.17 (6.43 to 2.31) | -0.11 (-0.16 to -0.07) | 206.50 (303.89 to 129.47) | 29.11 (42.93 to 18.21) | -0.07 (-0.1 to -0.05) | 103.02 (129.70 to 83.25) | 14.47 (18.24 to 11.68) | -0.06 (-0.27 to 0.14) | 1.72 (1.95 to 1.50) | 0.24 (0.27 to 0.21) | 0 (-0.3 to 0.3) |
| Germany | 1315.53 (1709.77 to 937.12) | 7.30 (9.66 to 5.09) | 0.9 (0.81 to 0.99) | 9705.49 (12728.35 to 6997.30) | 53.34 (70.46 to 38.24) | 0.73 (0.7 to 0.77) | 2994.29 (4086.17 to 2206.01) | 16.30 (22.40 to 11.95) | 0.23 (0.09 to 0.37) | 36.23 (38.57 to 33.98) | 0.19 (0.21 to 0.18) | -0.42 (-0.68 to -0.15) |
| Ghana | 122.89 (197.46 to 64.35) | 2.97 (4.74 to 1.56) | 0.51 (0.43 to 0.59) | 1074.47 (1586.02 to 688.95) | 25.89 (38.14 to 16.63) | 0.35 (0.32 to 0.39) | 733.32 (1029.15 to 522.97) | 16.97 (23.74 to 12.12) | 0.32 (0.22 to 0.43) | 12.75 (18.09 to 8.81) | 0.29 (0.42 to 0.20) | 0.33 (0.24 to 0.43) |
| Greece | 143.24 (220.28 to 84.54) | 5.91 (9.13 to 3.47) | 0.63 (0.58 to 0.69) | 1103.57 (1590.75 to 739.27) | 45.42 (65.58 to 30.41) | 0.54 (0.51 to 0.57) | 378.28 (508.09 to 281.64) | 15.46 (20.83 to 11.46) | 0.79 (0.7 to 0.89) | 5.11 (5.48 to 4.74) | 0.21 (0.22 to 0.19) | 1.11 (0.95 to 1.27) |
| Greenland | 0.69 (0.94 to 0.47) | 5.86 (8.07 to 3.87) | 0.26 (0.2 to 0.31) | 4.03 (5.48 to 2.79) | 33.83 (46.43 to 23.27) | 0.27 (0.26 to 0.28) | 3.31 (4.31 to 2.24) | 27.64 (35.96 to 18.70) | -0.74 (-0.84 to -0.65) | 0.07 (0.09 to 0.04) | 0.55 (0.74 to 0.35) | -0.91 (-1.03 to -0.79) |
| Grenada | 1.30 (1.95 to 0.78) | 6.11 (9.16 to 3.69) | 0.67 (0.48 to 0.87) | 11.74 (16.34 to 8.10) | 53.87 (75.42 to 36.97) | 0.49 (0.39 to 0.6) | 5.01 (6.40 to 3.85) | 22.65 (29.06 to 17.32) | 0.18 (0.02 to 0.34) | 0.08 (0.09 to 0.07) | 0.35 (0.42 to 0.29) | -0.1 (-0.65 to 0.46) |
| Guam | 1.94 (2.85 to 1.19) | 6.14 (9.13 to 3.77) | 0.48 (0.35 to 0.62) | 12.79 (18.86 to 8.66) | 40.34 (59.76 to 27.21) | 0.52 (0.43 to 0.6) | 5.94 (7.75 to 4.48) | 18.45 (24.13 to 13.88) | 0.18 (0.06 to 0.3) | 0.10 (0.12 to 0.08) | 0.30 (0.38 to 0.23) | 0.04 (-0.15 to 0.24) |
| Guatemala | 139.09 (203.78 to 83.60) | 6.50 (9.52 to 3.90) | 0.67 (0.54 to 0.8) | 1212.60 (1694.43 to 822.10) | 57.25 (79.77 to 38.98) | 0.61 (0.55 to 0.66) | 456.11 (603.09 to 350.91) | 21.58 (28.45 to 16.63) | -0.17 (-0.66 to 0.33) | 6.42 (7.66 to 5.28) | 0.31 (0.37 to 0.25) | -0.57 (-1.4 to 0.27) |
| Guinea | 39.22 (62.66 to 20.27) | 3.14 (4.99 to 1.63) | 0.42 (0.39 to 0.45) | 340.02 (503.83 to 219.45) | 27.18 (40.20 to 17.59) | 0.42 (0.35 to 0.49) | 245.75 (360.55 to 168.33) | 18.90 (27.53 to 12.99) | 0.51 (0.42 to 0.6) | 4.36 (6.60 to 2.80) | 0.34 (0.50 to 0.22) | 0.53 (0.42 to 0.63) |
| Guinea-Bissau | 6.77 (10.52 to 3.71) | 3.45 (5.34 to 1.90) | 0.24 (0.15 to 0.32) | 59.65 (86.09 to 38.95) | 30.46 (43.86 to 19.96) | 0.28 (0.24 to 0.33) | 61.86 (86.01 to 41.68) | 29.50 (40.85 to 19.95) | 0.04 (-0.02 to 0.11) | 1.18 (1.69 to 0.76) | 0.56 (0.80 to 0.37) | -0.03 (-0.1 to 0.04) |
| Guyana | 8.43 (11.88 to 5.31) | 6.55 (9.23 to 4.12) | 0.66 (0.44 to 0.88) | 71.18 (99.45 to 49.68) | 55.13 (77.06 to 38.45) | 0.39 (0.29 to 0.49) | 37.96 (49.73 to 28.48) | 29.34 (38.46 to 22.01) | 0.77 (0.48 to 1.06) | 0.66 (0.87 to 0.48) | 0.51 (0.67 to 0.37) | 0.93 (0.41 to 1.45) |
| Haiti | 106.73 (160.04 to 65.64) | 6.02 (9.01 to 3.70) | 0.19 (0.09 to 0.3) | 914.24 (1290.88 to 627.52) | 52.19 (73.55 to 35.98) | 0.17 (0.05 to 0.3) | 484.90 (676.76 to 331.72) | 28.08 (39.23 to 19.25) | -0.33 (-0.53 to -0.13) | 8.30 (12.36 to 5.02) | 0.49 (0.73 to 0.30) | -0.52 (-0.65 to -0.4) |
| Honduras | 110.28 (164.26 to 70.81) | 8.19 (12.16 to 5.27) | 0.96 (0.87 to 1.04) | 876.70 (1237.39 to 602.15) | 65.82 (92.65 to 45.38) | 0.7 (0.65 to 0.74) | 469.98 (631.03 to 331.68) | 35.49 (47.55 to 25.11) | 0.16 (0.11 to 0.22) | 8.12 (11.36 to 5.62) | 0.62 (0.86 to 0.43) | 0.03 (-0.05 to 0.11) |
| Hungary | 93.39 (146.39 to 51.82) | 4.03 (6.31 to 2.25) | 0.37 (0.34 to 0.41) | 580.32 (870.58 to 350.62) | 25.04 (37.61 to 15.10) | 0.3 (0.28 to 0.31) | 299.60 (368.93 to 243.26) | 12.90 (15.90 to 10.47) | -0.76 (-1.29 to -0.23) | 5.09 (5.85 to 4.33) | 0.22 (0.25 to 0.19) | -1.12 (-1.77 to -0.46) |
| Iceland | 4.47 (6.81 to 2.55) | 6.63 (10.13 to 3.78) | 0.65 (0.57 to 0.73) | 34.46 (49.61 to 23.17) | 51.12 (73.64 to 34.34) | 0.6 (0.55 to 0.65) | 10.47 (14.31 to 7.49) | 15.55 (21.26 to 11.14) | -0.16 (-0.33 to 0.01) | 0.12 (0.14 to 0.11) | 0.18 (0.20 to 0.16) | -0.84 (-1.13 to -0.55) |
| India | 14643.81 (21226.48 to 9254.21) | 6.24 (9.04 to 3.95) | 0.95 (0.83 to 1.06) | 121616.19 (167495.69 to 85925.12) | 51.96 (71.53 to 36.75) | 0.83 (0.77 to 0.89) | 50923.57 (62966.74 to 40165.13) | 21.79 (26.93 to 17.19) | 0.28 (0.09 to 0.47) | 779.72 (955.67 to 623.41) | 0.34 (0.41 to 0.27) | -0.02 (-0.31 to 0.27) |
| Indonesia | 2546.75 (3694.23 to 1595.38) | 4.57 (6.62 to 2.86) | 0.44 (0.32 to 0.56) | 19013.11 (26508.87 to 13185.92) | 34.10 (47.54 to 23.64) | 0.32 (0.27 to 0.38) | 12537.09 (15707.99 to 9895.57) | 22.48 (28.17 to 17.74) | 0 (-0.05 to 0.06) | 229.05 (299.27 to 176.50) | 0.41 (0.54 to 0.32) | -0.1 (-0.16 to -0.05) |
| Iran (Islamic Republic of) | 1005.62 (1437.95 to 652.87) | 5.93 (8.48 to 3.84) | 0.93 (0.84 to 1.01) | 7556.58 (10374.97 to 5329.15) | 44.79 (61.34 to 31.67) | 0.9 (0.84 to 0.97) | 3400.31 (4144.89 to 2211.71) | 20.31 (24.71 to 13.16) | -0.46 (-0.61 to -0.31) | 54.03 (62.19 to 25.38) | 0.33 (0.37 to 0.15) | -1.01 (-1.21 to -0.82) |
| Iraq | 355.60 (521.75 to 222.96) | 5.65 (8.26 to 3.55) | 1.07 (1.01 to 1.13) | 2609.35 (3708.88 to 1778.53) | 41.65 (59.07 to 28.44) | 1.06 (1.04 to 1.09) | 1865.89 (2475.86 to 1398.42) | 29.93 (39.69 to 22.43) | -0.39 (-0.43 to -0.34) | 35.36 (48.84 to 24.96) | 0.57 (0.79 to 0.40) | -0.7 (-0.75 to -0.65) |
| Ireland | 72.10 (110.68 to 40.79) | 6.70 (10.29 to 3.79) | 0.65 (0.6 to 0.7) | 546.36 (789.89 to 358.26) | 50.75 (73.39 to 33.27) | 0.59 (0.58 to 0.61) | 148.02 (211.06 to 101.41) | 13.74 (19.60 to 9.41) | -0.14 (-0.44 to 0.16) | 1.54 (1.67 to 1.42) | 0.14 (0.15 to 0.13) | -0.81 (-1.26 to -0.37) |
| Israel | 126.95 (192.32 to 74.30) | 7.74 (11.70 to 4.55) | 0.15 (-0.04 to 0.34) | 933.18 (1335.35 to 614.95) | 56.97 (81.56 to 37.56) | 0.32 (0.22 to 0.42) | 244.79 (358.35 to 163.43) | 14.95 (21.89 to 9.98) | -0.08 (-0.46 to 0.31) | 2.39 (2.58 to 2.20) | 0.15 (0.16 to 0.14) | -0.51 (-1.12 to 0.1) |
| Italy | 668.05 (998.86 to 378.53) | 4.56 (6.86 to 2.57) | -0.7 (-0.86 to -0.54) | 6383.61 (8714.96 to 4528.46) | 43.82 (59.92 to 31.08) | -0.23 (-0.38 to -0.09) | 2147.50 (2673.84 to 1743.19) | 14.44 (18.10 to 11.65) | -0.6 (-0.85 to -0.36) | 28.25 (29.17 to 27.32) | 0.18 (0.19 to 0.18) | -0.99 (-1.34 to -0.64) |
| Jamaica | 32.23 (48.69 to 19.58) | 6.41 (9.70 to 3.89) | 0.56 (0.44 to 0.67) | 280.68 (401.64 to 188.35) | 55.53 (79.57 to 37.19) | 0.46 (0.36 to 0.57) | 103.17 (141.98 to 74.35) | 20.32 (27.98 to 14.63) | 1.05 (-0.11 to 2.23) | 1.45 (1.95 to 1.07) | 0.28 (0.38 to 0.21) | 1.22 (-1.41 to 3.91) |
| Japan | 1053.18 (1523.61 to 636.40) | 3.62 (5.24 to 2.19) | -0.3 (-0.41 to -0.18) | 8684.34 (12078.57 to 6099.37) | 29.65 (41.48 to 20.73) | 0.05 (-0.09 to 0.2) | 4274.40 (5000.76 to 3690.15) | 14.37 (16.89 to 12.36) | 0.09 (-0.11 to 0.3) | 71.06 (72.60 to 69.28) | 0.24 (0.24 to 0.23) | 0.13 (-0.2 to 0.47) |
| Jordan | 87.12 (126.60 to 53.78) | 4.27 (6.20 to 2.64) | 0.29 (0.22 to 0.36) | 631.20 (893.25 to 429.19) | 30.98 (43.77 to 21.11) | 0.29 (0.2 to 0.37) | 339.01 (444.43 to 254.53) | 16.67 (21.85 to 12.52) | -1.27 (-1.45 to -1.09) | 5.80 (7.57 to 4.37) | 0.29 (0.37 to 0.22) | -1.78 (-1.99 to -1.56) |
| Kazakhstan | 149.56 (228.23 to 86.36) | 4.42 (6.73 to 2.55) | 0.1 (0.03 to 0.17) | 1064.88 (1555.79 to 683.84) | 31.43 (45.93 to 20.18) | 0.15 (0.12 to 0.18) | 549.06 (685.73 to 442.71) | 16.21 (20.24 to 13.07) | -0.03 (-0.5 to 0.43) | 9.20 (10.68 to 7.78) | 0.27 (0.32 to 0.23) | -0.15 (-0.82 to 0.52) |
| Kenya | 223.32 (332.43 to 134.71) | 3.88 (5.75 to 2.34) | 0.55 (0.51 to 0.59) | 1953.72 (2696.75 to 1385.58) | 34.11 (46.95 to 24.26) | 0.5 (0.47 to 0.53) | 958.99 (1193.49 to 766.49) | 16.97 (21.08 to 13.58) | 0.67 (0.58 to 0.77) | 15.55 (19.86 to 12.20) | 0.28 (0.36 to 0.22) | 0.76 (0.67 to 0.86) |
| Kiribati | 0.97 (1.35 to 0.66) | 6.02 (8.36 to 4.12) | -0.01 (-0.08 to 0.06) | 5.80 (8.09 to 3.99) | 36.22 (50.29 to 25.01) | 0.07 (0.04 to 0.11) | 3.91 (5.17 to 2.94) | 24.71 (32.68 to 18.57) | -0.26 (-0.32 to -0.21) | 0.07 (0.10 to 0.05) | 0.47 (0.67 to 0.33) | -0.36 (-0.43 to -0.28) |
| Kuwait | 60.24 (88.72 to 34.54) | 5.31 (7.75 to 3.07) | 0.38 (0.27 to 0.48) | 436.42 (632.30 to 283.37) | 38.69 (55.68 to 25.22) | 0.54 (0.5 to 0.57) | 161.59 (215.08 to 120.34) | 14.47 (19.18 to 10.80) | -2.34 (-3.62 to -1.04) | 2.23 (2.78 to 1.78) | 0.20 (0.25 to 0.16) | -3.55 (-5.81 to -1.23) |
| Kyrgyzstan | 37.23 (58.35 to 19.90) | 3.63 (5.70 to 1.94) | -0.3 (-0.35 to -0.25) | 262.82 (396.99 to 162.40) | 25.65 (38.75 to 15.85) | -0.24 (-0.29 to -0.2) | 125.34 (159.94 to 97.78) | 12.23 (15.61 to 9.54) | -0.86 (-1.39 to -0.31) | 2.02 (2.47 to 1.63) | 0.20 (0.24 to 0.16) | -1.07 (-1.6 to -0.53) |
| Lao People's Democratic Republic | 49.62 (74.63 to 29.33) | 4.62 (6.93 to 2.74) | 0.04 (-0.06 to 0.13) | 361.86 (518.89 to 241.77) | 33.68 (48.28 to 22.53) | 0.03 (0 to 0.06) | 256.98 (333.96 to 190.61) | 23.92 (31.07 to 17.74) | -0.77 (-0.82 to -0.72) | 4.78 (6.49 to 3.41) | 0.45 (0.61 to 0.32) | -0.99 (-1.04 to -0.94) |
| Latvia | 20.99 (31.52 to 12.58) | 5.26 (7.92 to 3.13) | -0.12 (-0.2 to -0.04) | 134.54 (195.65 to 88.76) | 33.60 (49.04 to 22.07) | -0.16 (-0.21 to -0.11) | 56.08 (71.17 to 44.24) | 13.92 (17.70 to 10.97) | -0.34 (-1.22 to 0.56) | 0.85 (0.97 to 0.74) | 0.21 (0.24 to 0.18) | -0.51 (-1.76 to 0.76) |
| Lebanon | 49.64 (73.18 to 29.82) | 5.27 (7.76 to 3.17) | 0.84 (0.77 to 0.91) | 360.82 (518.49 to 240.44) | 38.44 (55.14 to 25.67) | 0.77 (0.74 to 0.81) | 155.95 (199.42 to 118.39) | 16.65 (21.27 to 12.65) | -1.48 (-1.69 to -1.26) | 2.38 (2.96 to 1.81) | 0.26 (0.32 to 0.19) | -2.36 (-2.55 to -2.16) |
| Lesotho | 8.40 (12.48 to 4.89) | 3.81 (5.64 to 2.21) | 0.76 (0.71 to 0.81) | 68.01 (99.15 to 45.02) | 31.01 (44.90 to 20.62) | 0.58 (0.54 to 0.61) | 55.44 (76.23 to 37.29) | 25.66 (35.31 to 17.27) | 1.75 (1.56 to 1.94) | 1.08 (1.57 to 0.69) | 0.51 (0.74 to 0.32) | 2.07 (1.75 to 2.4) |
| Liberia | 19.90 (32.45 to 9.97) | 3.13 (5.03 to 1.60) | 0.42 (0.39 to 0.45) | 181.04 (266.40 to 116.16) | 28.33 (41.60 to 18.26) | 0.46 (0.37 to 0.54) | 134.67 (218.72 to 85.19) | 19.43 (30.98 to 12.47) | 0.3 (0.12 to 0.48) | 2.36 (4.12 to 1.32) | 0.34 (0.58 to 0.19) | 0.21 (0 to 0.43) |
| Libya | 95.71 (136.38 to 60.47) | 6.36 (9.04 to 4.03) | 1.18 (1.03 to 1.33) | 683.17 (949.59 to 468.82) | 45.54 (63.16 to 31.34) | 1.21 (1.07 to 1.35) | 519.37 (827.30 to 337.64) | 34.82 (55.56 to 22.57) | 1.61 (1.24 to 1.97) | 10.05 (17.63 to 5.96) | 0.68 (1.19 to 0.40) | 1.71 (1.11 to 2.31) |
| Lithuania | 32.16 (47.99 to 18.57) | 5.37 (8.08 to 3.08) | 0.06 (0 to 0.12) | 204.37 (301.77 to 132.74) | 34.12 (50.70 to 22.05) | -0.04 (-0.08 to 0) | 80.79 (104.95 to 62.97) | 13.42 (17.50 to 10.43) | -0.21 (-0.74 to 0.32) | 1.18 (1.35 to 1.03) | 0.19 (0.22 to 0.17) | -0.3 (-1.27 to 0.68) |
| Luxembourg | 9.13 (13.84 to 5.30) | 6.07 (9.24 to 3.49) | 0.53 (0.5 to 0.55) | 71.03 (104.87 to 46.02) | 47.14 (69.63 to 30.50) | 0.49 (0.45 to 0.52) | 20.85 (29.61 to 14.56) | 13.74 (19.58 to 9.57) | -0.75 (-0.96 to -0.54) | 0.24 (0.27 to 0.22) | 0.16 (0.17 to 0.14) | -1.75 (-2.18 to -1.31) |
| Madagascar | 105.47 (164.41 to 58.68) | 3.33 (5.17 to 1.86) | 0.44 (0.41 to 0.46) | 921.40 (1346.00 to 611.26) | 29.21 (42.60 to 19.43) | 0.41 (0.38 to 0.44) | 466.64 (632.79 to 334.83) | 14.94 (20.24 to 10.72) | -0.18 (-0.37 to 0.02) | 7.70 (10.76 to 5.20) | 0.25 (0.35 to 0.17) | -0.43 (-0.53 to -0.34) |
| Malawi | 66.96 (104.17 to 36.92) | 3.60 (5.56 to 1.99) | 0.28 (0.21 to 0.34) | 575.32 (822.19 to 375.89) | 31.06 (44.18 to 20.41) | 0.25 (0.22 to 0.29) | 341.70 (454.32 to 238.44) | 18.78 (24.93 to 13.10) | 0.31 (0.21 to 0.41) | 6.00 (8.10 to 4.01) | 0.34 (0.45 to 0.23) | 0.34 (0.2 to 0.48) |
| Malaysia | 259.53 (394.67 to 144.80) | 4.89 (7.43 to 2.73) | 0.62 (0.51 to 0.72) | 1941.00 (2800.48 to 1258.10) | 36.57 (52.71 to 23.73) | 0.55 (0.5 to 0.6) | 1109.01 (1370.85 to 894.29) | 20.90 (25.84 to 16.85) | 0.59 (0.28 to 0.9) | 19.22 (22.79 to 15.57) | 0.36 (0.43 to 0.29) | 0.74 (0.56 to 0.93) |
| Maldives | 4.26 (6.81 to 2.35) | 4.52 (7.18 to 2.51) | -0.13 (-0.21 to -0.06) | 32.14 (47.45 to 20.30) | 34.15 (50.16 to 21.69) | -0.07 (-0.13 to -0.01) | 12.98 (18.41 to 9.36) | 13.74 (19.42 to 9.90) | -1.81 (-1.94 to -1.68) | 0.18 (0.27 to 0.13) | 0.20 (0.29 to 0.14) | -2.62 (-2.86 to -2.38) |
| Mali | 59.65 (95.91 to 30.88) | 2.84 (4.54 to 1.47) | 0.21 (0.12 to 0.3) | 537.53 (792.07 to 340.39) | 25.56 (37.62 to 16.22) | 0.2 (0.16 to 0.24) | 405.21 (576.29 to 282.69) | 18.69 (26.45 to 13.08) | -0.05 (-0.15 to 0.05) | 7.27 (10.64 to 4.90) | 0.34 (0.49 to 0.23) | -0.15 (-0.26 to -0.03) |
| Malta | 5.26 (8.46 to 3.02) | 6.03 (9.70 to 3.47) | 0.72 (0.65 to 0.79) | 41.13 (60.43 to 27.14) | 47.22 (69.42 to 31.14) | 0.66 (0.62 to 0.71) | 13.62 (18.82 to 9.95) | 15.62 (21.59 to 11.40) | 0.39 (0.03 to 0.76) | 0.17 (0.20 to 0.15) | 0.20 (0.22 to 0.18) | 0.18 (-0.43 to 0.79) |
| Marshall Islands | 0.59 (0.83 to 0.40) | 6.70 (9.38 to 4.49) | 0.38 (0.29 to 0.48) | 3.68 (5.17 to 2.57) | 41.73 (58.52 to 29.24) | 0.41 (0.33 to 0.49) | 2.68 (3.57 to 1.95) | 30.79 (41.02 to 22.45) | -0.11 (-0.14 to -0.08) | 0.05 (0.07 to 0.04) | 0.60 (0.85 to 0.42) | -0.24 (-0.28 to -0.2) |
| Mauritania | 13.80 (22.07 to 7.41) | 2.98 (4.76 to 1.61) | 0.17 (0.11 to 0.23) | 119.67 (175.80 to 77.40) | 25.86 (37.94 to 16.76) | 0.18 (0.15 to 0.21) | 66.30 (106.55 to 43.01) | 13.99 (22.34 to 9.10) | -0.43 (-0.57 to -0.29) | 1.08 (1.94 to 0.64) | 0.23 (0.41 to 0.14) | -0.67 (-1.04 to -0.29) |
| Mauritius | 14.91 (21.77 to 8.85) | 5.40 (7.91 to 3.19) | 0.42 (0.36 to 0.48) | 109.85 (159.18 to 73.60) | 39.74 (57.64 to 26.61) | 0.36 (0.33 to 0.4) | 61.71 (74.79 to 51.40) | 22.39 (27.14 to 18.64) | 0.01 (-0.55 to 0.57) | 1.07 (1.19 to 0.94) | 0.39 (0.43 to 0.34) | -0.13 (-0.85 to 0.59) |
| Mexico | 1822.80 (2604.61 to 1162.32) | 7.52 (10.73 to 4.80) | 0.87 (0.62 to 1.12) | 16064.91 (21495.60 to 11511.42) | 66.20 (88.61 to 47.41) | 0.81 (0.7 to 0.91) | 6050.02 (7397.50 to 4940.09) | 24.92 (30.47 to 20.34) | 0.28 (0.14 to 0.43) | 86.55 (98.91 to 75.06) | 0.36 (0.41 to 0.31) | -0.03 (-0.31 to 0.26) |
| Micronesia (Federated States of) | 1.09 (1.53 to 0.76) | 6.61 (9.23 to 4.58) | 0.59 (0.49 to 0.69) | 6.61 (9.24 to 4.68) | 39.92 (55.87 to 28.24) | 0.52 (0.48 to 0.57) | 4.54 (5.96 to 3.33) | 27.37 (35.92 to 20.05) | -0.38 (-0.43 to -0.33) | 0.09 (0.12 to 0.06) | 0.52 (0.72 to 0.37) | -0.57 (-0.63 to -0.51) |
| Monaco | 0.57 (0.85 to 0.34) | 6.45 (9.77 to 3.74) | 0.57 (0.54 to 0.61) | 4.37 (6.27 to 2.84) | 48.98 (70.63 to 31.84) | 0.53 (0.47 to 0.59) | 1.64 (2.27 to 1.13) | 18.18 (25.40 to 12.46) | 0.27 (0.18 to 0.37) | 0.02 (0.03 to 0.02) | 0.26 (0.36 to 0.17) | 0.12 (0.05 to 0.19) |
| Mongolia | 21.70 (33.50 to 12.40) | 3.71 (5.72 to 2.13) | -0.26 (-0.38 to -0.14) | 147.75 (217.10 to 92.98) | 25.25 (37.09 to 15.91) | -0.18 (-0.22 to -0.15) | 81.82 (100.70 to 64.47) | 13.98 (17.21 to 11.01) | -0.23 (-0.5 to 0.05) | 1.40 (1.68 to 1.11) | 0.24 (0.29 to 0.19) | -0.23 (-0.62 to 0.17) |
| Montenegro | 5.60 (8.42 to 3.06) | 4.29 (6.46 to 2.33) | -0.02 (-0.06 to 0.02) | 34.62 (50.12 to 21.40) | 26.55 (38.51 to 16.38) | -0.01 (-0.04 to 0.03) | 17.19 (21.93 to 13.56) | 13.17 (16.81 to 10.39) | -0.34 (-0.67 to -0.01) | 0.29 (0.36 to 0.23) | 0.22 (0.27 to 0.18) | -0.49 (-0.92 to -0.05) |
| Morocco | 450.97 (630.06 to 306.27) | 6.77 (9.46 to 4.60) | 1.46 (1.38 to 1.55) | 3206.10 (4360.63 to 2232.22) | 48.20 (65.53 to 33.58) | 1.4 (1.32 to 1.48) | 2037.31 (2762.35 to 1449.68) | 30.68 (41.60 to 21.83) | 0.72 (0.64 to 0.81) | 37.95 (55.81 to 24.90) | 0.57 (0.84 to 0.38) | 0.55 (0.46 to 0.64) |
| Mozambique | 101.62 (156.56 to 53.58) | 3.54 (5.43 to 1.87) | 0.51 (0.49 to 0.54) | 868.45 (1238.98 to 575.29) | 30.32 (43.19 to 20.13) | 0.42 (0.4 to 0.44) | 604.37 (837.67 to 420.02) | 21.34 (29.57 to 14.86) | 0.79 (0.72 to 0.86) | 11.16 (16.33 to 7.25) | 0.40 (0.58 to 0.26) | 0.92 (0.82 to 1.03) |
| Myanmar | 476.16 (718.53 to 283.77) | 4.80 (7.25 to 2.86) | 0.41 (0.34 to 0.48) | 3433.93 (4948.72 to 2259.00) | 34.62 (49.90 to 22.77) | 0.39 (0.34 to 0.43) | 2362.12 (3085.17 to 1731.44) | 23.82 (31.11 to 17.46) | -0.3 (-0.36 to -0.25) | 43.80 (59.88 to 31.16) | 0.44 (0.60 to 0.31) | -0.47 (-0.54 to -0.4) |
| Namibia | 12.57 (19.28 to 7.25) | 3.87 (5.91 to 2.23) | 0.35 (0.3 to 0.39) | 105.65 (151.86 to 70.29) | 32.61 (46.76 to 21.76) | 0.32 (0.27 to 0.36) | 77.22 (105.46 to 55.41) | 24.09 (32.92 to 17.30) | 0.53 (0.36 to 0.71) | 1.46 (2.05 to 0.98) | 0.46 (0.65 to 0.31) | 0.58 (0.39 to 0.77) |
| Nauru | 0.10 (0.14 to 0.06) | 6.99 (9.70 to 4.67) | 0.34 (0.26 to 0.43) | 0.59 (0.82 to 0.41) | 42.57 (58.80 to 29.38) | 0.3 (0.25 to 0.35) | 0.49 (0.69 to 0.32) | 36.19 (50.81 to 23.61) | -0.45 (-0.53 to -0.37) | 0.01 (0.01 to 0.01) | 0.74 (1.08 to 0.44) | -0.59 (-0.68 to -0.5) |
| Nepal | 229.39 (343.69 to 133.52) | 5.11 (7.65 to 2.98) | 1.05 (0.99 to 1.12) | 1842.30 (2702.33 to 1196.87) | 41.13 (60.26 to 26.76) | 0.96 (0.92 to 0.99) | 869.60 (1158.18 to 633.97) | 19.45 (25.90 to 14.18) | 0.33 (0.28 to 0.38) | 14.06 (19.02 to 10.10) | 0.32 (0.43 to 0.23) | 0.1 (0.02 to 0.18) |
| Netherlands | 243.43 (363.23 to 145.53) | 6.55 (9.88 to 3.88) | 0.41 (0.33 to 0.49) | 1858.31 (2674.72 to 1228.63) | 49.84 (72.16 to 32.87) | 0.47 (0.43 to 0.52) | 551.60 (781.11 to 389.25) | 14.62 (20.82 to 10.27) | -0.05 (-0.15 to 0.06) | 6.41 (6.82 to 5.97) | 0.17 (0.18 to 0.15) | -0.54 (-0.8 to -0.28) |
| New Zealand | 25.70 (38.23 to 14.87) | 2.52 (3.77 to 1.44) | 0.57 (0.51 to 0.64) | 182.71 (260.23 to 121.25) | 18.06 (25.83 to 11.95) | 0.78 (0.7 to 0.85) | 142.37 (162.50 to 126.54) | 13.84 (15.82 to 12.27) | 0.29 (0.17 to 0.42) | 2.79 (2.99 to 2.63) | 0.27 (0.29 to 0.25) | 0.21 (0.05 to 0.37) |
| Nicaragua | 76.90 (112.76 to 48.06) | 7.64 (11.19 to 4.77) | 0.78 (0.66 to 0.89) | 633.84 (895.48 to 431.63) | 63.48 (89.43 to 43.38) | 0.65 (0.57 to 0.73) | 199.83 (274.14 to 146.41) | 20.04 (27.46 to 14.70) | 0.02 (-0.15 to 0.19) | 2.48 (3.13 to 1.93) | 0.25 (0.32 to 0.20) | -0.44 (-0.81 to -0.08) |
| Niger | 53.27 (85.20 to 27.53) | 2.83 (4.50 to 1.47) | 0.2 (0.16 to 0.23) | 477.21 (714.26 to 303.90) | 25.31 (37.70 to 16.19) | 0.17 (0.1 to 0.25) | 321.80 (478.21 to 215.46) | 16.36 (24.18 to 10.99) | -0.05 (-0.33 to 0.23) | 5.54 (8.62 to 3.45) | 0.28 (0.43 to 0.18) | -0.1 (-0.37 to 0.17) |
| Nigeria | 766.46 (1148.38 to 428.28) | 3.19 (4.77 to 1.79) | 0.38 (0.34 to 0.42) | 6935.35 (9666.70 to 4763.07) | 28.84 (40.16 to 19.82) | 0.3 (0.26 to 0.34) | 4377.01 (6808.53 to 2989.70) | 17.78 (27.51 to 12.19) | -0.23 (-0.37 to -0.09) | 74.18 (124.15 to 45.19) | 0.30 (0.50 to 0.18) | -0.37 (-0.58 to -0.15) |
| Niue | 0.02 (0.03 to 0.01) | 6.21 (8.99 to 3.90) | 0.29 (0.07 to 0.51) | 0.14 (0.20 to 0.09) | 39.99 (57.31 to 26.50) | 0.3 (0.2 to 0.4) | 0.08 (0.11 to 0.06) | 22.96 (31.20 to 16.88) | -0.49 (-0.67 to -0.31) | 0.00 (0.00 to 0.00) | 0.42 (0.58 to 0.29) | -0.73 (-0.95 to -0.5) |
| North Macedonia | 21.92 (32.92 to 12.79) | 4.35 (6.54 to 2.53) | 0.17 (0.13 to 0.22) | 134.60 (198.42 to 85.00) | 26.68 (39.39 to 16.84) | 0.12 (0.11 to 0.13) | 58.73 (78.27 to 44.37) | 11.62 (15.47 to 8.78) | -0.45 (-0.65 to -0.26) | 0.92 (1.22 to 0.67) | 0.18 (0.24 to 0.13) | -0.69 (-0.98 to -0.4) |
| Northern Mariana Islands | 0.69 (1.00 to 0.43) | 5.80 (8.44 to 3.60) | 0.18 (0.07 to 0.29) | 4.60 (6.59 to 2.98) | 38.66 (55.65 to 25.02) | 0.16 (0.02 to 0.3) | 2.40 (3.17 to 1.87) | 19.82 (26.17 to 15.42) | -0.4 (-0.48 to -0.32) | 0.04 (0.06 to 0.03) | 0.34 (0.45 to 0.26) | -0.65 (-0.89 to -0.42) |
| Norway | 55.70 (80.91 to 33.79) | 4.81 (7.02 to 2.90) | 3.51 (3.13 to 3.89) | 462.34 (634.60 to 324.63) | 39.95 (54.97 to 28.04) | 2.48 (2.24 to 2.72) | 170.54 (211.25 to 139.19) | 14.49 (18.04 to 11.76) | 0.61 (0.22 to 1) | 2.43 (2.53 to 2.32) | 0.20 (0.21 to 0.19) | -0.31 (-1.11 to 0.51) |
| Oman | 45.19 (69.60 to 27.32) | 5.93 (9.06 to 3.58) | 0.89 (0.78 to 0.99) | 321.78 (483.52 to 214.29) | 43.12 (64.24 to 28.96) | 0.93 (0.85 to 1.02) | 154.34 (202.99 to 115.58) | 21.29 (28.05 to 16.00) | -1 (-1.26 to -0.73) | 2.48 (3.38 to 1.82) | 0.35 (0.48 to 0.26) | -1.65 (-2.03 to -1.26) |
| Pakistan | 1612.72 (2367.53 to 997.77) | 5.31 (7.78 to 3.29) | 0.46 (0.3 to 0.62) | 13494.20 (18643.74 to 9484.46) | 44.60 (61.47 to 31.45) | 0.5 (0.4 to 0.6) | 6820.13 (8857.10 to 5116.42) | 22.70 (29.46 to 17.03) | 0.58 (0.52 to 0.63) | 113.06 (148.93 to 84.84) | 0.38 (0.50 to 0.29) | 0.62 (0.52 to 0.71) |
| Palau | 0.34 (0.49 to 0.22) | 6.80 (9.83 to 4.32) | 0.11 (-0.02 to 0.25) | 2.24 (3.18 to 1.49) | 44.52 (63.38 to 29.70) | 0.24 (0.2 to 0.29) | 1.05 (1.37 to 0.76) | 20.81 (27.16 to 15.01) | -0.47 (-0.54 to -0.4) | 0.02 (0.02 to 0.01) | 0.34 (0.46 to 0.25) | -0.79 (-0.89 to -0.69) |
| Palestine | 35.77 (52.76 to 22.01) | 5.57 (8.21 to 3.43) | 0.85 (0.76 to 0.93) | 255.69 (366.56 to 172.16) | 39.95 (57.17 to 26.95) | 0.83 (0.79 to 0.86) | 112.58 (143.01 to 86.93) | 17.67 (22.40 to 13.66) | -0.56 (-0.78 to -0.35) | 1.76 (2.16 to 1.38) | 0.28 (0.34 to 0.22) | -1.15 (-1.45 to -0.84) |
| Panama | 63.99 (94.88 to 36.93) | 8.50 (12.61 to 4.90) | 0.93 (0.63 to 1.23) | 537.99 (768.58 to 358.95) | 71.10 (101.73 to 47.38) | 0.8 (0.65 to 0.95) | 163.60 (232.20 to 114.21) | 21.60 (30.68 to 15.06) | 0.44 (0.04 to 0.85) | 1.97 (2.42 to 1.52) | 0.26 (0.32 to 0.20) | 0.16 (-0.55 to 0.87) |
| Papua New Guinea | 70.65 (102.88 to 43.70) | 5.09 (7.39 to 3.17) | 0.44 (0.39 to 0.48) | 462.52 (666.33 to 306.00) | 33.45 (48.05 to 22.15) | 0.4 (0.34 to 0.45) | 286.64 (425.48 to 196.70) | 21.11 (31.40 to 14.47) | -0.28 (-0.35 to -0.22) | 5.26 (8.59 to 3.33) | 0.39 (0.64 to 0.25) | -0.5 (-0.6 to -0.41) |
| Paraguay | 81.55 (119.24 to 49.61) | 7.47 (10.92 to 4.53) | 1.07 (1.01 to 1.12) | 676.70 (960.01 to 457.71) | 62.34 (88.33 to 42.26) | 0.87 (0.74 to 1) | 220.72 (308.66 to 157.05) | 20.39 (28.49 to 14.53) | 0.97 (0.8 to 1.14) | 2.82 (3.76 to 2.01) | 0.26 (0.35 to 0.19) | 1.21 (1.06 to 1.36) |
| Peru | 1028.62 (1446.60 to 672.32) | 16.49 (23.20 to 10.78) | 1.78 (1.64 to 1.92) | 8140.47 (11058.99 to 5704.89) | 131.16 (178.05 to 92.01) | 1.3 (1.23 to 1.37) | 2057.15 (2897.87 to 1433.17) | 33.16 (46.68 to 23.12) | 0.55 (0.38 to 0.73) | 19.46 (26.53 to 14.07) | 0.31 (0.43 to 0.23) | -0.22 (-0.65 to 0.22) |
| Philippines | 832.61 (1200.75 to 522.62) | 4.65 (6.71 to 2.91) | 0.46 (0.43 to 0.49) | 6575.52 (9076.81 to 4649.27) | 36.73 (50.66 to 25.99) | 0.41 (0.38 to 0.45) | 4546.59 (5429.84 to 3672.01) | 25.40 (30.33 to 20.51) | 0.69 (0.56 to 0.83) | 83.87 (100.94 to 66.47) | 0.47 (0.56 to 0.37) | 0.8 (0.61 to 0.99) |
| Poland | 355.52 (455.86 to 263.81) | 4.44 (5.68 to 3.30) | -0.08 (-0.13 to -0.04) | 2055.15 (2576.06 to 1619.06) | 25.67 (32.16 to 20.23) | -0.91 (-0.96 to -0.86) | 1078.68 (1230.32 to 939.85) | 13.48 (15.37 to 11.74) | -0.72 (-0.79 to -0.64) | 18.40 (20.09 to 16.77) | 0.23 (0.25 to 0.21) | -0.51 (-0.72 to -0.3) |
| Portugal | 133.92 (201.43 to 78.90) | 5.38 (8.11 to 3.17) | 0.92 (0.88 to 0.96) | 1092.73 (1557.73 to 731.46) | 43.83 (62.70 to 29.25) | 0.77 (0.73 to 0.82) | 354.46 (503.10 to 262.10) | 14.11 (20.09 to 10.40) | 0.21 (0.06 to 0.36) | 4.47 (4.78 to 4.15) | 0.18 (0.19 to 0.16) | -0.34 (-0.66 to -0.03) |
| Puerto Rico | 45.51 (68.11 to 27.03) | 6.80 (10.19 to 4.05) | 0.59 (0.5 to 0.68) | 398.05 (564.57 to 268.96) | 58.80 (83.68 to 39.59) | 0.47 (0.42 to 0.52) | 148.59 (194.32 to 110.55) | 21.82 (28.63 to 16.21) | 0.45 (0.26 to 0.63) | 2.12 (2.59 to 1.69) | 0.31 (0.38 to 0.24) | 0.52 (0.11 to 0.93) |
| Qatar | 35.40 (53.73 to 20.71) | 5.93 (8.92 to 3.52) | 0.43 (0.35 to 0.5) | 251.55 (374.20 to 161.29) | 42.75 (62.70 to 27.65) | 0.57 (0.53 to 0.6) | 104.15 (146.24 to 72.54) | 18.04 (25.39 to 12.56) | -1.24 (-1.62 to -0.87) | 1.53 (2.29 to 1.04) | 0.27 (0.41 to 0.19) | -2.01 (-2.49 to -1.53) |
| Republic of Korea | 656.59 (959.72 to 389.65) | 4.77 (7.03 to 2.79) | 1.18 (1.1 to 1.25) | 4376.40 (6311.15 to 2788.58) | 32.01 (46.38 to 20.30) | 1.07 (1.01 to 1.12) | 2066.80 (2642.11 to 1657.10) | 15.02 (19.21 to 12.01) | -0.92 (-1.02 to -0.83) | 33.75 (40.10 to 28.34) | 0.24 (0.29 to 0.20) | -1.6 (-1.74 to -1.46) |
| Republic of Moldova | 37.13 (55.74 to 20.66) | 4.81 (7.21 to 2.69) | -0.24 (-0.36 to -0.13) | 246.51 (360.54 to 153.23) | 31.93 (46.70 to 19.85) | -0.28 (-0.33 to -0.23) | 96.45 (128.56 to 74.89) | 12.50 (16.67 to 9.71) | -0.43 (-1.05 to 0.19) | 1.39 (1.57 to 1.22) | 0.18 (0.20 to 0.16) | -0.52 (-1.47 to 0.44) |
| Romania | 195.25 (293.87 to 111.20) | 4.01 (6.09 to 2.26) | 0.33 (0.29 to 0.37) | 1268.93 (1869.84 to 810.19) | 26.10 (38.62 to 16.62) | 0.32 (0.27 to 0.37) | 754.26 (899.76 to 626.87) | 15.42 (18.40 to 12.81) | 0.08 (-0.48 to 0.63) | 13.62 (15.50 to 11.83) | 0.28 (0.31 to 0.24) | -0.01 (-0.76 to 0.75) |
| Russian Federation | 1539.93 (2203.64 to 960.82) | 5.16 (7.38 to 3.22) | 0.03 (-0.07 to 0.14) | 10309.79 (14436.45 to 7028.08) | 34.53 (48.36 to 23.53) | 0.01 (-0.04 to 0.06) | 4515.64 (5458.65 to 3819.29) | 15.12 (18.28 to 12.79) | 0.14 (-0.54 to 0.82) | 70.00 (75.84 to 64.22) | 0.23 (0.25 to 0.21) | 0.21 (-0.74 to 1.16) |
| Rwanda | 52.03 (80.43 to 28.72) | 3.48 (5.36 to 1.93) | -0.27 (-0.3 to -0.24) | 445.39 (654.05 to 289.44) | 29.96 (43.87 to 19.56) | -0.14 (-0.17 to -0.12) | 265.90 (373.21 to 177.52) | 18.18 (25.50 to 12.13) | -1.39 (-1.62 to -1.16) | 4.69 (7.01 to 2.89) | 0.33 (0.49 to 0.20) | -1.72 (-1.98 to -1.45) |
| Saint Kitts and Nevis | 0.93 (1.36 to 0.60) | 6.88 (10.07 to 4.40) | 0.54 (0.42 to 0.65) | 8.06 (11.25 to 5.52) | 58.90 (82.63 to 40.21) | 0.4 (0.32 to 0.48) | 3.85 (4.99 to 2.96) | 27.83 (36.22 to 21.40) | -0.54 (-0.84 to -0.24) | 0.06 (0.08 to 0.05) | 0.46 (0.57 to 0.35) | -0.82 (-1 to -0.64) |
| Saint Lucia | 2.77 (4.16 to 1.71) | 6.79 (10.22 to 4.19) | 0.5 (0.26 to 0.73) | 24.05 (33.95 to 16.67) | 58.17 (82.20 to 40.19) | 0.39 (0.27 to 0.51) | 10.24 (13.26 to 7.76) | 24.65 (31.98 to 18.67) | -0.07 (-0.32 to 0.18) | 0.16 (0.20 to 0.13) | 0.38 (0.47 to 0.30) | -0.28 (-0.56 to 0.01) |
| Saint Vincent and the Grenadines | 1.38 (2.03 to 0.83) | 6.06 (8.91 to 3.65) | 0.54 (0.4 to 0.68) | 12.38 (17.44 to 8.25) | 53.81 (76.00 to 35.78) | 0.42 (0.32 to 0.51) | 5.82 (7.32 to 4.67) | 25.18 (31.72 to 20.19) | 0.69 (0.5 to 0.88) | 0.09 (0.11 to 0.08) | 0.41 (0.47 to 0.35) | 0.82 (0.55 to 1.1) |
| Samoa | 1.68 (2.44 to 1.06) | 5.76 (8.39 to 3.65) | 0.43 (0.36 to 0.5) | 10.86 (15.82 to 7.26) | 37.25 (54.29 to 24.89) | 0.45 (0.42 to 0.48) | 6.38 (8.86 to 4.63) | 21.81 (30.27 to 15.80) | -0.07 (-0.15 to 0.01) | 0.12 (0.17 to 0.08) | 0.39 (0.58 to 0.26) | -0.24 (-0.34 to -0.15) |
| San Marino | 0.45 (0.69 to 0.26) | 5.48 (8.41 to 3.07) | 0.2 (0.14 to 0.27) | 3.60 (5.22 to 2.32) | 43.68 (63.60 to 28.07) | 0.27 (0.24 to 0.29) | 0.79 (1.22 to 0.45) | 9.56 (14.85 to 5.42) | -0.45 (-0.55 to -0.35) | 0.01 (0.01 to 0.00) | 0.07 (0.10 to 0.04) | -1.84 (-1.98 to -1.7) |
| Sao Tome and Principe | 1.05 (1.66 to 0.56) | 3.67 (5.78 to 1.95) | 0.74 (0.69 to 0.8) | 9.95 (14.30 to 6.66) | 34.85 (49.99 to 23.36) | 0.72 (0.67 to 0.76) | 5.79 (8.31 to 4.03) | 19.80 (28.16 to 13.80) | 0.52 (0.34 to 0.71) | 0.10 (0.14 to 0.07) | 0.33 (0.48 to 0.23) | 0.44 (0.22 to 0.67) |
| Saudi Arabia | 540.56 (805.23 to 344.60) | 6.91 (10.23 to 4.42) | 1.54 (1.48 to 1.6) | 3781.49 (5428.19 to 2581.46) | 49.11 (69.94 to 33.79) | 1.48 (1.45 to 1.52) | 3436.66 (4506.99 to 2597.43) | 44.97 (58.74 to 34.15) | 0.36 (0.28 to 0.43) | 68.09 (91.23 to 49.79) | 0.91 (1.21 to 0.67) | 0.16 (0.08 to 0.25) |
| Senegal | 54.42 (85.86 to 28.21) | 3.19 (5.01 to 1.66) | 0.55 (0.54 to 0.57) | 481.99 (700.55 to 307.85) | 28.22 (40.99 to 18.06) | 0.57 (0.52 to 0.62) | 348.55 (528.19 to 234.66) | 19.78 (29.79 to 13.35) | 0.51 (0.21 to 0.81) | 6.15 (9.92 to 3.95) | 0.35 (0.56 to 0.22) | 0.49 (0.09 to 0.89) |
| Serbia | 73.09 (110.50 to 41.36) | 3.98 (6.02 to 2.25) | 0 (-0.04 to 0.03) | 444.67 (648.76 to 275.83) | 24.22 (35.36 to 15.01) | 0.01 (-0.05 to 0.06) | 214.32 (277.31 to 166.98) | 11.66 (15.09 to 9.08) | -0.43 (-0.89 to 0.02) | 3.52 (4.32 to 2.83) | 0.19 (0.23 to 0.15) | -0.59 (-1.16 to -0.03) |
| Seychelles | 1.40 (2.08 to 0.83) | 6.00 (8.91 to 3.56) | 0.42 (0.33 to 0.51) | 10.08 (14.39 to 6.70) | 43.13 (61.62 to 28.66) | 0.4 (0.37 to 0.42) | 7.32 (9.15 to 5.91) | 31.49 (39.35 to 25.45) | -0.76 (-1.16 to -0.37) | 0.14 (0.17 to 0.11) | 0.59 (0.73 to 0.48) | -1.04 (-1.58 to -0.5) |
| Sierra Leone | 27.19 (43.39 to 14.32) | 2.99 (4.74 to 1.59) | 0.51 (0.47 to 0.56) | 239.12 (352.90 to 151.81) | 26.27 (38.74 to 16.73) | 0.48 (0.45 to 0.5) | 167.46 (249.93 to 110.30) | 17.47 (25.84 to 11.54) | 0.4 (0.23 to 0.57) | 2.92 (4.60 to 1.81) | 0.30 (0.47 to 0.19) | 0.37 (0.16 to 0.58) |
| Singapore | 61.44 (92.18 to 34.50) | 4.31 (6.48 to 2.41) | 0.48 (0.33 to 0.62) | 443.36 (648.04 to 286.04) | 31.19 (45.44 to 20.18) | 0.55 (0.42 to 0.67) | 159.81 (213.14 to 122.03) | 11.36 (15.13 to 8.70) | -0.95 (-1.16 to -0.74) | 2.18 (2.35 to 2.01) | 0.16 (0.17 to 0.14) | -1.72 (-1.99 to -1.45) |
| Slovakia | 46.88 (71.27 to 26.64) | 3.89 (5.89 to 2.22) | 0 (-0.09 to 0.08) | 293.33 (429.26 to 180.63) | 24.31 (35.59 to 14.98) | 0.02 (-0.06 to 0.09) | 138.04 (177.49 to 109.14) | 11.46 (14.74 to 9.06) | -1.04 (-1.24 to -0.84) | 2.22 (2.74 to 1.80) | 0.19 (0.23 to 0.15) | -1.45 (-1.74 to -1.16) |
| Slovenia | 20.58 (31.27 to 11.46) | 4.35 (6.64 to 2.40) | -0.04 (-0.07 to -0.02) | 129.08 (194.36 to 78.61) | 27.25 (41.10 to 16.56) | 0.04 (0.01 to 0.07) | 47.28 (62.89 to 36.29) | 9.95 (13.25 to 7.63) | -1.28 (-1.5 to -1.07) | 0.66 (0.77 to 0.55) | 0.14 (0.16 to 0.11) | -1.93 (-2.24 to -1.63) |
| Solomon Islands | 6.31 (8.90 to 4.31) | 6.88 (9.66 to 4.73) | 0.42 (0.34 to 0.51) | 37.86 (52.60 to 26.31) | 41.54 (57.55 to 28.94) | 0.41 (0.31 to 0.5) | 25.03 (33.64 to 18.47) | 27.88 (37.45 to 20.56) | -0.07 (-0.21 to 0.07) | 0.47 (0.65 to 0.33) | 0.53 (0.74 to 0.37) | -0.23 (-0.52 to 0.05) |
| Somalia | 64.91 (103.92 to 35.29) | 3.56 (5.64 to 1.95) | 0.1 (0.06 to 0.14) | 545.57 (803.83 to 350.68) | 30.19 (44.24 to 19.63) | 0.11 (0.09 to 0.13) | 326.78 (475.44 to 219.59) | 18.97 (27.54 to 12.76) | -0.3 (-0.36 to -0.24) | 5.65 (9.02 to 3.38) | 0.34 (0.54 to 0.21) | -0.45 (-0.55 to -0.34) |
| South Africa | 334.73 (498.11 to 194.22) | 3.60 (5.35 to 2.09) | 0.21 (0.16 to 0.26) | 2968.24 (4125.47 to 2082.88) | 31.98 (44.37 to 22.46) | 0.19 (0.17 to 0.22) | 1877.35 (2294.63 to 1548.07) | 20.33 (24.84 to 16.78) | 0.51 (0.35 to 0.67) | 33.91 (41.41 to 28.05) | 0.37 (0.45 to 0.31) | 0.65 (0.4 to 0.89) |
| South Sudan | 35.92 (56.20 to 18.45) | 3.20 (4.99 to 1.65) | 0.13 (0.09 to 0.17) | 316.75 (458.87 to 205.33) | 28.31 (40.92 to 18.40) | 0.12 (0.1 to 0.14) | 209.24 (291.23 to 149.76) | 18.92 (26.35 to 13.53) | -0.01 (-0.12 to 0.1) | 3.86 (5.66 to 2.56) | 0.35 (0.52 to 0.23) | -0.05 (-0.2 to 0.09) |
| Spain | 766.04 (1211.07 to 430.03) | 6.61 (10.48 to 3.70) | 0.87 (0.69 to 1.05) | 5801.09 (8575.38 to 3719.09) | 50.04 (74.16 to 32.05) | 0.82 (0.66 to 0.98) | 1730.70 (2472.05 to 1243.83) | 14.85 (21.27 to 10.65) | 0.17 (0.05 to 0.29) | 20.31 (21.86 to 18.74) | 0.17 (0.19 to 0.16) | -0.54 (-0.83 to -0.26) |
| Sri Lanka | 213.49 (328.92 to 121.80) | 4.93 (7.62 to 2.80) | 0.6 (0.55 to 0.66) | 1609.74 (2376.67 to 1068.11) | 37.15 (54.86 to 24.65) | 0.5 (0.46 to 0.53) | 752.25 (1035.83 to 513.16) | 17.37 (23.92 to 11.85) | -0.38 (-0.73 to -0.03) | 11.90 (17.21 to 7.26) | 0.27 (0.40 to 0.17) | -0.83 (-1.16 to -0.5) |
| Sudan | 292.59 (415.82 to 185.04) | 5.84 (8.28 to 3.70) | 0.73 (0.67 to 0.79) | 2097.03 (2954.12 to 1421.55) | 42.10 (59.10 to 28.68) | 0.76 (0.69 to 0.84) | 1250.50 (1730.06 to 873.05) | 25.42 (35.15 to 17.78) | -0.24 (-0.3 to -0.19) | 22.35 (32.81 to 14.60) | 0.46 (0.67 to 0.30) | -0.56 (-0.62 to -0.5) |
| Suriname | 6.52 (9.70 to 3.85) | 5.76 (8.61 to 3.40) | 0.67 (0.54 to 0.8) | 57.12 (81.21 to 38.44) | 49.92 (71.30 to 33.50) | 0.54 (0.48 to 0.6) | 24.98 (33.75 to 18.27) | 21.64 (29.23 to 15.82) | 0.63 (-0.21 to 1.47) | 0.39 (0.51 to 0.29) | 0.34 (0.43 to 0.25) | 0.67 (-0.59 to 1.93) |
| Sweden | 107.90 (157.76 to 64.62) | 5.28 (7.76 to 3.15) | 0.51 (0.47 to 0.54) | 888.13 (1233.58 to 607.04) | 43.43 (60.44 to 29.67) | 0.36 (0.34 to 0.38) | 260.30 (359.32 to 189.04) | 12.60 (17.42 to 9.12) | -0.53 (-0.66 to -0.39) | 2.97 (3.34 to 2.63) | 0.14 (0.16 to 0.12) | -1.3 (-1.58 to -1.01) |
| Switzerland | 118.51 (181.16 to 68.60) | 5.96 (9.18 to 3.42) | 0.49 (0.41 to 0.56) | 924.86 (1346.00 to 611.53) | 46.41 (67.80 to 30.65) | 0.41 (0.38 to 0.45) | 249.25 (362.26 to 169.48) | 12.40 (18.13 to 8.39) | -0.46 (-0.63 to -0.28) | 2.60 (2.78 to 2.41) | 0.13 (0.14 to 0.12) | -1.34 (-1.67 to -1.01) |
| Syrian Arab Republic | 151.66 (228.04 to 91.78) | 5.11 (7.69 to 3.09) | 1.04 (0.99 to 1.09) | 1115.09 (1644.27 to 744.88) | 37.49 (55.37 to 25.01) | 1.04 (1.01 to 1.06) | 676.13 (909.84 to 496.31) | 22.64 (30.48 to 16.62) | -0.36 (-0.52 to -0.19) | 12.31 (17.32 to 8.62) | 0.41 (0.58 to 0.29) | -0.7 (-0.9 to -0.5) |
| Taiwan (Province of China) | 435.16 (555.67 to 324.83) | 7.54 (9.67 to 5.60) | 1.77 (1.65 to 1.89) | 2683.92 (3436.27 to 2068.86) | 46.50 (59.55 to 35.82) | 1.37 (1.26 to 1.48) | 1547.26 (1865.90 to 1316.82) | 26.89 (32.42 to 22.89) | 0.03 (-0.02 to 0.09) | 27.23 (29.65 to 24.62) | 0.47 (0.51 to 0.43) | -0.37 (-0.44 to -0.3) |
| Tajikistan | 50.59 (78.78 to 28.14) | 3.69 (5.74 to 2.05) | 0 (-0.08 to 0.08) | 360.97 (532.73 to 230.55) | 26.31 (38.80 to 16.81) | -0.02 (-0.07 to 0.03) | 215.81 (275.77 to 168.03) | 15.73 (20.10 to 12.25) | -0.5 (-0.62 to -0.37) | 3.84 (5.10 to 2.99) | 0.28 (0.37 to 0.22) | -0.67 (-0.84 to -0.49) |
| Thailand | 863.11 (1270.73 to 511.91) | 5.01 (7.42 to 2.95) | 0.37 (0.32 to 0.41) | 6452.04 (9009.84 to 4345.28) | 37.55 (52.59 to 25.25) | 0.39 (0.38 to 0.41) | 4209.44 (5493.20 to 3197.73) | 24.80 (32.31 to 18.85) | 0.1 (-0.09 to 0.3) | 76.80 (104.37 to 55.95) | 0.45 (0.61 to 0.33) | -0.03 (-0.26 to 0.21) |
| Timor-Leste | 7.24 (11.13 to 4.11) | 4.49 (6.86 to 2.57) | 0.48 (0.43 to 0.53) | 52.28 (76.69 to 33.81) | 32.38 (47.53 to 20.95) | 0.37 (0.35 to 0.39) | 30.09 (39.83 to 21.92) | 18.62 (24.66 to 13.58) | -0.06 (-0.24 to 0.12) | 0.52 (0.72 to 0.37) | 0.32 (0.45 to 0.23) | -0.22 (-0.52 to 0.08) |
| Togo | 31.69 (50.10 to 16.23) | 3.06 (4.82 to 1.57) | 0.59 (0.43 to 0.74) | 289.10 (411.69 to 188.36) | 27.87 (39.61 to 18.20) | 0.57 (0.49 to 0.65) | 209.87 (316.51 to 138.46) | 19.59 (29.43 to 12.98) | 0.93 (0.86 to 1.01) | 3.71 (5.91 to 2.24) | 0.35 (0.55 to 0.21) | 1.02 (0.92 to 1.13) |
| Tokelau | 0.01 (0.02 to 0.01) | 5.67 (8.19 to 3.34) | 0.18 (0.05 to 0.31) | 0.09 (0.12 to 0.06) | 36.39 (51.77 to 24.10) | 0.28 (0.19 to 0.36) | 0.05 (0.07 to 0.04) | 21.20 (29.64 to 15.15) | -0.73 (-0.77 to -0.69) | 0.00 (0.00 to 0.00) | 0.39 (0.57 to 0.25) | -1.02 (-1.1 to -0.95) |
| Tonga | 0.80 (1.19 to 0.47) | 5.25 (7.84 to 3.08) | 0.4 (0.28 to 0.53) | 5.32 (7.72 to 3.44) | 34.93 (50.74 to 22.60) | 0.46 (0.4 to 0.53) | 2.91 (4.21 to 2.02) | 18.97 (27.46 to 13.19) | -0.11 (-0.16 to -0.06) | 0.05 (0.08 to 0.03) | 0.33 (0.52 to 0.21) | -0.32 (-0.37 to -0.27) |
| Trinidad and Tobago | 17.03 (24.91 to 10.33) | 6.20 (9.09 to 3.76) | 0.5 (0.34 to 0.66) | 147.12 (204.61 to 100.40) | 53.56 (74.47 to 36.56) | 0.33 (0.18 to 0.48) | 61.55 (81.80 to 46.31) | 22.40 (29.77 to 16.85) | 0.35 (-0.29 to 1) | 0.94 (1.24 to 0.70) | 0.34 (0.45 to 0.25) | 0.58 (-0.18 to 1.35) |
| Tunisia | 130.84 (192.34 to 80.40) | 5.53 (8.14 to 3.40) | 1.13 (1.08 to 1.19) | 964.80 (1394.79 to 645.77) | 40.85 (58.97 to 27.37) | 1.1 (1.06 to 1.13) | 473.35 (654.72 to 336.96) | 20.08 (27.77 to 14.29) | 0.26 (0.19 to 0.33) | 7.80 (11.30 to 5.01) | 0.33 (0.48 to 0.21) | -0.06 (-0.15 to 0.03) |
| Turkey | 884.04 (1330.58 to 522.31) | 5.38 (8.08 to 3.19) | 0.79 (0.73 to 0.84) | 6661.86 (9587.66 to 4460.55) | 40.68 (58.48 to 27.26) | 0.79 (0.74 to 0.84) | 3335.50 (4320.84 to 2524.83) | 20.46 (26.48 to 15.49) | -0.73 (-0.83 to -0.63) | 55.73 (72.13 to 42.54) | 0.34 (0.45 to 0.26) | -1.24 (-1.35 to -1.12) |
| Turkmenistan | 31.39 (48.89 to 17.93) | 3.71 (5.77 to 2.12) | -0.01 (-0.06 to 0.04) | 229.87 (339.23 to 149.41) | 27.16 (40.09 to 17.65) | 0.14 (0.11 to 0.17) | 140.05 (181.30 to 105.64) | 16.55 (21.42 to 12.48) | 0.56 (-0.16 to 1.28) | 2.50 (3.33 to 1.83) | 0.30 (0.39 to 0.22) | 0.69 (-0.45 to 1.83) |
| Tuvalu | 0.11 (0.17 to 0.07) | 6.10 (8.93 to 3.84) | 0.23 (0.18 to 0.28) | 0.73 (1.06 to 0.49) | 38.94 (56.67 to 26.26) | 0.37 (0.33 to 0.41) | 0.50 (0.66 to 0.37) | 26.49 (35.25 to 19.68) | -0.53 (-0.58 to -0.48) | 0.01 (0.01 to 0.01) | 0.51 (0.69 to 0.37) | -0.76 (-0.81 to -0.71) |
| Uganda | 140.42 (221.67 to 77.48) | 3.74 (5.87 to 2.07) | 0.38 (0.32 to 0.43) | 1204.29 (1772.52 to 795.26) | 32.15 (47.19 to 21.29) | 0.33 (0.3 to 0.36) | 668.62 (919.49 to 459.13) | 18.06 (24.82 to 12.40) | 0.12 (0.01 to 0.22) | 11.47 (16.71 to 7.21) | 0.31 (0.46 to 0.20) | 0.03 (-0.09 to 0.16) |
| Ukraine | 604.48 (852.43 to 391.87) | 6.31 (8.91 to 4.08) | 0.11 (-0.08 to 0.3) | 3867.81 (5330.75 to 2651.10) | 40.32 (55.63 to 27.61) | 0.1 (0.06 to 0.14) | 1776.91 (2278.10 to 1302.61) | 18.50 (23.73 to 13.57) | 0.38 (-0.67 to 1.45) | 28.90 (38.57 to 20.13) | 0.30 (0.40 to 0.21) | 0.07 (-0.98 to 1.14) |
| United Arab Emirates | 276.48 (403.58 to 177.65) | 8.72 (12.61 to 5.64) | 1.04 (0.92 to 1.15) | 1856.27 (2637.83 to 1280.27) | 59.66 (84.08 to 41.57) | 1.18 (1.14 to 1.21) | 465.90 (685.89 to 303.58) | 15.06 (22.16 to 9.72) | -1.75 (-1.95 to -1.56) | 4.18 (5.41 to 3.14) | 0.14 (0.18 to 0.11) | -3.85 (-4.24 to -3.46) |
| United Kingdom | 954.43 (1346.92 to 608.75) | 6.76 (9.61 to 4.26) | -0.23 (-0.29 to -0.16) | 7485.53 (10248.41 to 5288.40) | 52.88 (72.61 to 37.33) | 0.09 (0.02 to 0.15) | 2580.85 (3160.39 to 2128.35) | 18.03 (22.16 to 14.80) | 0.12 (-0.37 to 0.61) | 34.94 (35.69 to 34.20) | 0.24 (0.24 to 0.23) | 0.19 (-0.26 to 0.64) |
| United Republic of Tanzania | 226.28 (341.81 to 125.40) | 3.55 (5.34 to 1.97) | 0.56 (0.52 to 0.6) | 1989.15 (2830.74 to 1334.69) | 31.33 (44.38 to 21.08) | 0.36 (0.34 to 0.39) | 1057.48 (1446.06 to 751.70) | 16.84 (23.01 to 11.98) | -0.15 (-0.44 to 0.14) | 17.85 (26.36 to 11.80) | 0.29 (0.42 to 0.19) | -0.37 (-0.73 to 0) |
| United States Virgin Islands | 1.65 (2.34 to 1.14) | 4.78 (5.88 to 3.79) | 0.27 (0.24 to 0.29) | 18081.02 (21643.24 to 14920.19) | 27.67 (33.16 to 22.78) | 0.53 (0.5 to 0.57) | 4.36 (6.04 to 3.13) | 19.01 (20.75 to 17.46) | -0.87 (-1.32 to -0.42) | 235.18 (241.64 to 228.56) | 0.36 (0.37 to 0.35) | -1.86 (-2.47 to -1.24) |
| United States of America | 3117.82 (3815.83 to 2486.68) | 8.99 (12.88 to 6.17) | 0.73 (0.69 to 0.77) | 13.62 (18.58 to 9.86) | 72.23 (98.81 to 51.92) | -0.73 (-0.8 to -0.67) | 12412.22 (13544.62 to 11404.71) | 23.48 (32.55 to 16.82) | 0.02 (-0.22 to 0.27) | 0.06 (0.07 to 0.04) | 0.30 (0.40 to 0.22) | 0.3 (0.04 to 0.55) |
| Uruguay | 34.16 (51.87 to 20.03) | 5.17 (7.87 to 3.02) | 0.95 (0.74 to 1.16) | 205.15 (305.42 to 129.33) | 31.13 (46.41 to 19.59) | 0.98 (0.87 to 1.09) | 99.77 (124.14 to 81.56) | 15.12 (18.82 to 12.35) | 0.34 (0.08 to 0.6) | 1.65 (1.77 to 1.53) | 0.25 (0.27 to 0.23) | 0.04 (-0.39 to 0.47) |
| Uzbekistan | 216.82 (329.54 to 119.61) | 3.81 (5.78 to 2.10) | -0.08 (-0.21 to 0.04) | 1529.51 (2243.44 to 968.44) | 26.86 (39.36 to 17.01) | -0.13 (-0.2 to -0.07) | 615.32 (788.22 to 475.07) | 10.82 (13.85 to 8.35) | -0.64 (-0.93 to -0.36) | 8.96 (10.74 to 7.29) | 0.16 (0.19 to 0.13) | -1.03 (-1.4 to -0.65) |
| Vanuatu | 2.61 (3.74 to 1.72) | 6.24 (8.90 to 4.13) | 0.41 (0.27 to 0.55) | 16.65 (23.19 to 11.36) | 39.86 (55.46 to 27.25) | 0.4 (0.36 to 0.43) | 11.69 (14.99 to 8.80) | 28.35 (36.30 to 21.35) | -0.17 (-0.45 to 0.12) | 0.22 (0.30 to 0.16) | 0.55 (0.72 to 0.39) | -0.31 (-0.65 to 0.02) |
| Venezuela (Bolivarian Republic of) | 396.11 (580.99 to 234.27) | 7.32 (10.75 to 4.32) | 0.84 (0.6 to 1.09) | 3427.05 (4860.83 to 2333.69) | 63.26 (89.71 to 43.08) | 0.79 (0.64 to 0.94) | 1237.43 (1679.72 to 887.70) | 22.84 (31.00 to 16.38) | 0.55 (0.25 to 0.86) | 17.07 (22.78 to 12.64) | 0.31 (0.42 to 0.23) | 0.55 (0.07 to 1.03) |
| Viet Nam | 1218.11 (1844.84 to 713.10) | 6.15 (9.33 to 3.60) | 1.02 (0.98 to 1.05) | 8727.93 (12625.32 to 5986.58) | 44.09 (63.80 to 30.23) | 0.86 (0.83 to 0.9) | 4858.71 (6578.95 to 3523.47) | 24.55 (33.26 to 17.81) | 0.33 (0.29 to 0.38) | 83.99 (117.89 to 59.33) | 0.42 (0.60 to 0.30) | 0.16 (0.13 to 0.2) |
| Yemen | 181.71 (256.38 to 117.59) | 5.10 (7.17 to 3.32) | 0.88 (0.82 to 0.95) | 1316.24 (1830.64 to 899.90) | 37.44 (51.76 to 25.80) | 0.93 (0.91 to 0.96) | 855.99 (1183.44 to 581.48) | 25.08 (34.62 to 17.03) | -0.1 (-0.28 to 0.08) | 15.72 (23.64 to 9.61) | 0.47 (0.71 to 0.29) | -0.37 (-0.55 to -0.18) |
| Zambia | 78.25 (118.21 to 43.76) | 3.99 (5.99 to 2.24) | 0.3 (0.22 to 0.38) | 657.76 (932.02 to 428.39) | 33.71 (47.55 to 22.06) | 0.3 (0.25 to 0.36) | 369.24 (495.76 to 270.49) | 19.18 (25.74 to 14.05) | 0.11 (-0.02 to 0.25) | 6.33 (8.90 to 4.33) | 0.33 (0.47 to 0.23) | 0.01 (-0.14 to 0.16) |
| Zimbabwe | 63.24 (98.06 to 33.75) | 3.59 (5.54 to 1.92) | 0.19 (0.08 to 0.3) | 552.45 (790.45 to 367.36) | 31.50 (44.91 to 21.01) | 0.2 (0.14 to 0.27) | 474.92 (653.88 to 330.89) | 27.46 (37.74 to 19.20) | 1.43 (1.16 to 1.7) | 9.23 (13.26 to 6.06) | 0.54 (0.78 to 0.36) | 1.63 (1.27 to 2) |

# Table S3. Changes in DALYs number according to population-level determinants and causes from 1990 to 2021.

| Location | Overll difference | Change due to Population-level determinants  (% contribute to the total changes) | | |
| --- | --- | --- | --- | --- |
|  |  | Aging | Population | Epidemiological change |
| Global | 186559.80 | 9365.66(5.02%) | 156090.57(83.67%) | 21103.57(11.31%) |
| **SDI** |  |  |  |  |
| High SDI | 17013.92 | 2508.58(14.74%) | 11480.08(67.47%) | 3025.27(17.78%) |
| High-middle SDI | 41342.86 | 2179.18(5.27%) | 31794.41(76.90%) | 7369.26(17.82%) |
| Middle SDI | 79558.32 | 5909.55(7.43%) | 70424.27(88.52%) | 3224.50(4.05%) |
| Low-middle SDI | 36444.92 | 288.09(0.79%) | 32508.54(89.20%) | 3648.29(10.01%) |
| Low SDI | 12108.72 | 365.12(3.02%) | 13477.35(111.30%) | 1003.51(8.29%) |

# Table S4. Frontier age-standardized disability-adjusted life-years and corresponding effective difference of EOPD in 2021 in 204 countries or territories.

| Location | Sociodemographic-index | Age-standardized disability adjusted life-years | Frontier age standardized disability adjusted life-years | Effective difference | Trend of age standardized  disability adjusted life years from 1990  to 2019 | Effective difference rank (Age-standardized disability adjusted life-years rank) |
| --- | --- | --- | --- | --- | --- | --- |
| Afghanistan | 0.34 | 48.35 | 14.36 | 33.99 | Decrease | 203(204) |
| Albania | 0.71 | 10.86 | 10.52 | 0.34 | Decrease | 8(7) |
| Algeria | 0.66 | 22.32 | 11.07 | 11.25 | Increase | 151(141) |
| American Samoa | 0.72 | 20.41 | 10.83 | 9.57 | Decrease | 129(123) |
| Andorra | 0.87 | 13.65 | 10.13 | 3.52 | Decrease | 42(29) |
| Angola | 0.45 | 19.81 | 13.48 | 6.33 | Decrease | 97(111) |
| Antigua and Barbuda | 0.75 | 19.81 | 10.76 | 9.05 | Decrease | 119(110) |
| Argentina | 0.72 | 12.60 | 10.57 | 2.03 | Decrease | 24(20) |
| Armenia | 0.70 | 10.44 | 10.44 | 0.00 | Decrease | 1(3) |
| Australia | 0.84 | 12.22 | 10.30 | 1.92 | Increase | 22(15) |
| Austria | 0.85 | 13.48 | 10.17 | 3.31 | Decrease | 38(28) |
| Azerbaijan | 0.69 | 10.52 | 10.52 | 0.00 | Decrease | 1(4) |
| Bahamas | 0.81 | 28.27 | 10.67 | 17.60 | Increase | 188(186) |
| Bahrain | 0.75 | 16.48 | 10.72 | 5.76 | Decrease | 87(64) |
| Bangladesh | 0.49 | 17.70 | 13.45 | 4.25 | Decrease | 54(79) |
| Barbados | 0.75 | 18.69 | 10.76 | 7.93 | Increase | 111(92) |
| Belarus | 0.78 | 14.57 | 10.72 | 3.85 | Increase | 50(43) |
| Belgium | 0.85 | 15.02 | 10.16 | 4.86 | Increase | 70(50) |
| Belize | 0.61 | 24.74 | 11.71 | 13.03 | Increase | 168(166) |
| Benin | 0.37 | 18.75 | 13.88 | 4.88 | Increase | 71(94) |
| Bermuda | 0.82 | 17.22 | 10.66 | 6.55 | Decrease | 98(73) |
| Bhutan | 0.47 | 20.12 | 13.50 | 6.62 | Decrease | 100(117) |
| Bolivia | 0.60 | 34.12 | 11.78 | 22.35 | Decrease | 198(197) |
| Bosnia and Herzegovina | 0.72 | 13.03 | 10.75 | 2.28 | Decrease | 27(24) |
| Botswana | 0.64 | 16.51 | 11.18 | 5.33 | Decrease | 82(65) |
| Brazil | 0.65 | 21.56 | 11.15 | 10.41 | Increase | 138(131) |
| Brunei Darussalam | 0.81 | 24.96 | 10.71 | 14.25 | Increase | 177(169) |
| Bulgaria | 0.77 | 17.44 | 10.68 | 6.76 | Increase | 103(76) |
| Burkina Faso | 0.29 | 18.41 | 14.58 | 3.83 | Increase | 48(88) |
| Burundi | 0.29 | 17.90 | 14.52 | 3.38 | Decrease | 40(82) |
| Cabo Verde | 0.53 | 21.97 | 13.02 | 8.95 | Increase | 118(140) |
| Cambodia | 0.47 | 24.07 | 13.49 | 10.58 | Decrease | 142(160) |
| Cameroon | 0.48 | 24.04 | 13.45 | 10.59 | Increase | 144(159) |
| Canada | 0.87 | 22.45 | 10.15 | 12.30 | Increase | 162(145) |
| Central African Republic | 0.31 | 25.06 | 14.56 | 10.50 | Decrease | 140(170) |
| Chad | 0.24 | 19.84 | 14.58 | 5.26 | Increase | 79(113) |
| Chile | 0.77 | 12.68 | 10.73 | 1.95 | Decrease | 23(21) |
| China | 0.72 | 35.03 | 10.80 | 24.23 | Increase | 200(199) |
| Colombia | 0.66 | 19.11 | 11.12 | 7.99 | Decrease | 114(102) |
| Comoros | 0.48 | 17.84 | 13.41 | 4.43 | Decrease | 61(81) |
| Congo | 0.58 | 22.41 | 11.82 | 10.59 | Decrease | 143(144) |
| Cook Islands | 0.78 | 17.40 | 10.73 | 6.67 | Decrease | 101(75) |
| Costa Rica | 0.70 | 21.77 | 10.77 | 11.00 | Increase | 147(136) |
| Coted'Ivoire | 0.43 | 22.83 | 13.58 | 9.25 | Increase | 123(150) |
| Croatia | 0.80 | 11.10 | 10.51 | 0.59 | Decrease | 11(8) |
| Cuba | 0.67 | 19.09 | 11.12 | 7.97 | Increase | 113(101) |
| Cyprus | 0.84 | 12.01 | 10.46 | 1.55 | Decrease | 18(14) |
| Czechia | 0.83 | 10.58 | 10.50 | 0.09 | Decrease | 7(5) |
| Democratic People's Republic of Korea | 0.57 | 43.17 | 11.88 | 31.29 | Increase | 202(202) |
| Democratic Republic of the Congo | 0.38 | 19.22 | 13.90 | 5.32 | Decrease | 81(104) |
| Denmark | 0.90 | 12.97 | 9.91 | 3.06 | Decrease | 34(23) |
| Djibouti | 0.49 | 16.66 | 13.38 | 3.28 | Increase | 35(68) |
| Dominica | 0.75 | 25.73 | 10.55 | 15.18 | Increase | 181(176) |
| Dominican Republic | 0.62 | 23.30 | 11.33 | 11.98 | Increase | 160(155) |
| Ecuador | 0.66 | 30.57 | 11.06 | 19.51 | Increase | 193(191) |
| Egypt | 0.61 | 23.21 | 11.71 | 11.50 | Decrease | 154(154) |
| El Salvador | 0.56 | 27.54 | 12.15 | 15.39 | Increase | 182(181) |
| Equatorial Guinea | 0.66 | 20.39 | 11.06 | 9.33 | Decrease | 125(121) |
| Eritrea | 0.40 | 23.19 | 13.79 | 9.39 | Decrease | 126(153) |
| Estonia | 0.84 | 11.87 | 10.19 | 1.68 | Decrease | 19(13) |
| Eswatini | 0.59 | 33.97 | 11.78 | 22.20 | Increase | 196(196) |
| Ethiopia | 0.36 | 14.65 | 13.87 | 0.78 | Decrease | 13(45) |
| Fiji | 0.68 | 21.63 | 11.06 | 10.57 | Decrease | 141(134) |
| Finland | 0.86 | 15.43 | 10.11 | 5.32 | Decrease | 80(56) |
| France | 0.84 | 15.39 | 10.49 | 4.90 | Increase | 72(54) |
| Gabon | 0.63 | 24.09 | 11.28 | 12.81 | Decrease | 167(161) |
| Gambia | 0.41 | 19.89 | 13.69 | 6.20 | Increase | 94(114) |
| Georgia | 0.73 | 14.47 | 10.73 | 3.74 | Decrease | 47(41) |
| Germany | 0.90 | 16.30 | 10.02 | 6.28 | Increase | 96(62) |
| Ghana | 0.56 | 16.97 | 11.97 | 5.00 | Increase | 75(71) |
| Greece | 0.79 | 15.46 | 10.64 | 4.82 | Increase | 69(57) |
| Greenland | 0.83 | 27.64 | 10.62 | 17.02 | Decrease | 186(182) |
| Grenada | 0.67 | 22.65 | 11.02 | 11.63 | Increase | 157(148) |
| Guam | 0.80 | 18.45 | 10.56 | 7.89 | Increase | 110(89) |
| Guatemala | 0.54 | 21.58 | 13.01 | 8.58 | Decrease | 116(132) |
| Guinea | 0.34 | 18.90 | 14.17 | 4.73 | Increase | 67(96) |
| Guinea-Bissau | 0.35 | 29.50 | 13.91 | 15.59 | Increase | 184(189) |
| Guyana | 0.65 | 29.34 | 11.20 | 18.15 | Increase | 189(188) |
| Haiti | 0.45 | 28.08 | 13.53 | 14.55 | Decrease | 179(185) |
| Honduras | 0.51 | 35.49 | 13.15 | 22.33 | Increase | 197(200) |
| Hungary | 0.79 | 12.90 | 10.69 | 2.22 | Decrease | 25(22) |
| Iceland | 0.88 | 15.55 | 10.19 | 5.36 | Decrease | 83(58) |
| India | 0.58 | 21.79 | 11.88 | 9.91 | Increase | 132(137) |
| Indonesia | 0.66 | 22.48 | 11.16 | 11.32 | Increase | 153(146) |
| Iran (Islamic Republic of) | 0.70 | 20.31 | 10.82 | 9.49 | Decrease | 128(118) |
| Iraq | 0.66 | 29.93 | 11.11 | 18.82 | Decrease | 192(190) |
| Ireland | 0.87 | 13.74 | 10.12 | 3.62 | Decrease | 44(31) |
| Israel | 0.81 | 14.95 | 10.67 | 4.28 | Decrease | 56(48) |
| Italy | 0.81 | 14.44 | 10.76 | 3.68 | Decrease | 46(39) |
| Jamaica | 0.68 | 20.32 | 11.05 | 9.27 | Increase | 124(119) |
| Japan | 0.87 | 14.37 | 9.98 | 4.39 | Increase | 58(38) |
| Jordan | 0.73 | 16.67 | 10.74 | 5.93 | Decrease | 90(69) |
| Kazakhstan | 0.73 | 16.21 | 10.81 | 5.40 | Decrease | 84(61) |
| Kenya | 0.52 | 16.97 | 13.06 | 3.91 | Increase | 51(72) |
| Kiribati | 0.53 | 24.71 | 13.07 | 11.64 | Decrease | 158(165) |
| Kuwait | 0.85 | 14.47 | 10.39 | 4.09 | Decrease | 52(40) |
| Kyrgyzstan | 0.60 | 12.23 | 11.78 | 0.45 | Decrease | 9(16) |
| Lao People's Democratic Republic | 0.49 | 23.92 | 13.43 | 10.49 | Decrease | 139(158) |
| Latvia | 0.83 | 13.92 | 10.46 | 3.46 | Decrease | 41(34) |
| Lebanon | 0.74 | 16.65 | 10.80 | 5.84 | Decrease | 88(67) |
| Lesotho | 0.51 | 25.66 | 13.04 | 12.62 | Increase | 164(175) |
| Liberia | 0.35 | 19.43 | 13.93 | 5.50 | Increase | 85(105) |
| Libya | 0.73 | 34.82 | 10.77 | 24.05 | Increase | 199(198) |
| Lithuania | 0.86 | 13.42 | 10.10 | 3.31 | Decrease | 39(26) |
| Luxembourg | 0.88 | 13.74 | 10.44 | 3.30 | Decrease | 36(30) |
| Madagascar | 0.40 | 14.94 | 13.80 | 1.14 | Decrease | 16(47) |
| Malawi | 0.38 | 18.78 | 13.83 | 4.95 | Increase | 73(95) |
| Malaysia | 0.74 | 20.90 | 10.52 | 10.38 | Increase | 137(126) |
| Maldives | 0.65 | 13.74 | 11.19 | 2.55 | Decrease | 29(32) |
| Mali | 0.27 | 18.69 | 14.58 | 4.11 | Decrease | 53(93) |
| Malta | 0.80 | 15.62 | 10.65 | 4.97 | Increase | 74(59) |
| Marshall Islands | 0.57 | 30.79 | 12.14 | 18.66 | Decrease | 190(193) |
| Mauritania | 0.50 | 13.99 | 13.40 | 0.59 | Decrease | 10(36) |
| Mauritius | 0.72 | 22.39 | 10.84 | 11.55 | Increase | 156(142) |
| Mexico | 0.66 | 24.92 | 10.90 | 14.02 | Increase | 174(168) |
| Micronesia (Federated States of) | 0.59 | 27.37 | 11.79 | 15.58 | Decrease | 183(179) |
| Monaco | 0.91 | 18.18 | 9.94 | 8.24 | Increase | 115(87) |
| Mongolia | 0.62 | 13.98 | 11.41 | 2.57 | Decrease | 30(35) |
| Montenegro | 0.80 | 13.17 | 10.73 | 2.44 | Decrease | 28(25) |
| Morocco | 0.56 | 30.68 | 12.00 | 18.68 | Increase | 191(192) |
| Mozambique | 0.33 | 21.34 | 14.56 | 6.78 | Increase | 104(130) |
| Myanmar | 0.53 | 23.82 | 13.13 | 10.69 | Decrease | 146(157) |
| Namibia | 0.62 | 24.09 | 11.36 | 12.73 | Increase | 166(162) |
| Nauru | 0.63 | 36.19 | 11.31 | 24.88 | Decrease | 201(201) |
| Nepal | 0.43 | 19.45 | 13.50 | 5.95 | Increase | 91(106) |
| Netherlands | 0.89 | 14.62 | 9.92 | 4.70 | Decrease | 66(44) |
| New Zealand | 0.85 | 13.84 | 10.00 | 3.84 | Increase | 49(33) |
| Nicaragua | 0.52 | 20.04 | 13.00 | 7.04 | Increase | 105(115) |
| Niger | 0.17 | 16.36 | 14.65 | 1.70 | Decrease | 20(63) |
| Nigeria | 0.50 | 17.78 | 13.09 | 4.69 | Decrease | 65(80) |
| Niue | 0.73 | 22.96 | 10.75 | 12.21 | Decrease | 161(152) |
| North Macedonia | 0.75 | 11.62 | 10.69 | 0.93 | Decrease | 14(11) |
| Northern Mariana Islands | 0.77 | 19.82 | 10.71 | 9.12 | Decrease | 121(112) |
| Norway | 0.92 | 14.49 | 9.99 | 4.50 | Increase | 62(42) |
| Oman | 0.77 | 21.29 | 10.67 | 10.62 | Decrease | 145(129) |
| Pakistan | 0.50 | 22.70 | 13.07 | 9.64 | Increase | 130(149) |
| Palau | 0.75 | 20.81 | 10.67 | 10.14 | Decrease | 134(125) |
| Palestine | 0.63 | 17.67 | 11.44 | 6.23 | Decrease | 95(78) |
| Panama | 0.71 | 21.60 | 10.50 | 11.09 | Increase | 149(133) |
| Papua New Guinea | 0.42 | 21.11 | 13.61 | 7.50 | Decrease | 107(127) |
| Paraguay | 0.64 | 20.39 | 11.22 | 9.17 | Increase | 122(122) |
| Peru | 0.66 | 33.16 | 11.04 | 22.12 | Increase | 195(195) |
| Philippines | 0.65 | 25.40 | 11.17 | 14.23 | Increase | 176(173) |
| Poland | 0.81 | 13.48 | 10.72 | 2.76 | Decrease | 32(27) |
| Portugal | 0.74 | 14.11 | 10.56 | 3.55 | Increase | 43(37) |
| Puerto Rico | 0.83 | 21.82 | 10.72 | 11.10 | Increase | 150(139) |
| Qatar | 0.85 | 18.04 | 10.16 | 7.88 | Decrease | 109(84) |
| Republic of Korea | 0.89 | 15.02 | 9.94 | 5.07 | Decrease | 76(49) |
| Republic of Moldova | 0.73 | 12.50 | 10.76 | 1.74 | Decrease | 21(18) |
| Romania | 0.77 | 15.42 | 10.68 | 4.74 | Increase | 68(55) |
| Russian Federation | 0.81 | 15.12 | 10.73 | 4.39 | Increase | 59(53) |
| Rwanda | 0.44 | 18.18 | 13.57 | 4.61 | Decrease | 64(86) |
| Saint Kitts and Nevis | 0.75 | 27.83 | 10.69 | 17.14 | Decrease | 187(183) |
| Saint Lucia | 0.67 | 24.65 | 10.97 | 13.67 | Decrease | 170(164) |
| Saint Vincent and the Grenadines | 0.64 | 25.18 | 11.17 | 14.02 | Increase | 173(172) |
| Samoa | 0.59 | 21.81 | 11.81 | 10.01 | Decrease | 133(138) |
| San Marino | 0.89 | 9.56 | 9.56 | 0.00 | Decrease | 1(1) |
| Sao Tome and Principe | 0.51 | 19.80 | 13.09 | 6.71 | Increase | 102(109) |
| Saudi Arabia | 0.82 | 44.97 | 10.67 | 34.30 | Increase | 204(203) |
| Senegal | 0.41 | 19.78 | 13.81 | 5.98 | Increase | 92(108) |
| Serbia | 0.79 | 11.66 | 10.69 | 0.97 | Decrease | 15(12) |
| Seychelles | 0.73 | 31.49 | 10.84 | 20.66 | Decrease | 194(194) |
| Sierra Leone | 0.36 | 17.47 | 13.84 | 3.63 | Increase | 45(77) |
| Singapore | 0.86 | 11.36 | 10.10 | 1.26 | Decrease | 17(9) |
| Slovakia | 0.81 | 11.46 | 10.70 | 0.75 | Decrease | 12(10) |
| Slovenia | 0.84 | 9.95 | 9.95 | 0.00 | Decrease | 5(2) |
| Solomon Islands | 0.43 | 27.88 | 13.61 | 14.27 | Decrease | 178(184) |
| Somalia | 0.08 | 18.97 | 18.89 | 0.08 | Decrease | 6(99) |
| South Africa | 0.68 | 20.33 | 10.89 | 9.44 | Increase | 127(120) |
| South Sudan | 0.28 | 18.92 | 14.50 | 4.42 | Decrease | 60(97) |
| Spain | 0.77 | 14.85 | 10.59 | 4.26 | Increase | 55(46) |
| Sri Lanka | 0.70 | 17.37 | 10.79 | 6.59 | Decrease | 99(74) |
| Sudan | 0.54 | 25.42 | 13.01 | 12.41 | Decrease | 163(174) |
| Suriname | 0.63 | 21.64 | 11.45 | 10.19 | Increase | 135(135) |
| Sweden | 0.89 | 12.60 | 9.93 | 2.67 | Decrease | 31(19) |
| Switzerland | 0.93 | 12.40 | 10.14 | 2.25 | Decrease | 26(17) |
| Syrian Arab Republic | 0.62 | 22.64 | 11.39 | 11.26 | Decrease | 152(147) |
| Taiwan (Province of China) | 0.87 | 26.89 | 10.14 | 16.75 | Decrease | 185(178) |
| Tajikistan | 0.54 | 15.73 | 12.73 | 3.00 | Decrease | 33(60) |
| Thailand | 0.68 | 24.80 | 10.99 | 13.81 | Increase | 171(167) |
| Timor-Leste | 0.44 | 18.62 | 13.52 | 5.10 | Decrease | 77(91) |
| Togo | 0.41 | 19.59 | 13.66 | 5.93 | Increase | 89(107) |
| Tokelau | 0.69 | 21.20 | 10.87 | 10.32 | Decrease | 136(128) |
| Tonga | 0.63 | 18.97 | 11.65 | 7.32 | Decrease | 106(98) |
| Trinidad and Tobago | 0.77 | 22.40 | 10.61 | 11.79 | Increase | 159(143) |
| Tunisia | 0.68 | 20.08 | 11.01 | 9.07 | Increase | 120(116) |
| Turkey | 0.71 | 20.46 | 10.73 | 9.73 | Decrease | 131(124) |
| Turkmenistan | 0.68 | 16.55 | 10.97 | 5.57 | Increase | 86(66) |
| Tuvalu | 0.58 | 26.49 | 12.27 | 14.22 | Decrease | 175(177) |
| Uganda | 0.42 | 18.06 | 13.56 | 4.50 | Increase | 63(85) |
| Ukraine | 0.76 | 18.50 | 10.67 | 7.83 | Increase | 108(90) |
| United Arab Emirates | 0.85 | 15.06 | 9.93 | 5.13 | Decrease | 78(51) |
| United Kingdom | 0.86 | 18.03 | 10.07 | 7.95 | Increase | 112(83) |
| United Republic of Tanzania | 0.45 | 16.84 | 13.54 | 3.30 | Decrease | 37(70) |
| United States of America | 0.86 | 19.01 | 10.11 | 8.90 | Decrease | 117(100) |
| United States Virgin Islands | 0.82 | 23.48 | 10.77 | 12.71 | Decrease | 165(156) |
| Uruguay | 0.72 | 15.12 | 10.75 | 4.36 | Increase | 57(52) |
| Uzbekistan | 0.66 | 10.82 | 10.82 | 0.00 | Decrease | 1(6) |
| Vanuatu | 0.47 | 28.35 | 13.45 | 14.89 | Decrease | 180(187) |
| Venezuela (Bolivarian Republic of) | 0.60 | 22.84 | 11.76 | 11.07 | Increase | 148(151) |
| Viet Nam | 0.63 | 24.55 | 11.40 | 13.15 | Increase | 169(163) |
| Yemen | 0.45 | 25.08 | 13.56 | 11.52 | Decrease | 155(171) |
| Zambia | 0.51 | 19.18 | 13.09 | 6.10 | Increase | 93(103) |
| Zimbabwe | 0.47 | 27.46 | 13.47 | 13.98 | Increase | 172(180) |
